# Comparative Effectiveness of Noninvasive Brain-Computer Interface–Based Interventions for Upper Limb Rehabilitation in Poststroke Hemiplegia: Systematic Review and Network Meta-Analysis of Randomized Controlled Trials

**DOI:** 10.2196/92940

**Published:** 2026-08-28

**Authors:** Jiabin Xu, Yitian Gao, Siqi Xie, Weikang Jiang, Huiqing Zhang, Lin Qiu, Mengxue Jiang, Lanshu Zhou

**Affiliations:** 1 College of Nursing, Shanghai University of Traditional Chinese Medicine, 1200 Cailun Road, Shanghai, 201203, China, 86 21-5132-2259; 2 Key Laboratory of Geriatric Long-term Care, Naval Medical University, Shanghai, China

**Keywords:** brain-computer interface, stroke, upper limb, hemiplegia, rehabilitation, network meta-analysis

## Abstract

**Background:**

Noninvasive brain-computer interface (BCI)–based interventions show promise for poststroke motor recovery. However, the intrinsic complexity of BCI-based interventions limits the determination of their comparative efficacy.

**Objective:**

Guided by the International Classification of Functioning, Disability and Health framework, this review evaluated the effectiveness of BCI-based interventions in poststroke upper limb rehabilitation and identify the optimal intervention.

**Methods:**

We searched PubMed, Cochrane Library, EBSCOhost, Web of Science, Embase, Wiley Online Library,CNKI, Wanfang, VIP, and SinoMed through July 2026. Randomized controlled trials (RCTs) assessing BCI-based interventions for poststroke upper limb rehabilitation were included. Outcomes were body functions and structures (Fugl-Meyer Assessment of Upper Extremity [FMA-UE]) and activities and participation (Action Research Arm Test [ARAT], Wolf Motor Function Test [WMFT], and Modified Barthel Index [MBI]). Risk of bias was assessed using Cochrane RoB 2, and evidence quality was graded using the Grading of Recommendations, Assessment, Development, and Evaluation framework. We used pairwise meta-analyses to evaluate the overall effectiveness of BCI-based interventions vs controls and network meta-analysis to compare the interventions.

**Results:**

Seventy-two RCTs involving 2906 patients with stroke were included, evaluating 12 BCI-based interventions. Pairwise meta-analyses demonstrated that, compared with control groups, BCI-based interventions improved FMA-UE (mean difference [MD] 5.33, 95% CI 4.28 to 6.38; 95% prediction interval [PI] −1.76 to 12.43), ARAT (MD 5.26, 95% CI 3.90 to 6.62; 95% PI 0.41 to 10.11), WMFT (MD 7.25, 95% CI 5.06 to 9.44; 95% PI 0.71 to 13.79), and MBI (MD 8.18, 95% CI 6.04 to 10.32; 95% PI −1.87 to 18.23). Network meta-analysis revealed that BCI-motor imagery-transcutaneous electrical acupoint stimulation (BCI-MI-TEAS) achieved the highest surface under the cumulative ranking curve (SUCRA; 95.5%) in improving FMA-UE. For ARAT, BCI-MI–end-effector robots and transcranial direct current stimulation (tDCS; 86.3%) alongside BCI-MI-TEAS (86.3%) yielded the highest SUCRA. BCI-MI–exoskeleton robot showed the highest SUCRA for WMFT (92.7%), whereas BCI-MI-TEAS (85.3%) and BCI-MI–exoskeleton robot (81.7%) ranked highest for MBI. The evidence quality ranged from very low to high across these interventions.

**Conclusions:**

This study represents the first network meta-analysis comparing the efficacy of different BCI-based interventions. Unlike previous reviews, interventions were categorized by experimental paradigms, external feedback devices, and adjunctive noninvasive brain stimulation, to enable clinically meaningful comparisons. Overall, BCI-based interventions significantly improved poststroke upper limb rehabilitation. Among evaluated interventions, BCI-MI-TEAS demonstrated the most performance across body functions, structures, and activities and participation, whereas BCI-MI–end-effector robot + tDCS showed advantages for fine motor dexterity and BCI-MI–exoskeleton robot improved activities of daily living.Given low to moderate evidence certainty and substantial heterogeneity, these findings remain exploratory. High-quality trials are needed to establish the clinical utility of these interventions.

## Introduction

Stroke has become one of the leading causes of death worldwide. The global prevalence, incidence, and mortality rates for stroke are 1099.3, 141.6, and 87.5 per 100,000 persons, respectively [1]. With advancements in treatment modalities and medical resources, patients with stroke are now experiencing longer survival times [2]. However, mitigating long-term disability and enhancing quality of life remain significant challenges in stroke management. Among the many sequelae of stroke, upper limb hemiplegia is the most prevalent, affecting nearly two-thirds of survivors [3]. This disability limits independence in activities of daily living (ADL) and leads to secondary complications [4,5]. Restoring upper limb motor function has thus become a primary goal in stroke rehabilitation.

Numerous rehabilitation therapies have demonstrated effectiveness in restoring upper limb function, ranging from conventional motor therapies [6-8] to robot-assisted interventions [9-11]. Conventional therapies require residual motor function, limiting their applicability in patients with severe paralysis. Robot-assisted interventions provide an alternative by facilitating repetitive and intense movements; yet, these passive exercises often constrain their efficacy in promoting cortical neuroplasticity [12]. Motor recovery following a stroke relies heavily on neuroplasticity, the brain’s ability to reorganize and form new neural connections [13]. Therefore, there is a critical need to explore therapeutic interventions that can effectively facilitate both limb functional recovery and neuroplasticity.

Brain-computer interface (BCI)–based interventions bridge peripheral limb rehabilitation with central neuroplasticity by decoding real-time neural activity into functional control commands while simultaneously delivering sensory feedback, including motor, tactile, and visual inputs [14,15]. Through this bidirectional communication, BCI systems can strengthen sensorimotor pathways, providing meaningful rehabilitative benefits for patients with neurological disorders and hemiparesis [16]. BCI-based interventions increasingly integrate active stimulation modalities to synergistically target damaged neural circuits and further enhance these rehabilitative outcomes [17]. While BCI technologies have rapidly advanced across invasive, semi-invasive, and noninvasive modalities, noninvasive systems are most widely adopted in rehabilitation due to their safety, portability, and cost-effectiveness [18]. Typical noninvasive BCI-based interventions comprise 4 core components, including signal acquisition, feature extraction and classification, command translation, and external feedback devices [19,20]. Currently, such interventions are widely applied to poststroke upper limb rehabilitation, with accumulating evidence demonstrating significant therapeutic benefits [21,22].

Despite the clinical promise of noninvasive BCI-based interventions, existing reviews predominantly focus on isolated motor impairment [21,23,24]. Upper limb function serves as a cornerstone of physical capacity, and its recovery is pivotal for enhancing individual activities and participation [25]. Rehabilitation research should extend beyond the mere improvement in local motor impairments to prioritize the restoration of patients’ social reintegration. To effectively bridge the gap between biological recovery and functional independence, the International Classification of Functioning, Disability and Health (ICF) framework provides a robust classification system for rehabilitation assessment [26]. By mapping clinical outcomes across body functions, body structures, activities, and participation, this framework facilitates the development of patient-centered and comprehensive evaluation systems. However, translating this potential into consistent clinical efficacy remains elusive. While meta-analyses have reported significant improvements in upper limb motor function, these gains often fail to achieve the minimal clinically important difference (MCID) [21,24]. Furthermore, significant contradictions exist across clinical trials, with some indicating that BCI-based interventions lack superiority over standard care [27-29]. This complexity is rooted in the inherent diversity of BCI-based interventions, which comprise a wide array of experimental paradigms, feedback devices, training intensities, and multisensory integration strategies [21,24]. This technical diversity drives significant heterogeneity in clinical outcomes, thereby obscuring the consensus on optimal treatment strategies. In this context, network meta-analysis provides a powerful framework for comparing multiple interventions simultaneously [30,31].

Therefore, this study establishes a comprehensive evaluation guided by the ICF framework, pursuing three specific aims: (1) to evaluate the efficacy of BCI-based interventions compared with control groups for poststroke upper limb rehabilitation across body functions, body structures, activities, and participation; (2) to compare the relative efficacy among distinct BCI-based interventions and identify optimal interventions by analyzing various combinations of BCI experimental paradigms, external feedback devices, and adjunctive noninvasive brain stimulation; and (3) to provide multidimensional, stratified guidance for clinical practice and future research directions.

## Methods

### Registration and Protocol

This systematic review protocol was registered with PROSPERO (CRD420251155441). The initial protocol encompassed upper limb, lower limb, and balance recovery as primary outcomes. The subsequent literature search revealed that the available data for lower limb and balance recovery were insufficient to support a network meta-analysis. The study protocol was amended in June 2026 to align with the final implementation plan, ensuring optimal feasibility. The implementation and reporting of this systematic review strictly follow the PRISMA (Preferred Reporting Items for Systematic Reviews and Meta-Analyses) 2020 statement (Checklist 1) [32], and the PRISMA-S (Preferred Reporting Items for Systematic Reviews and Meta-Analyses literature search extension) guideline (Checklist 2) [33] was adopted for the reporting of the search strategy.

### Eligibility Criteria

The search strategy followed the PICOS (population, intervention, comparison, outcome, and study design) principle of evidence-based medicine.

- Population (P): patients aged ≥18 years with a stroke [34]; all participants exhibited limb motor impairment.
- Interventions (I): noninvasive BCI-based interventions; each unique combination of BCI experimental paradigm, external feedback device, and adjunctive noninvasive brain stimulation was defined as a distinct intervention. Details regarding these specific interventions are provided in Table S1 in Multimedia Appendix 1.
- Comparisons (C): conventional rehabilitation therapies (eg, physical therapy, occupational therapy, or usual care), sham interventions, or BCI-based interventions
- Outcomes (O): the Fugl-Meyer Assessment of Upper Extremity (FMA-UE) [35] was used to evaluate body functions and structures; the Action Research Arm Test (ARAT) [36], the Wolf Motor Function Test (WMFT) [37], and the Modified Barthel Index (MBI) [38] were used to evaluate body activities and participation. Specifically, ARAT assesses grasping, gripping, pinching, and gross motor functions; WMFT evaluates upper limb motor function and movement quality through single-joint, multijoint, and functional activities; and MBI reflects an individual’s ability to live independently in domestic and community settings.
- Study design (S): randomized controlled trial (RCT)

The search was conducted without language restrictions. This study excluded studies that were single-arm studies, conference abstracts, letters to the editor, or study protocols that lacked adequate data for extraction. Studies were also excluded if the outcomes of interest were not assessed or if the required data were not reported.

### Information Sources and Search Strategy

A comprehensive search was performed across PubMed, Cochrane Library, EBSCOhost, Web of Science, Embase, Wiley Online Library, China National Knowledge Infrastructure (CNKI), Wanfang Data, VIP Information Chinese Periodical Service Platform, and Chinese Biomedical Literature Database from their inception to July 17, 2026, using the official web interface of each database. For international databases, MeSH terms (eg, stroke, hemiplegia, and brain-computer interfaces) and Emtree terms (eg, cerebrovascular accident, hemiplegia, and noninvasive brain-computer interface) were combined with free-text keywords (eg, electroencephalography [EEG]-based, P300-based, and motor imagery). For the Chinese databases, the search strategy was optimized using discipline-specific terms to align with its indexing framework. The search strategy was developed de novo through team discussion and finalized in consultation with a professional librarian. Furthermore, backward and forward citation searching was manually performed based on the reference lists and citing articles of all eligible studies. The full search strategy is provided in Multimedia Appendix 2.

### Selection Process

Two reviewers (JX and SX) independently imported all records into EndNote (version 21; Clarivate) and removed duplicates. Titles and abstracts were then screened independently according to the eligibility criteria, followed by full-text screening of the remaining studies. Any disagreements were resolved through discussion, and a third reviewer (YG) was consulted when necessary to reach a consensus.

### Data Collection Process and Data Items

Data extraction was performed independently by 2 researchers (JX and SX), referencing the Cochrane Handbook guidelines to record information. No automation tools were used. Disagreements were resolved through discussion or consultation with a third reviewer (YG). Five corresponding authors of the included studies were contacted to request clarification or additional information when data were missing. The extracted data mainly included (1) general information (title, first authors, publication year, and country of publication); (2) demographic characteristics (sample size, age, sex, and disease duration); (3) BCI-based intervention details (BCI experimental paradigm, external auxiliary device, adjunctive noninvasive brain stimulation, and type of primary multisensory feedback); (4) training protocol parameters (session duration, frequency, and total period); (5) outcome data, specifically FMA-UE, ARAT, WMFT, and MBI measured post intervention, for which means and SDs were extracted.

### Risk-of-Bias Assessment

The risk of bias of the included studies was assessed independently by 2 reviewers (SX and WJ). Any discrepancies between the reviewers were resolved through discussion with a third reviewer (JX). The risk of bias of the included studies was assessed using the revised Cochrane RoB 2 tool [39]. Five domains were considered during the assessment process: bias arising from the randomization process, bias due to deviations from intended interventions, bias due to missing outcome data, bias in the measurement of the outcome, and bias in the selection of the reported result.

### Data Synthesis and Analysis

To comprehensively evaluate both the overall effectiveness of BCI-based interventions and the comparative efficacy of these specific interventions, analyses were performed sequentially. First, a pairwise meta-analysis was performed to synthesize available direct evidence, with the primary analysis estimating the overall effectiveness of BCI-based interventions vs control conditions and assessing between-study heterogeneity. Subsequently, a frequentist network meta-analysis was performed to synthesize both direct and indirect evidence, allowing the simultaneous comparison of individual intervention strategies and estimation of their relative rankings. All statistical analyses were performed using R software (version 4.5.1; R Foundation for Statistical Computing).

Following Cochrane guidance, when multiple comparisons from a multiarm trial were included in the same pairwise meta-analysis, the sample size of the shared control group was divided, while its mean and SD remained unchanged. In the network meta-analysis, the original multiarm structure was retained, with correlations among comparisons accounted for using an appropriate multiarm adjustment [40,41]. Potentially overlapping reports were cross-checked, and multiple reports from the same trial were treated as a single study to prevent double counting.

### Pairwise Meta-Analysis

Given the expected clinical and methodological heterogeneity across study populations, intervention characteristics, and outcome measures, pooled mean differences (MDs) between the intervention and control groups with 95% CIs were calculated using a random-effects model with the Hartung-Knapp adjustment to provide robust variance estimation when the number of studies was limited and between-study heterogeneity was substantial [42,43].

Between-study variance (τ^2^) was estimated using the restricted maximum likelihood method [44]. Statistical heterogeneity was assessed using Cochran Q statistic, τ^2^, and *I*^2^ statistic. Prediction intervals (PIs) were calculated for the pairwise meta-analyses to estimate the expected range of treatment effects in future settings and to ascertain whether substantial heterogeneity warranted further investigation [45-47]. To account for anticipated clinical and methodological heterogeneity, prespecified subgroup analyses were subsequently conducted to explore potential sources of heterogeneity according to categorical variables, including the type of primary multisensory feedback and training protocol parameters. Meta-regression analyses were additionally performed for continuous variables, including mean participant age, proportion of male participants, and publication year as a proxy for the development of BCI technology.

### Network Meta-Analysis

Following the estimation of the overall intervention effects, separate network meta-analyses were performed for each outcome to compare and rank individual BCI-based interventions. Network meta-analysis was conducted within a frequentist framework [48]. Network geometry was visualized using network plots, in which nodes represented competing interventions and control groups, while edges represented direct pairwise comparisons. Node sizes were proportional to the total sample sizes, and edge thicknesses corresponded to the numbers of studies informing each comparison. Results were synthesized based on all possible pairwise comparisons, incorporating direct, indirect, and mixed evidence. The surface under the cumulative ranking curve (SUCRA) was used to estimate and rank the hierarchy of the interventions [49]. Comparative results were presented using league tables.

Local inconsistency was evaluated using the node-splitting method [50]. Global inconsistency was assessed using the design-by-treatment interaction model [51]. To evaluate the transitivity assumption, clinical and methodological characteristics were synthesized and compared across specific interventions for each outcome to ensure clinical homogeneity. These characteristics included patient demographics (eg, age and time since stroke onset), specific baseline functional status (eg, FMA-UE, ARAT, WMFT, and MBI), and intervention protocols (eg, type of primary multisensory feedback, session duration, and training frequency).

### Sensitivity Analysis

Sensitivity analyses were performed to test the robustness of the findings. For the pairwise meta-analyses, leave-one-out sensitivity analyses were conducted by sequentially excluding one study at a time to evaluate the stability of the pooled effect sizes. For the network meta-analysis, further sensitivity analyses were conducted by excluding studies judged to have an overall high risk of bias according to the Cochrane RoB 2 tool, with network effect sizes and SUCRA rankings recalculated accordingly.

### Reporting Biases and Small-Study Effects

Potential small-study effects and publication bias were evaluated for individual direct pairwise comparisons comprising 10 or more studies. These assessments were performed visually through the inspection of standard funnel plot symmetry and statistically via Egger linear regression test and Begg rank correlation test [52,53]. For the network meta-analysis, comparison-adjusted funnel plots were generated and visually inspected to qualitatively assess potential network-level asymmetry across different treatment comparisons, following the methodological framework recommended by Cochrane [54]. Recognizing that funnel plot asymmetry can stem from clinical or methodological heterogeneity, small-study effects, and publication bias, the trim-and-fill method was applied specifically to individual pairwise meta-analyses where substantial asymmetry was observed, aiming to estimate potentially missing studies and provide a more conservative adjusted effect estimate.

### Certainty Assessment

Two researchers (JX and YG) independently appraised the quality of evidence for each pairwise comparison regarding outcomes such as FMA-UE, ARAT, WMFT, and MBI, which were evaluated using the Grading of Recommendations, Assessment, Development, and Evaluation (GRADE) framework. Discussion with a third researcher (SX) was conducted to resolve disagreements. The certainty of evidence was classified into 4 levels (high, moderate, low, or very low) by evaluating 5 standard domains: risk of bias, inconsistency, indirectness, imprecision, and publication bias [55].

## Results

### Study Selection

**Figure 1.**
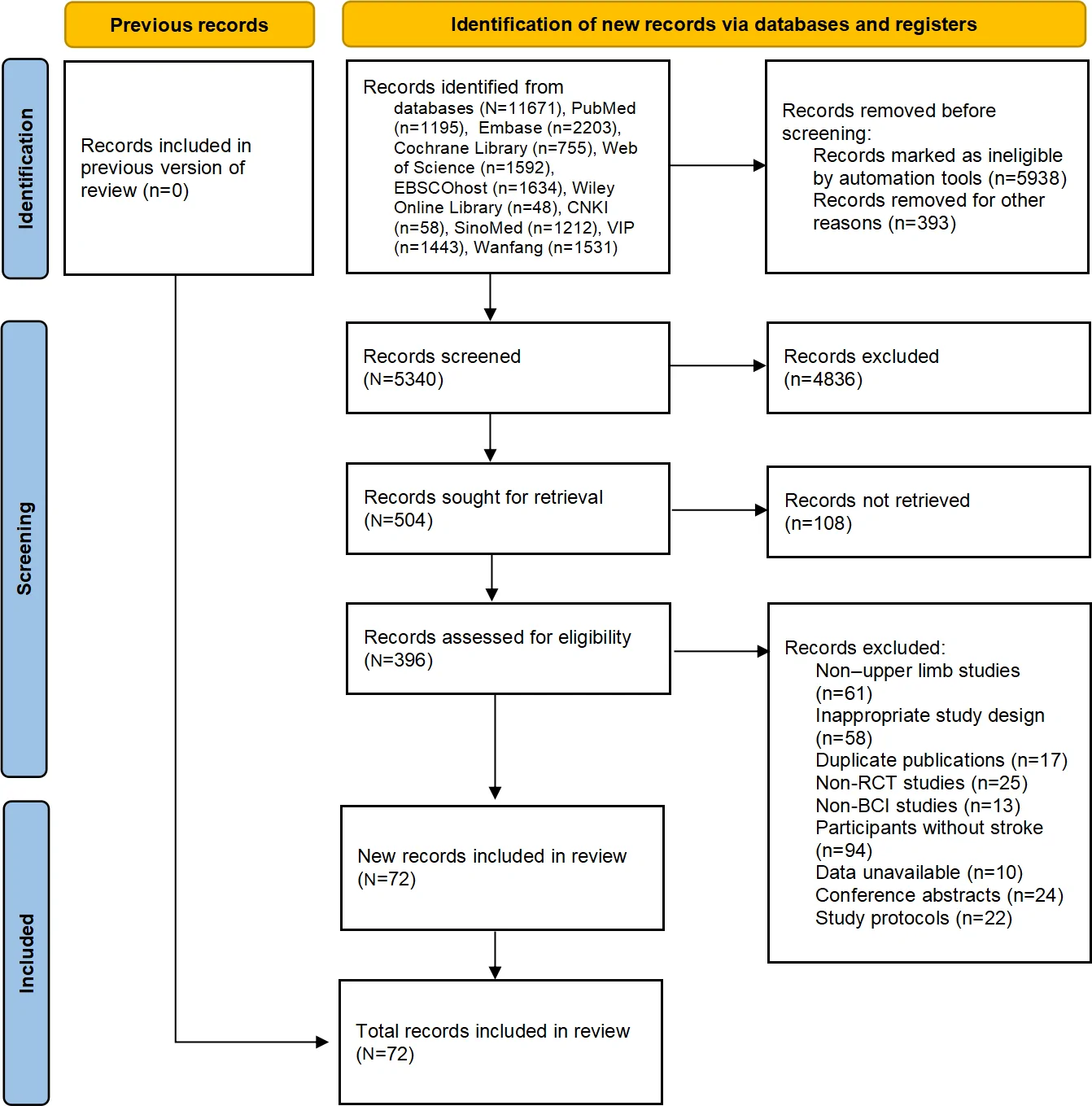
PRISMA flowchart of the study selection and literature screening process. BCI: brain-computer interface; CNKI: China National Knowledge Infrastructure; RCT: randomized controlled trial; SinoMed: Chinese Biomedical Literature Database.

After searching the database and removing duplicate articles, 11,671 articles were potentially eligible. Full texts of 396 studies were retrieved for eligibility assessment. Ultimately, a total of 72 RCTs were finally selected. The detailed selection and exclusion process is documented in the PRISMA flowchart (Figure 1).

### Study Characteristics

The included studies were published between 2013 and 2025 and were conducted in China, Singapore, Germany, Mexico, Russia, Korea, Thailand, Italy, Japan, Brazil, Switzerland, Austria, and Denmark. A total of 72 RCTs involving 2906 participants were included. Sample sizes ranged from 9 to 296 participants, with most participants aged between 50 and 65 years and stroke duration ranging from 0.41 to 58 months. All included studies used electroencephalography-based BCI systems. The BCI paradigms comprised motor imagery (MI), steady-state visual evoked potentials (SSVEP), and P300 event–related potentials (ERP). External devices included end-effector robots, exoskeleton robots, soft robotic gloves, functional electrical stimulation (FES), and transcutaneous electrical acupoint stimulation (TEAS). Adjunctive noninvasive brain stimulation consisted of transcranial direct current stimulation (tDCS) and repetitive transcranial magnetic stimulation (rTMS). Among all evaluated interventions, BCI-MI-FES was the most frequently investigated intervention (25/72, 34.72%), followed by BCI-MI–exoskeleton robot (18/72, 25%) and BCI-MI–end-effector robot (7/72, 9.72%). The number of identified studies is listed in Table 1. Detailed information about these intervention combinations is provided in Table S2 in Multimedia Appendix 1. To evaluate the transitivity assumption, baseline demographic, clinical, and intervention characteristics were compared across intervention categories. Some differences were observed among the groups, as presented in Table S3 in Multimedia Appendix 1.

**Table 1.** Classifications of brain-computer interface (BCI)–based interventions and the number of identified studies (N=72).

Code	Treatment	Studies, n (%)	Intervention (n)	Comparator (n)
A	BCI-ERP-FES^a^	1 (1.39)	7	7
B	BCI-MI^b^	6 (8.33)	95	89
C	BCI-MI–end-effector robot	7 (9.72)	89	95
D	BCI-MI–end-effector robot + tDCS^f^	3 (4.17)	39	68
E	BCI-MI–exoskeleton robot	18 (25)	371	311
F	BCI-MI–exoskeleton robot + tDCS	1 (1.39)	15	13
G	BCI-MI-FES^e^	25 (34.72)	641	633
H	BCI-MI-FES+ rTMS^g^	2 (2.78)	39	41
I	BCI-MI–soft robotic glove	4 (5.56)	65	79
J	BCI-MI-TEAS^d^	2 (2.78)	67	67
K	BCI-SSVEP–exoskeleton^c^ robot	1 (1.39)	18	18
L	BCI-SSVEP–soft robotic glove	2 (2.78)	20	19
	Overall	72 (100)	1466	1440

a ERP: P300 event–related potentials.

b MI: motor imagery.

c SSVEP: steady-state visual evoked potentials.

d TEAS: transcutaneous electrical acupoint stimulation.

e FES: functional electric stimulation.

f tDCS: transcranial direct current stimulation.

g rTMS: repetitive transcranial magnetic stimulation.

### Risk of Bias in Included Studies

**Figure 2.**
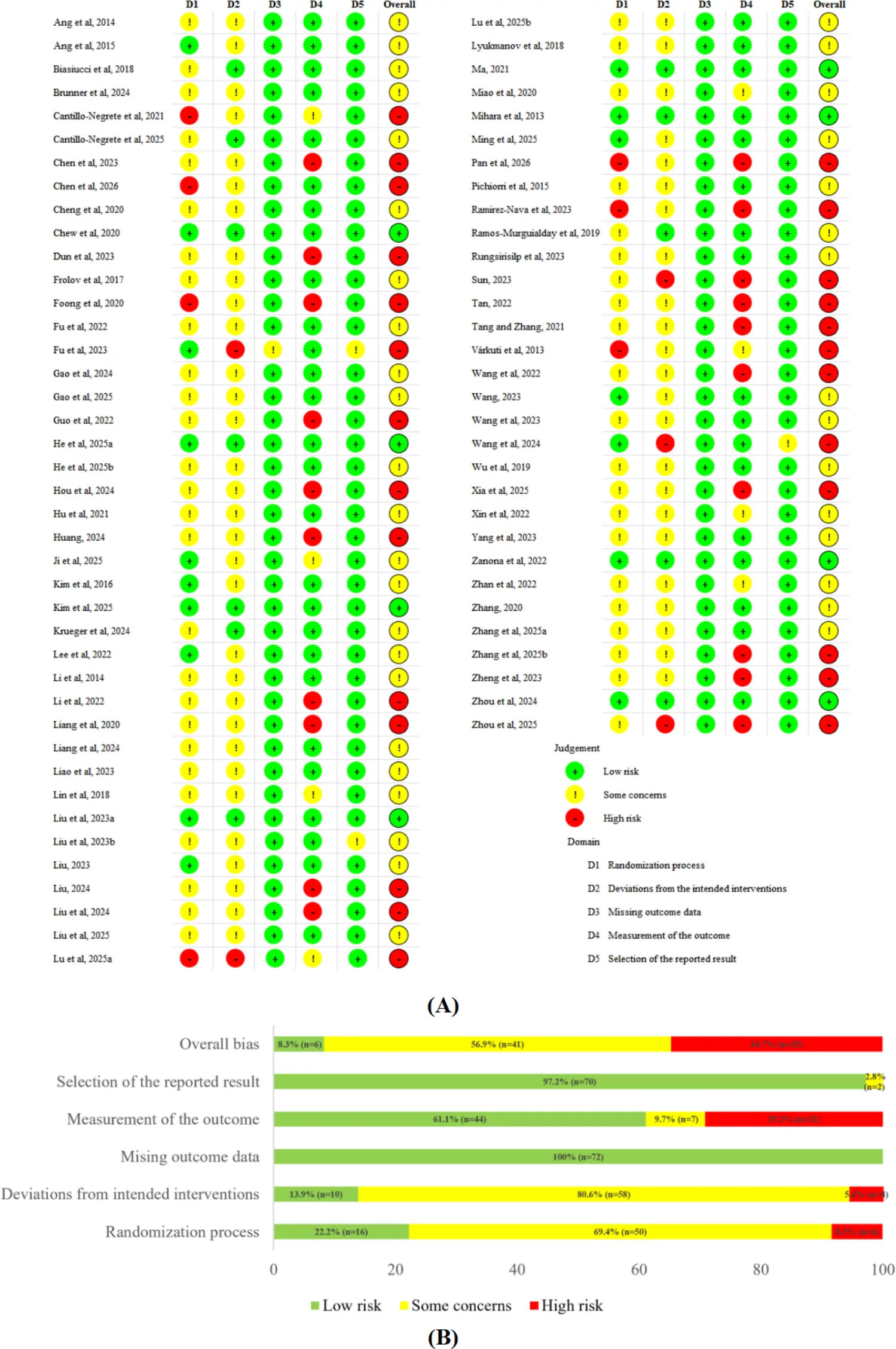
Risk-of-bias assessment of included randomized controlled trials using the Cochrane RoB 2 tool. (A) Study-level judgments across the 5 RoB 2 domains (D1-D5) and (B) domain-level summary across all included studies [27,29,56-125]. "He et al, 2025a" refers to [71], and "He et al, 2025b" refers to [73]; “Liu et al, 2023a” refers to [107], and “Liu et al, 2023b” refers to [87]; Lu et al, 2025a” refers to [58] and “Lu et al, 2025b” refers to [75]; and "Zhang et al, 2025a" refers to [119], and "Zhang et al, 2025b" refers to [93].

Among the 72 included RCTs, 8 (11.11%) were judged to have a low overall risk of bias, 38 (52.78%) were judged to have some concerns, and 26 (36.11%) were classified to have a high risk of bias. Overall and domain-specific assessments are presented in Figure 2.

### Pairwise Meta-Analysis

The pairwise meta-analysis evaluated the overall effectiveness of pooled BCI-based interventions compared with the control group. Significant improvements were observed across all 4 clinical outcomes. For FMA-UE, 69 of the 72 RCTs (95.83%), involving 2760 of 2906 participants (94.98%), showed a pooled mean difference vs control groups of 5.33 (95% CI 4.28 to 6.38; 95% PI −1.76 to 12.43; *I*^2^=89.12%; *P*<.001). For ARAT, 26 of the 72 RCTs (36.11%), comprising 1191 of 2906 participants (40.98%), showed a pooled mean difference vs control groups of 5.26 (95% CI 3.90 to 6.62; 95% PI 0.41 to 10.11; *I*^2^=40.60%; *P*<.001). For WMFT, 16 of the 72 RCTs (22.22%), involving 1000 of 2906 participants (34.41%), showed a pooled mean difference vs control groups of 7.25 (95% CI 5.06 to 9.44; 95% PI 0.71 to 13.79; *I*^2^=50.78%; *P*<.001). For MBI, 30 of the 72 RCTs (41.67%), involving 1343 of 2906 participants (46.22%), showed a pooled mean difference vs control groups of 8.18 (95% CI 6.04 to 10.32; 95% PI −1.87 to 18.23; *I*^2^=88.12%; *P*<.001). Detailed findings for the comparisons between the BCI-based interventions and control groups are presented in Figures S1 and S2 in Multimedia Appendix 1. However, because this analysis estimated only the overall effectiveness of pooled BCI-based interventions, a subsequent network meta-analysis was conducted to compare the relative efficacy and ranking of these specific interventions.

### Network Meta-Analysis Results

Following the pairwise meta-analysis, a network meta-analysis was performed by integrating direct and indirect evidence to compare the relative efficacy of different BCI-based interventions, with treatment hierarchies and rankings determined using SUCRA probabilities.

**Figure 3.**
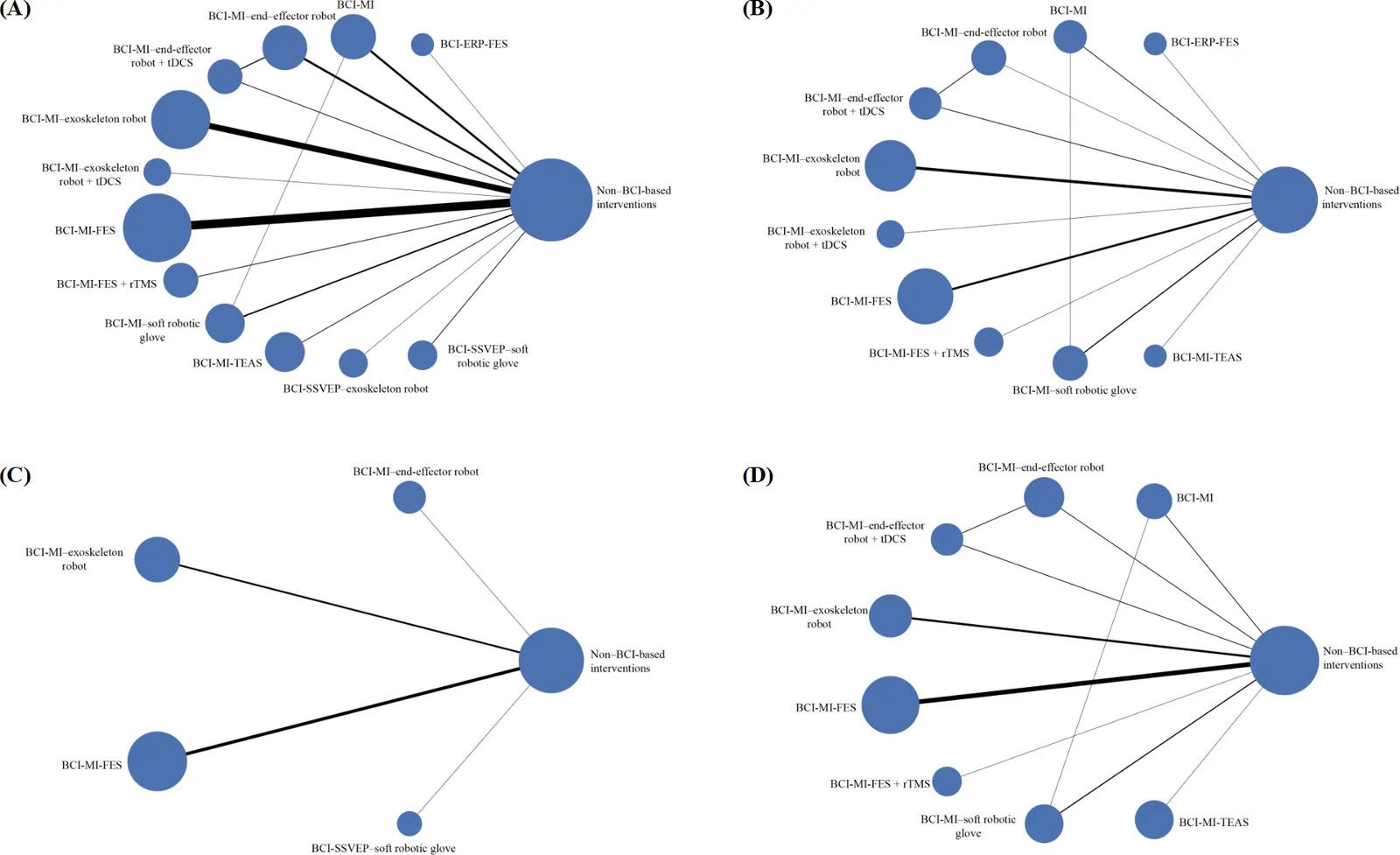
Network structure diagrams for randomized controlled trials of BCI-based interventions among poststroke patients, by outcome: (A) Fugl-Meyer Assessment-Upper Extremity, (B) Action Research Arm Test, (C) Wolf Motor Function Test, and (D) Modified Barthel Index. BCI: brain-computer interface; ERP: P300 event–related potentials; FES: functional electric stimulation; MI: motor imagery; rTMS: repetitive transcranial magnetic stimulation; SSVEP: steady-state visual evoked potentials; tDCS: transcranial direct current stimulation; TEAS: transcutaneous electrical acupoint stimulation.

**Figure 4.**
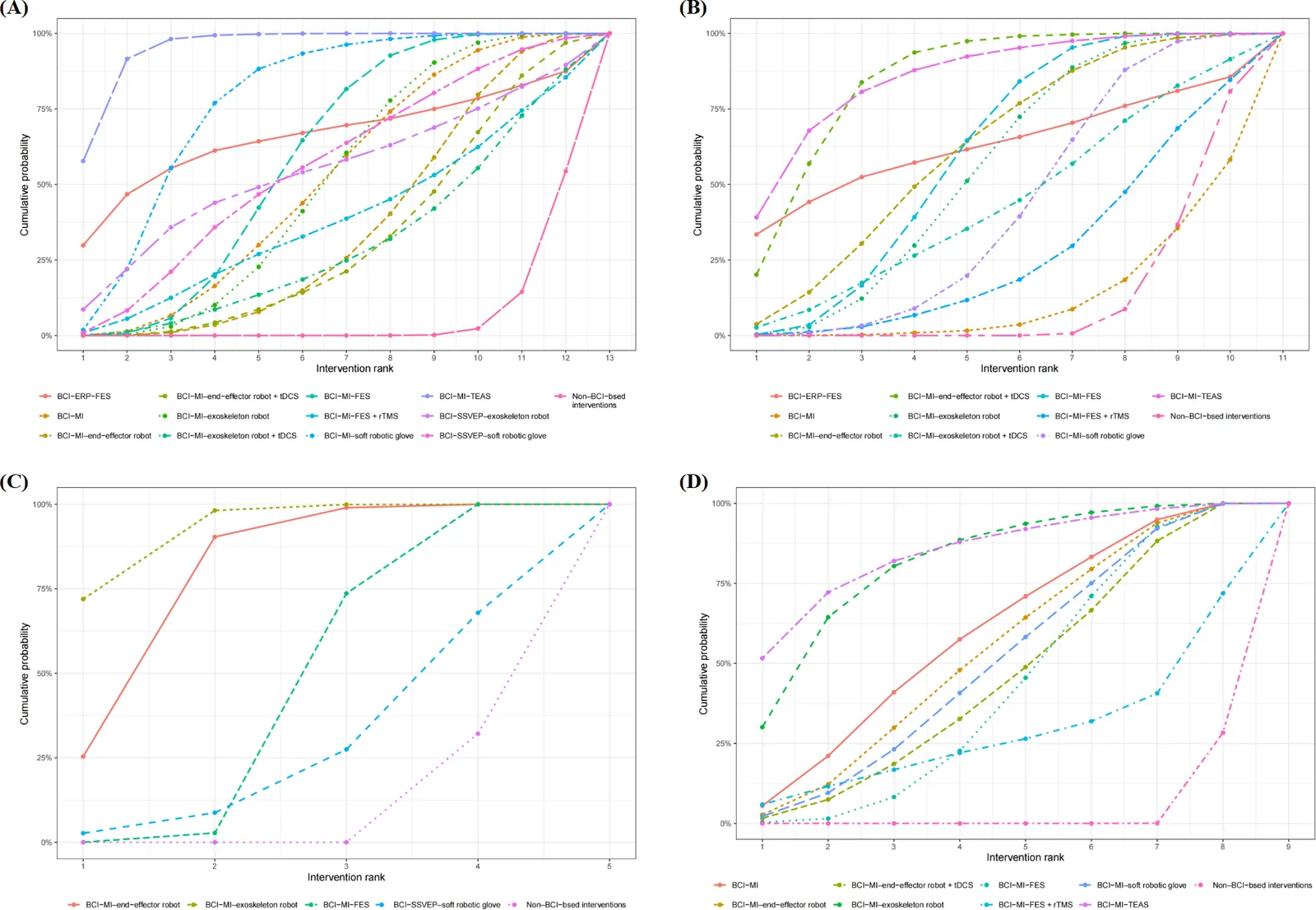
Surface under the cumulative ranking curve of outcome indicators: (A) Fugl-Meyer Assessment-Upper Extremity, (B) Action Research Arm Test, (C) Wolf Motor Function Test, and (D) Modified Barthel Index. BCI: brain-computer interface; ERP: P300 event–related potentials; Exo-RT: exoskeleton robot; FES: functional electric stimulation; MI: motor imagery; rTMS: repetitive transcranial magnetic stimulation; SSVEP: steady-state visual evoked potentials; tDCS: transcranial direct current stimulation; TEAS: transcutaneous electrical acupoint stimulation.

For FMA-UE, the 12 BCI-based interventions formed 14 direct comparisons and 2 closed loops (Figure 3). Compared with non–BCI-based interventions (Table 2), significant improvements were observed for BCI-MI-TEAS (MD 12.87, 95% CI 8.20 to 17.54), BCI-MI–soft robotic glove (MD 7.91, 95% CI 4.42 to 11.40), BCI-SSVEP–soft robotic glove (MD 5.71, 95% CI 0.11 to 11.32), BCI-MI-FES (MD 5.64, 95% CI 3.96 to 7.32), BCI-MI (MD 5.11, 95% CI 2.13 to 8.08), BCI-MI–exoskeleton robot (MD 5.03, 95% CI 2.89 to 7.18), and BCI-MI–end-effector robot (MD 3.79, 95% CI 0.81 to 6.77) (Table 2). According to SUCRA probabilities, the 3 highest-ranked interventions were BCI-MI-TEAS (95.5%), BCI-MI–soft robotic glove (77.5%), and BCI-MI-FES (58.3%). All BCI-based interventions achieved higher SUCRA values than non–BCI-based interventions (5.9%); the specific SUCRA values for all evaluated interventions are detailed in Figure S4A in Multimedia Appendix 1, and the corresponding cumulative ranking probability curves are presented in Figure 4.

**Table 2.** League table for the Fugl-Meyer Assessment-Upper Extremity score showing mean differences and their 95% CIs among BCI-based^a^ interventions.

Treatment	BCI-ERP-FES^b^^c^^,^	BCI-MI^d^	BCI-MI–end-effector robot	BCI-MI–end-effector robot + tDCS^e^	BCI-MI–exoskeleton robot	BCI-MI–exoskeleton robot + tDCS	BCI-MI-FES	BCI-MI-FES + rTMS^f^	BCI-MI–soft robotic glove	BCI-MI-TEAS^g^	BCI-SSVEP–exoskeleton^h^ robot	BCI-SSVEP–soft robotic glove	Non–BCI-based interventions
BCI-ERP-FES	—^i^	—	—	—	—	—	—	—	—	—	—	—	—
BCI-MI	−3.70 (−20.89 to 13.48)	—	—	—	—	—	—	—	—	—	—	—	—
BCI-MI–end-effector robot	−5.02 (−22.20 to 12.17)	−1.31 (−5.52 to 2.90)	—	—	—	—	—	—	—	—	—	—	—
BCI-MI–end-effector robot + tDCS	−5.58 (−22.93 to 11.77)	−1.88 (−6.73 to 2.98)	−0.57 (−4.12 to 2.99)	—	—	—	—	—	—	—	—	—	—
BCI-MI–exoskeleton robot	−3.78 (−20.83 to 13.28)	−0.07 (−3.74 to 3.60)	1.24 (−2.43 to 4.91)	1.81 (-2.59 to 6.20)	—	—	—	—	—	—	—	—	—
BCI-MI–exoskeleton robot + tDCS	−6.02 (−23.91 to 11.87)	−2.32 (−8.83 to 4.20)	−1.00 (−7.52 to 5.51)	−0.44 (−7.39 to 6.51)	−2.24 (−8.42 to 3.93)	—	—	—	—	—	—	—	—
BCI-MI-FES	−3.17 (−20.18 to 13.83)	0.53 (−2.89 to 3.95)	1.84 (-1.58 to 5.27)	2.41 (-1.78 to 6.60)	0.60 (−2.12 to 3.33)	2.85 (−3.19 to 8.88)	—	—	—	—	—	—	—
BCI-MI-FES + rTMS	−5.33 (−24.02 to 13.36)	−1.63 (−10.10 to 6.85)	−0.31 (−8.79 to 8.16)	0.25 (−8.56 to 9.06)	−1.55 (−9.77 to 6.66)	0.69 (−9.13 to 10.51)	-2.16 (−10.27 to 5.95)	—	—	—	—	—	—
BCI-MI–soft robotic glove	-0.90 (−18.18 to 16.38)	2.80 (−1.40 to 7.01)	4.12 (−0.48 to 8.71)	4.68 (−0.51 to 9.87)	2.87 (−1.22 to 6.97)	5.12 (−1.65 to 11.88)	2.27 (−1.60 to 6.15)	4.43 (−4.24 to 13.10)	—	—	—	—	—
BCI-MI-TEAS	4.06 (−13.49 to 21.62)	7.77 (2.23 to 13.30)	9.08 (3.54 to 14.62)	9.64 (3.60 to 15.69)	7.84 (2.70 to 12.98)	10.08 (2.64 to 17.52)	7.23 (2.27 to 12.20)	9.39 (0.19 to 18.60)	4.96 (−0.87 to 10.79)	—	—		—
BCI-SSVEP–exoskeleton robot	−2.81 (−22.86 to 17.24)	0.89 (−10.27 to 12.06)	2.21 (−8.96 to 13.37)	2.77 (−8.65 to 14.20)	0.97 (−10.01 to 11.94)	3.21 (−9.01 to 15.43)	0.36 (−10.53 to 11.26)	2.52 (−10.85 to 15.89)	−1.91 (−13.22 to 9.40)	−6.87 (−18.60 to 4.86)	—	—	—
BCI-SSVEP–soft robotic glove	−3.10 (−20.92 to 14.73)	0.61 (−5.74 to 6.95)	1.92 (−4.43 to 8.27)	2.49 (−4.31 to 9.28)	0.68 (−5.32 to 6.68)	2.92 (−5.14 to 10.99)	0.08 (−5.78 to 5.93)	2.23 (−7.48 to 11.95)	−2.20 (−8.80 to 4.41)	−7.16 (−14.45 to 0.14)	−0.29 (−12.42 to 11.85)	—	—
Non–BCI-based interventions	−8.81 (−25.73 to 8.11)	-5.11 (−8.08 to −2.13)	-3.79 (−6.77 to -0.81)	-3.23 (−7.07 to 0.61)	−5.03 (−7.18 to -2.89)	−2.79 (−8.58 to 3.00)	−5.64 (−7.32 to −3.96)	−3.48 (−11.41 to 4.45)	−7.91 (−11.40 to −4.42)	−12.87 (−17.54 to −8.20)	−6.00 (−16.76 to 4.76)	−5.71 (−11.32 to −0.11)	—

a BCI: brain-computer interface.

b ERP: P300 event–related potentials.

c FES: functional electric stimulation.

d MI: motor imagery.

e tDCS: transcranial direct current stimulation.

f rTMS: repetitive transcranial magnetic stimulation.

g TEAS: transcutaneous electrical acupoint stimulation.

h SSVEP: steady-state visual evoked potentials.

i Not applicable.

For ARAT, the 10 BCI-based interventions formed 12 direct comparisons and 2 closed loops (Figure 3). Compared with non–BCI-based interventions (Table 3), significant improvements were observed in BCI-MI-TEAS (MD 10.28, 95% CI 2.62 to 17.94), BCI-MI–end-effector robot + tDCS (MD 9.13, 95% CI 4.97 to 13.29), BCI-MI–end-effector robot (MD 6.11, 95% CI 0.56 to 11.66), BCI-MI-FES (MD 5.66, 95% CI 3.07 to 8.25), and BCI-MI–soft robotic glove (MD 3.78, 95% CI 0.38 to 7.17). According to SUCRA probabilities, the 3 highest-ranked interventions were BCI-MI-TEAS (86.3%), BCI-MI–end-effector robot + tDCS (86.2%), and BCI-MI–end-effector robot (SUCRA 61.5%). All BCI-based interventions achieved higher SUCRA values than non–BCI-based interventions (12.3%); the specific SUCRA values for all evaluated interventions are detailed in Figure S4B in Multimedia Appendix 1, and the corresponding cumulative ranking probability curves are presented in Figure 4.

For WMFT, the 4 BCI-based interventions formed 4 direct comparisons without any closed loops (Figure 3). Compared with non–BCI-based interventions (Table 4), significant improvements were observed in BCI-MI–exoskeleton robot (MD 11.43, 95% CI 7.15 to 15.71), BCI-MI–end-effector robot (MD 9.50, 95% CI 4.79 to 14.21), and BCI-MI-FES (MD 5.26, 95% CI 3.17 to 7.35). According to SUCRA probabilities, the 3 highest-ranked interventions were BCI-MI–exoskeleton robot (SUCRA 92.7%), BCI-MI–end-effector robot (78.4%), and BCI-MI-FES (44.3%). All BCI-based interventions achieved higher SUCRA values than non–BCI-based interventions (7.9%); the specific SUCRA values for all evaluated interventions are detailed in Figure S4C in Multimedia Appendix 1, and the corresponding cumulative ranking probability curves are presented in Figure 4.

**Table 3.** League table for the Action Research Arm Test score showing mean differences and their 95% CIs among BCI^a^-based interventions.

Treatment	BCI-ERP^b^-FES^c^	BCI-MI^d^	BCI-MI–end-effector robot	BCI-MI–end-effector robot + tDCS^e^	BCI-MI–exoskeleton robot	BCI-MI–exoskeleton robot + tDCS	BCI-MI-FES	BCI-MI-FES + rTMS^f^	BCI-MI–soft robotic glove	BCI-MI-TEAS^g^	Non–BCI-based interventions
BCI-ERP-FES	—^h^	—	—	—	—	—	—	—	—	—	—
BCI-MI	−7.98 (−27.14 to 11.19)	—	—	—	—	—	—	—	—	—	—
BCI-MI–end-effector robot	−1.29 (−20.39 to 17.81)	6.69 (−1.32 to 14.69)	—	—	—	—	—	—	—	—	—
BCI-MI–end-effector robot + tDCS	1.73 (−17.02 to 20.47)	9.71 (2.59 to 16.82)	3.02 (−1.17 to 7.21)	—	—	—	—	—	—	—	—
BCI-MI–exoskeleton robot	−2.23 (−20.82 to 16.36)	5.75 (−0.95 to 12.45)	−0.94 (−7.44 to 5.57)	−3.95 (−9.33 to 1.42)	—	—	—	—	—	—	—
BCI-MI–exoskeleton robot + tDCS	−3.67 (−23.68 to 16.34)	4.31 (−5.69 to 14.30)	−2.38 (−12.25 to 7.49)	−5.40 (−14.56 to 3.76)	−1.44 (−10.28 to 7.39)	—	—	—	—	—	—
BCI-MI-FES	−1.74 (−20.20 to 16.72)	6.24 (−0.09 to 12.57)	−0.45 (−6.57 to 5.68)	−3.47 (−8.37 to 1.43)	0.49 (−3.79 to 4.76)	1.93 (−6.63 to 10.49)	—	—	—	—	—
BCI-MI-FES + rTMS	−5.73 (−25.06 to 13.60)	2.25 (−6.30 to 10.79)	−4.44 (−12.83 to 3.95)	−7.46 (−15.01 to 0.09)	−3.50 (−10.66 to 3.65)	−2.06 (−12.37 to 8.25)	−3.99 (−10.80 to 2.82)	—	—	—	—
BCI-MI–soft robotic glove	−3.62 (−22.21 to 14.97)	4.36 (−0.91 to 9.62)	−2.33 (−8.84 to 4.17)	−5.35 (−10.72 to 0.02)	−1.40 (−6.20 to 3.41)	0.05 (−8.79 to 8.89)	−1.88 (−6.15 to 2.39)	2.11 (−5.05 to 9.27)	—	—	—
BCI-MI-TEAS	2.88 (−16.94 to 22.70)	10.86 (1.26 to 20.45)	4.17 (−5.29 to 13.63)	1.15 (−7.57 to 9.87)	5.11 (−3.28 to 13.49)	6.55 (−4.64 to 17.74)	4.62 (−3.47 to 12.70)	8.61 (−1.31 to 18.53)	6.50 (−1.88 to 14.88)	—	—
Non–BCI-based interventions	−7.40 (−25.68 to 10.88)	0.58 (−5.20 to 6.35)	−6.11 (−11.66 to −0.56)	−9.13 (−13.29 to −4.97)	−5.17 (−8.57 to −1.77)	−3.73 (−11.89 to 4.43)	−5.66 (−8.25 to −3.07)	−1.67 (−7.97 to 4.63)	−3.78 (−7.17 to −0.38)	−10.28 (−17.94 to −2.62)	—

a BCI: brain-computer interface.

b ERP: P300 event–related potentials.

c FES: functional electric stimulation.

d MI: motor imagery.

e tDCS: transcranial direct current stimulation.

f rTMS: repetitive transcranial magnetic stimulation.

g TEAS: transcutaneous electrical acupoint stimulation.

h Not applicable.

**Table 4.** League table for the Wolf Motor Function Test score showing mean differences and their 95% CIs among BCI-based^a^ interventions.

Treatment	BCI-MI^b^–end-effector robot	BCI-MI–exoskeleton robot	BCI-MI-FES^c^	BCI-SSVEP^d^–soft robotic glove	Non–BCI-based interventions
BCI-MI–end-effector robot	—^e^	—	—	—	—
BCI-MI–exoskeleton robot	1.93 (−4.43 to 8.29)	—	—	—	—
BCI-MI-FES	−4.24 (−9.39 to 0.91)	−6.17 (−10.93 to −1.41)	—	—	—
BCI-SSVEP–soft robotic glove	−7.25 (−18.59 to 4.09)	−9.18 (−20.35 to 1.99)	−3.01 (−13.53 to 7.51)	—	—
Non–BCI-based interventions	−9.50 (−14.21 to −4.79)	−11.43 (−15.71 to −7.15)	−5.26 (−7.35 to −3.17)	−2.25 (−12.56 to 8.06)	—

a BCI: brain-computer interface.

b MI: motor imagery.

c FES: functional electric stimulation.

d SSVEP: steady-state visual evoked potentials.

e Not applicable.

For MBI, the 8 BCI-based interventions formed 10 direct comparisons and 2 closed loops (Figure 3). Compared with non–BCI-based interventions (Table 5), significant improvements were observed in BCI-MI-TEAS (MD 13.86, 95% CI 5.05 to 22.67), BCI-MI–exoskeleton robot (MD 12.44, 95% CI 6.42 to 18.46), BCI-MI (MD 9.40, 95% CI 3.80 to 14.99), BCI-MI–end-effector robot (MD 8.75, 95% CI 3.56 to 13.94), BCI-MI–soft robotic glove (MD 8.38, 95% CI 3.46 to 13.29), BCI-MI–end-effector robot + tDCS (MD 7.85, 95% CI 2.52 to 13.18), and BCI-MI-FES (MD 7.75, 95% CI 4.79 to 10.70). According to SUCRA probabilities, the 3 highest-ranked interventions were BCI-MI-TEAS (85.3%), BCI-MI–exoskeleton robot (81.7%), and BCI-MI (59.3%). All BCI-based interventions achieved higher SUCRA values than non–BCI-based interventions (3.5%); the specific SUCRA values for all evaluated interventions are detailed in Figure S4D in Multimedia Appendix 1, and the corresponding cumulative ranking probability curves are presented in Figure 4.

Overall, the network meta-analyses demonstrated that BCI-MI-TEAS consistently ranked among the most effective interventions across FMA-UE, ARAT, and MBI, whereas BCI-MI–exoskeleton robot showed the greatest efficacy for WMFT.

**Table 5.** League table for the Modified Barthel Index score showing mean differences and their 95% CIs among BCI^a^-based interventions.

Treatment	BCI-MI	BCI-MI^b^–end-effector robot	BCI-MI–end-effector robot + tDCS^c^	BCI-MI–exoskeleton robot	BCI-MI-FES^d^	BCI-MI-FES + rTMS^e^	BCI-MI–soft robotic glove	BCI-MI-TEAS	Non–BCI-based interventions
BCI-MI	—^g^	—	—	—	—	—	—	—	—
BCI-MI–end-effector robot	−0.64 (−8.27 to 6.99)	—	—	—	—	—	—	—	—
BCI-MI–end-effector robot + tDCS	−1.55 (−9.27 to 6.18)	−0.91 (−6.24 to 4.43)	—	—	—	—	—	—	—
BCI-MI–exoskeleton robot	3.04 (−5.17 to 11.26)	3.69 (−4.26 to 11.63)	4.59 (−3.45 to 12.63)	—	—	—	—	—	—
BCI-MI-FES	−1.65 (−7.97 to 4.68)	−1.01 (−6.98 to 4.97)	−0.10 (−6.19 to 5.99)	−4.69 (−11.39 to 2.01)	—	—	—	—	—
BCI-MI-FES + rTMS	−5.40 (−20.27 to 9.47)	−4.75 (−19.48 to 9.97)	−3.85 (−18.62 to 10.92)	−8.44 (−23.47 to 6.59)	−3.75 (−17.84 to 10.34)	—	—	—	—
BCI-MI–soft robotic glove	−1.02 (−7.57 to 5.53)	−0.38 (−7.53 to 6.77)	0.53 (−6.72 to 7.78)	−4.06 (−11.83 to 3.71)	0.63 (−5.11 to 6.36)	4.38 (−10.25 to 19.00)	—	—	—
BCI-MI-TEAS^f^	4.46 (−5.97 to 14.90)	5.11 (−5.12 to 15.33)	6.01 (−4.28 to 16.30)	1.42 (−9.25 to 12.09)	6.11 (−3.18 to 15.40)	9.86 (−6.49 to 26.21)	5.48 (−4.60 to 15.57)	—	—
Non–BCI-based interventions	−9.40 (−14.99 to −3.80)	−8.75 (−13.94 to −3.56)	−7.85 (−13.18 to −2.52)	−12.44 (−18.46 to −6.42)	−7.75 (−10.70 to −4.79)	−4.00 (−17.78 to 9.78)	−8.38 (−13.29 to −3.46)	−13.86 (−22.67 to −5.05)	—

a BCI: brain-computer interface.

b MI: motor imagery.

c tDCS: transcranial direct current stimulation.

d FES: functional electric stimulation.

e rTMS: repetitive transcranial magnetic stimulation.

f TEAS: transcutaneous electrical acupoint stimulation.

g Not applicable.

### Inconsistency Assessment

The validity of the network estimates was evaluated through assessments of global and local inconsistency. Global inconsistency was assessed using the design-by-treatment interaction model. Significant global inconsistency was identified in the FMA-UE network (*P*=.01), whereas no evidence of global inconsistency was observed for the ARAT (*P*=.82) or MBI (*P*=.15) networks (Table 6). Global inconsistency could not be evaluated for WMFT because the corresponding network contained no closed loops. Local inconsistency was further assessed using the node-splitting approach. Significant disagreement between direct and indirect evidence was identified in the FMA-UE network. In contrast, no statistically significant local inconsistency was detected for the ARAT or MBI networks. Node-splitting analysis was not applicable to the WMFT network because no closed loop was available. Detailed results of the inconsistency analyses are presented in Table S5 in Multimedia Appendix 1.

**Table 6.** The results of heterogeneity and inconsistency of brain-computer interface (BCI)–based interventions.

Results	Heterogeneity				Global inconsistency (*P* value)
	*I*^2^ (%)	Q	τ^2^	*P* value	
FMA-UE^a^	89.12	661.99	12.34	<.001	.01
ARAT^b^	40.60	47.13	5.09	.01	.82
WMFT^c^	50.78	30.47	8.24	.01	—^d^
MBI^e^	88.12	277.78	23.28	<.001	.15

a FMA-UE: Fugl-Meyer Assessment-Upper Extremity.

b ARAT: Action Research Arm Test.

c WMFT: Wolf Motor Function Test.

d Not available.

e MBI: Modified Barthel Index.

### Subgroup Analyses and Meta-Regression Results

To investigate potential sources of heterogeneity identified in the pairwise meta-analysis, prespecified subgroup analyses were performed (Figure S3 in Multimedia Appendix 1). For FMA-UE, significant subgroup differences were observed according to session duration (*I*^2^=91.72%; τ^2^=7.68; *P*<.001) and total period (*I*^2^=77.72%; τ^2^=2.17; *P*=.01). Greater improvements were associated with interventions delivered 4 to 5 days per week (MD 6.54, 95% CI 5.44 to 7.65; *P*<.001) and a total period of 2 to 3 weeks (MD 7.62, 95% CI 6.45 to 8.80; *P*<.001). For MBI, training frequency (*I*^2^=74.73%; τ^2^=5.15; *P*=.05), total period (*I*^2^=70.92%; τ^2^=8.18; *P*=.03), and multisensory feedback modalities (*I*^2^=94.66%; τ^2^=16.58; *P*<.001) exhibited substantial subgroup heterogeneity. Notably, a distinct trend toward higher efficacy was clustered in the 60 to 90 minutes per day subgroup (MD 11.82, 95% CI 8.83 to 14.80; *P*<.001), the 2 to 3 weeks subgroup (MD 12.25, 95% CI 8.99 to 15.50; *P*<.001), and the auditory feedback subgroup (MD 12.66, 95% CI 12.36 to 12.96; *P*<.001). No statistically significant subgroup effects were identified for ARAT or WMFT.

Meta-regression analyses demonstrated that mean participant age was significantly associated with treatment effect for FMA-UE (*β*=.038, 95% CI 0.002 to 0.073; *P*=.04). No significant associations were identified for the remaining continuous variables. Detailed meta-regression results are presented in Table S4 in Multimedia Appendix 1.

### Reporting Biases and Small-Study Effects

Potential small-study effects and publication bias for individual direct pairwise comparisons were evaluated using standard funnel plots (Figure S5 in Multimedia Appendix 1). Visual inspection indicated that the effect sizes for ARAT and WMFT were generally symmetrically distributed. In contrast, the funnel plots for FMA-UE and MBI exhibited substantial asymmetry. Egger test confirmed a significant small-study effect for FMA-UE (*P*=.006). In contrast, no significant publication bias was detected for ARAT, WMFT, and MBI using Egger test (all *P*>.05; Table S6 in Multimedia Appendix 1). A trim-and-fill analysis was conducted to assess the impact of potential publication bias on the FMA-UE outcome. After adjusting for 32 potentially missing studies via imputation, the pooled MD for FMA-UE remained robust at 7.15 (95% CI 6.08 to 8.22; *P*<.001).

For the network meta-analyses, comparison-adjusted funnel plots were generated for each outcome (Figure S6 in Multimedia Appendix 1) to qualitatively evaluate potential small-study effects and publication bias across different treatment comparisons. Visual inspection of the comparison-adjusted funnel plots revealed general symmetry across the network comparisons and no substantial asymmetry, which was further supported by nonsignificant statistical tests (all *P*>.05; Table S7 in Multimedia Appendix 1), indicating a low likelihood of publication bias or small-study effects.

### Sensitivity Analysis

Leave-one-out sensitivity analyses (Figure S7 in Multimedia Appendix 1) confirmed the robustness of pairwise meta-analysis results for FMA-UE, ARAT, WMFT, and MBI after sequentially excluding individual trials.

After excluding studies categorized as high risk of bias by the Cochrane RoB 2 tool, global and local inconsistency tests were performed. The *P* values for global inconsistency were .06 for FMA-UE, .82 for ARAT, and .15 for MBI, while all local inconsistency tests yielded *P*>.05 (Table S8 in Multimedia Appendix 1). Subsequently, intervention hierarchies were estimated using SUCRA probabilities. For FMA-UE, BCI-MI-TEAS demonstrated the highest SUCRA probability (98.6%), followed by BCI-MI–soft robotic glove (75.9%). Regarding ARAT, BCI-MI-TEAS ranked highest (91.3%), closely followed by BCI-MI–end-effector robot + tDCS (90.5%). For WMFT, BCI-MI–exoskeleton robot exhibited the highest probability of being ranked first (98.7%) compared to others. For MBI, BCI-MI-TEAS (80.5%) and BCI-MI–exoskeleton robot (77.9%) were the top-ranked interventions. However, the SUCRA probabilities for BCI-MI remained relatively low across FMA-UE (29.4%), ARAT (12%), and WMFT (29.9%), surpassing only the non–BCI-based interventions. The comparative efficacy between all competing interventions is presented in Table S9 in Multimedia Appendix 1, and the corresponding SUCRA rankings are illustrated in Figure S8 in Multimedia Appendix 1.

### Certainty of Evidence

Based on the GRADE framework, the certainty of evidence for all comparisons ranged from very low to high. Although interventions such as BCI-MI-TEAS, BCI-MI–exoskeleton robot, and BCI-MI–end-effector robot may potentially improve limb function in patients with stroke, the low certainty of evidence precludes definitive comparative conclusions. In contrast, the certainty of evidence for BCI-MI-FES was rated as high across the FMA-UE, ARAT, and WMFT outcomes. To enhance methodological transparency, the detailed risk of bias across domains and the corresponding evidence grades are presented in Tables 7–10.

**Table 7.** Grading of Recommendations, Assessment, Development, and Evaluation summary of the quality of the evidence for the Fugl-Meyer Assessment-Upper Extremity.

Certainty assessment						Number of participants		Effect	Certainty
Comparison	Risk of bias^a^	Inconsistency^b^	Indirectness^c^	Imprecision^d^	Other considerations^e^	Intervention^f^ (n)	Comparator^g^ (n)	MD^h^ (95% CI)	
BCI-ERP-FES^i^,^j^ vs non–BCI-based^k^ interventions	Serious	Not serious	Not serious	Very serious	Undetected	9	9	8.81 (−8.11 to 25.73)	⊕ΟOO Very low
BCI-MI^l^ vs non–BCI-based interventions	Not serious	Serious	Serious	Not serious	Undetected	115	108	5.11 (2.81 to 8.08)	⊕⊕OO Low
BCI-MI–end-effector robot vs non–BCI-based interventions	Serious	Serious	Not serious	Not serious	Undetected	69	76	3.79 (0.81 to 6.77)	⊕⊕ΟΟ Low
BCI-MI–end-effector robot + tDCS vs non–BCI-based interventions	Not serious	Very serious	Serious	Serious	Undetected	14	29	3.23 (−0.61 to 7.07)	⊕ΟΟΟ Very low
BCI-MI–end-effector robot + tDCS vs BCI-MI–end-effector robot	Not serious	Serious	Not serious	Serious	Undetected	25	38	0.57 (−2.99 to 4.12)	⊕⊕ΟΟ Low
BCI-MI–exoskeleton robot vs non–BCI-based interventions	Serious	Serious	Not serious	Not serious	Undetected	331	270	5.03 (2.89 to 7.18)	⊕⊕ΟΟ Low
BCI-MI–exoskeleton robot + tDCS^m^ vs non–BCI-based interventions	Not serious	Not serious	Not serious	Serious	Undetected	15	13	2.79 (−3.00 to 8.58)	⊕⊕⊕Ο Moderate
BCI-MI-FES vs non–BCI-based interventions	Not serious	Not serious	Not serious	Not serious	Undetected	607	599	5.64 (3.96 to 7.32)	⊕⊕⊕⊕ High
BCI-MI-FES+ rTMS^n^ vs non–BCI-based interventions	Serious	Serious	Not serious	Serious	Undetected	39	41	3.48 (−4.45 to 11.41)	⊕ΟΟΟ Very low
BCI-MI–soft robotic glove vs non–BCI-based interventions	Not serious	very serious	Not serious	Not serious	Undetected	57	64	7.91 (4.42 to 11.40)	⊕⊕ΟΟ Low
BCI-MI–soft robotic glove vs BCI-MI	Not serious	Serious	Not serious	Serious	Undetected	8	15	−2.80 (−7.01 to 1.40)	⊕⊕ΟΟ Low
BCI-MI-TEAS^o^ vs non–BCI-based interventions	Not serious	Serious	Not serious	Serious	Undetected	67	67	12.87 (8.20 to 17.54)	⊕⊕ΟΟ Low
BCI-SSVEP–exoskeleton^p^ robot vs non–BCI-based interventions	Serious	Not serious	Not serious	Very serious	Undetected	18	18	6.00 (−4.76 to 16.76)	⊕ΟΟΟ Very low
BCI-SSVEP–soft robotic glove vs non–BCI-based interventions	Serious	Not serious	Not serious	Not serious	Undetected	20	19	5.71 (0.11 to 11.32)	⊕⊕⊕Ο Moderate

a Risk of bias: downgraded by 1 level if the majority of evidence was derived from studies at high risk of bias.

b Inconsistency: assessed using node-splitting and design-by-treatment interaction models. Evidence was downgraded by 1 level for significant inconsistency (*P*<.05) or substantial heterogeneity (*I*2≥50%) and by 2 levels for *I*2≥80%.

c Indirectness: downgraded by 1 level if included studies deviated from the predefined population, intervention, comparison, outcome, and study design (PICOS) framework or violated the transitivity assumption.

d Imprecision: downgraded for wide CIs, failure to meet the optimal information size, or estimates failing to meet the minimal clinically important difference. Two levels of downgrading were applied only in cases of very serious imprecision.

e Other considerations: evidence was downgraded by 1 level for evidence of publication bias.

f Intervention: BCI-based combination therapies.

g Comparator: conventional rehabilitation or control.

h MD: mean difference

i ERP: P300 event–related potentials

j FES: functional electric stimulation

k BCI: brain-computer interface

l MI: motor imagery

m tDCS: transcranial direct current stimulation

n rTMS: repetitive transcranial magnetic stimulation

o TEAS: transcutaneous electrical acupoint stimulation

p SSVEP: steady-state visual evoked potentials

**Table 8.** Grading of Recommendations, Assessment, Development, and Evaluation summary of the quality of the evidence for the Action Research Arm Test.

Certainty assessment						Number of participants		Effect	Certainty
Comparison	Risk of bias^a^	Inconsistency^b^	Indirectness^c^	Imprecision^d^	Other considerations^e^	Intervention^f^ (n)	Comparator^g^ (n)	MD^h^ (95% CI)	
BCI-ERP-FES^i^^j^^k^ vs non–BCI-based interventions	Serious	Not serious	Not serious	Very serious	Undetected	9	9	7.40 (−10.88 to 25.68)	⊕ΟΟΟ Very low
BCI-MI^l^ vs non–BCI-based interventions	Not serious	Not serious	Serious	Serious	Undetected	17	15	−0.58 (−6.35 to 5.20)	⊕⊕ΟΟ Low
BCI-MI–end-effector robot vs non–BCI-based interventions	Serious	Not serious	Not serious	Not serious	Undetected	11	11	6.11 (0.56 to 11.66)	⊕⊕⊕Ο Moderate
BCI-MI–end-effector robot + tDCS^m^ vs non–BCI-based interventions	Serious	Not serious	Serious	Not serious	Undetected	14	29	9.13 (4.97 to 13.29)	⊕⊕ΟΟ Low
BCI-MI–end-effector robot + tDCS vs BCI-MI–end-effector robot	Not serious	Not serious	Serious	Serious	Undetected	15	29	−3.02 (−7.21 to 1.17)	⊕⊕ΟΟ Low
BCI-MI–exoskeleton robot vs non–BCI-based interventions	Serious	Not serious	Serious	Not serious	Undetected	190	136	5.17 (1.77 to 8.57)	⊕⊕ΟΟ Low
BCI-MI–exoskeleton robot + tDCS vs non–BCI-based interventions	Not serious	Not serious	Not serious	Serious	Undetected	15	13	3.73 (−4.43 to 11.89)	⊕⊕⊕Ο Moderate
BCI-MI-FES vs non–BCI-based interventions	Not serious	Not serious	Not serious	Not serious	Undetected	267	263	5.66 (3.07 to 8.25)	⊕⊕⊕⊕ High
BCI-MI-FES+ rTMS^n^ vs non–BCI-based interventions	Serious	Not serious	Not serious	Serious	Undetected	19	21	1.67 (−4.63 to 7.97)	⊕⊕ΟΟ Low
BCI-MI–soft robotic glove vs non–BCI-based interventions	Not serious	Very serious	Not serious	Not serious	Undetected	32	39	3.78 (0.38 to 7.17)	⊕⊕ΟΟ Low
BCI-MI–soft robotic glove vs BCI-MI	Not serious	Serious	Not serious	Serious	Undetected	8	15	−2.11 (9.27 to −5.05)	⊕⊕ΟΟ Low
BCI-MI-TEAS^o^ vs non–BCI-based interventions	Not serious	Not serious	Not serious	Serious	Undetected	7	7	10.28 (2.62 to 17.94)	⊕⊕⊕Ο Moderate

a Risk of bias: downgraded by 1 level if the majority of evidence was derived from studies at high risk of bias.

b Inconsistency: assessed using node-splitting and design-by-treatment interaction models. Evidence was downgraded by 1 level for significant inconsistency (*P*<.05) or substantial heterogeneity (*I*2≥50%) and by 2 levels for *I*2≥80%.

c Indirectness: downgraded by 1 level if included studies deviated from the predefined population, intervention, comparison, outcome, and study design (PICOS) framework or violated the transitivity assumption.

d Imprecision: downgraded for wide CIs, failure to meet the optimal information size, or estimates failing to meet the minimal clinically important difference. Two levels of downgrading were applied only in cases of very serious imprecision.

e Other considerations: evidence was downgraded by 1 level for evidence of publication bias.

f Intervention: BCI-based combination therapies.

g Comparator: conventional rehabilitation or control.

h MD: mean difference.

i BCI: brain-computer interface.

j ERP: P300 event–related potentials.

k FES: functional electric stimulation.

l MI: motor imagery.

m tDCS: transcranial direct current stimulation.

n rTMS: repetitive transcranial magnetic stimulation.

o TEAS: transcutaneous electrical acupoint stimulation.

**Table 9.** Grading of Recommendations, Assessment, Development, and Evaluation summary of the quality of the evidence for the Wolf Motor Function Test.

Certainty assessment						Number of participants		Effect	Certainty
Comparison	Risk of bias^a^	Inconsistency^b^	Indirectness^c^	Imprecision^d^	Other considerations^e^	Intervention^f^ (n)	Comparator^g^ (n)	MD^h^ (95% CI)	
BCI^i^-MI^j^–end-effector, robot vs non–BCI-based interventions	Serious	Not serious	Not serious	Not serious	Undetected	30	30	9.50 (4.79 to 14.21)	⊕⊕⊕Ο Moderate
BCI-MI–exoskeleton robot vs non–BCI-based interventions	Serious	Not serious	Not serious	Not serious	Undetected	116	117	11.43 (7.15 to 15.71)	⊕⊕⊕Ο Moderate
BCI-MI-FES^k^ vs non–BCI-based interventions	Not serious	Not serious	Not serious	Not serious	Undetected	346	341	5.26 (3.17 to 7.35)	⊕⊕⊕⊕ High
BCI-SSVEF^l^–soft robotic glove vs non–BCI-based interventions	Serious	Not serious	Not serious	Very serious	Undetected	10	10	2.25 (−8.06 to 12,56)	⊕ΟΟΟ Very low

a Risk of bias: downgraded by 1 level if the majority of evidence was derived from studies at high risk of bias.

b Inconsistency: assessed using node-splitting and design-by-treatment interaction models. Evidence was downgraded by 1 level for significant inconsistency (*P*<.05) or substantial heterogeneity (*I*2≥50%) and by 2 levels for *I*2≥80%.

c Indirectness: downgraded by one level if included studies deviated from the predefined population, intervention, comparison, outcome, and study design (PICOS) framework or violated the transitivity assumption.

d Imprecision: downgraded for wide CIs, failure to meet the optimal information size, or estimates failing to meet the minimal clinically important difference. Two levels of downgrading were applied only in cases of very serious imprecision.

e Other considerations: evidence was downgraded by 1 level for evidence of publication bias.

f Intervention: BCI-based combination therapies.

g Comparator: conventional rehabilitation or control.

h MD: mean difference.

i BCI: brain-computer interface.

j MI: motor imagery.

k FES: functional electric stimulation.

l SSVEP: steady-state visual evoked potentials.

**Table 10.** Grading of Recommendations, Assessment, Development, and Evaluation summary of the quality of the evidence for the Modified Barthel Index.

Certainty assessment						Number of participants		Effect	Certainty
Comparison	Risk of bias^a^	Inconsistency^b^	Indirectness^c^	Imprecision^d^	Other considerations^e^	Intervention^f^ (n)	Comparator^g^ (n)	MD^h^ (95% CI)	
BCI^i^-MI^j^ vs non–BCI-based interventions	Not serious	Not serious	Serious	Serious	Undetected	46	42	9.40 (3.80 to 14.99)	⊕⊕ΟΟ Low
BCI-MI–end-effector robot vs non–BCI-based interventions	Serious	Not serious	Not serious	Not serious	Undetected	41	41	8.75 (3.56 to 13.94)	⊕⊕⊕Ο Moderate
BCI-MI–end-effector robot + tDCS^k^ vs non–BCI-based interventions	Not serious	Serious	Serious	Serious	Undetected	14	29	7.85 (2.52 to 13.18)	⊕ΟΟΟ Very low
BCI-MI–end-effector robot + tDCS vs BCI-MI–end-effector robot	Not serious	very serious	Serious	Serious	Undetected	15	29	0.91 (−4.43 to 6.24)	⊕ΟΟΟ Very low
BCI-MI–exoskeleton robot vs non–BCI-based interventions	Serious	Not serious	Not serious	Not serious	Undetected	92	94	12.44 (6.42 to 18.46)	⊕⊕⊕Ο Moderate
BCI-MI-FES^l^ vs non–BCI-based interventions	Not serious	Serious	Serious	Not serious	Undetected	304	302	7.75 (4.79 to 10.70)	⊕⊕ΟΟ Low
BCI-MI-FES+ rTMS^m^ vs non–BCI-based interventions	Serious	Not serious	Not serious	very serious	Undetected	20	20	4.00 (−9.78 to 17.78)	⊕ΟΟΟ Very low
BCI-MI–soft robotic glove vs non–BCI-based interventions	Not serious	Serious	Not serious	Not serious	Undetected	52	59	8.38 (3.46 to 13.29)	⊕⊕⊕Ο Moderate
BCI-MI–soft robotic glove vs BCI-MI	Not serious	Serious	Not serious	Serious	Undetected	8	15	1.02 (−5.53 to 7.57)	⊕⊕ΟΟ Low
BCI-MI-TEAS^n^ vs non–BCI-based interventions	Not serious	Serious	Not serious	Serious	Undetected	60	60	13.86 (5.05 to 22.67)	⊕⊕ΟΟ Low

a Risk of bias: downgraded by 1 level if the majority of evidence was derived from studies at high risk of bias.

b Inconsistency: assessed using node-splitting and design-by-treatment interaction models. Evidence was downgraded by 1 level for significant inconsistency (*P*<.05) or substantial heterogeneity (*I*2≥50%) and by 2 levels for *I*2≥80%.

c Indirectness: downgraded by one level if included studies deviated from the predefined population, intervention, comparison, outcome, and study design (PICOS) framework or violated the transitivity assumption.

d Imprecision: downgraded for wide CIs, failure to meet the optimal information size, or estimates failing to meet the minimal clinically important difference. Two levels of downgrading were applied only in cases of very serious imprecision.

e Other considerations: evidence was downgraded by 1 level for evidence of publication bias.

f Intervention: BCI-based combination therapies.

g Comparator: conventional rehabilitation or control.

h MD: mean difference.

i BCI: brain-computer interface.

j MI: motor imagery.

k tDCS: transcranial direct current stimulation.

l FES: functional electric stimulation.

m rTMS: repetitive transcranial magnetic stimulation.

n TEAS: transcutaneous electrical acupoint stimulation.

## Discussion

### Principal Results

This study evaluated the comparative efficacy of 12 distinct BCI-based interventions for poststroke upper limb rehabilitation, involving 2906 patients with stroke across 72 RCTs. Pairwise meta-analysis revealed that, compared with control groups, improvements in FMA-UE and WMFT surpassed the established MCID threshold, whereas those in ARAT and MBI failed to reach this threshold. Although pooled estimates demonstrated robust efficacy, the wide 95% PIs indicate significant clinical variability across diverse settings, underscoring the context-dependent nature of real-world BCI-based interventions. Network meta-analysis revealed that, BCI-MI-TEAS consistently achieved high SUCRA rankings across body functions, body structures, activities, and participation outcomes. Furthermore, BCI-MI–end-effector robot + tDCS showed the largest estimated effect for improving gross and fine motor functions, while BCI-MI–exoskeleton robot achieved a more favorable ranking in enhancing upper limb function, movement quality, and the capacity for independent living in domestic and community settings. Accordingly, clinical decision-making should prioritize tailoring the selection of BCI-based interventions to specific rehabilitation goals. However, with the certainty of evidence for the comparisons between BCI-based interventions and control groups ranging from very low to high, these findings should be interpreted with caution as indicators of potential efficacy rather than as definitive evidence of clinical superiority.

Although the FMA-UE network meta-analysis initially exhibited global and local inconsistency, these issues were mitigated after excluding studies at high risk of bias. Transitivity assessment further substantiated that these variations were primarily driven by disparities in patient baseline characteristics and intervention protocols across nodes. BCI-based interventions leverage neurofeedback mechanisms to facilitate the remodeling of motor circuits [18]. This process necessitates a certain degree of neuroplastic potential and is significantly modulated by clinical variables, particularly the time elapsed since stroke onset [126]. High-frequency training leverages continuous closed-loop feedback to facilitate neural remodeling, whereas short-term interventions often yield suboptimal results due to insufficient practice opportunities [24]. Therefore, the observed inconsistency reflects meaningful clinical heterogeneity rather than methodological bias.

Compared with control groups, BCI-based interventions demonstrated a pooled average improvement in FMA-UE and WMFT that surpassed the established MCID [127,128], a finding that contrasts with prior meta-analyses [21,23,24]. Conversely, pooled improvements in ARAT and MBI fell short of the MCID [56,129], although even these metrics exhibited a greater degree of improvement relative to the findings reported in earlier literature [21,23,24]. This may be attributed to the recent rapid development of BCI technology, including signal acquisition, feature extraction, classification, command translation, and external feedback devices, which has significantly enhanced the efficiency and depth of neural plasticity induction [18,130]. Although these technical advancements have effectively translated neuroplasticity into measurable improvements in body structures and functions, the subsequent transfer to real-world activities and participation remains a more complex process. Whereas the evidence supporting the efficacy of ARAT is graded as high, the confidence in the evidence for other outcomes remains low, and the included studies frequently exhibit a high risk of bias. These findings should be interpreted as demonstrating potential therapeutic gains rather than as establishing definitive clinical superiority.

The wide PIs for FMA-UE, WMFT, ARAT, and MBI demonstrated substantial interindividual variability; individual clinical outcomes following BCI-based interventions remain highly uncertain and context dependent. First, the heterogeneity of patients with stroke regarding their recovery stage, lesion topography, and baseline upper limb impairment imposes stringent demands on the decoding performance and adaptive calibration of BCI systems [131]. Second, BCI-based interventions comprise diverse experimental paradigms, external auxiliary devices, and adjunctive noninvasive brain stimulation; these configurational variations likely modulate the efficacy of limb rehabilitation [18]. Third, the training dosage, including intensity, frequency, and duration, may be insufficient to trigger the neuroplastic changes required to promote effective upper limb recovery in patients with stroke [132]. Finally, the majority of BCI-based interventions are optimized for laboratory tasks rather than for ecological contexts. The absence of realistic, closed-loop designs thus restricts their ability to facilitate meaningful gains in activities and participation [132].

In terms of body structures and functions, BCI-MI-TEAS achieved the highest SUCRA ranking for FMA-UE, suggesting that it may serve as a potentially effective intervention for modulating the physiological substrates of motor recovery post stroke. By applying electrical stimulation to specific acupoints, TEAS acts as a peripheral counterpart to acupuncture, facilitating the restoration of voluntary motor control [133]. This delivers precise sensory afferent feedback that optimizes cortical excitability and reinforces the neural pathways essential for motor recovery [133,134]. Simultaneously, BCI-based interventions promote functional reorganization within the central nervous system, effectively strengthening neuroplasticity across integrated brain regions [57,130]. This bidirectional synchronization between peripheral sensory input and central reorganization optimizes the recovery of impaired neural structures and motor functions. In terms of activities and participation, BCI-MI-TEAS also exerts a positive influence. By matching the distal hand’s peripheral innervation, this approach facilitates the reconstruction of cortical circuits governing fine motor dexterity [135]. By optimizing these physiological structures and fine motor dexterity, this intervention improves overall physical function, effectively translating clinical motor gains into ADL independence and broader social participation within family and community contexts [133]. However, the evidence supporting BCI-MI-TEAS for these outcomes is predominantly graded as low to moderate, with substantial heterogeneity and a high risk of bias across the included studies. These findings should be interpreted with caution, as they are exploratory and hypothesis-generating in nature, representing preliminary signals of efficacy rather than definitive conclusions.

Among the evaluated interventions, BCI-MI-FES demonstrated clinically meaningful improvements in FMA-UE, which is supported by high-certainty evidence, low statistical heterogeneity, and a predominantly moderate risk of bias. Notably, this intervention accounted for the highest number of included studies. It also achieved MCID for improving FMA-UE in patients post stroke, a finding that aligns with previous meta-analyses [21,24]. Although BCI-MI-FES and BCI-MI-TEAS share the same foundation of integrating central neural reorganization with peripheral electrical stimulation, BCI-MI-FES specifically targets motor-driven activation, while BCI-MI-TEAS focuses on neuromodulation [136]. Previous studies have indicated that the synergistic integration of neuromodulation and brain decoding is pivotal for the clinical translation of BCI technology, a paradigm that BCI-MI-TEAS exemplifies [137]. Despite BCI-MI-TEAS currently having a moderate to low evidence quality, the clinical promise of this emerging paradigm is evident, warranting further large-scale, high-quality randomized controlled trials to substantiate its efficacy.

In terms of activities and participation, BCI-MI–end-effector robot + tDCS ranked highest in the current network for ARAT, effectively enhancing grasping, gripping, pinching, and gross motor functions. End-effector robotic training primarily targets distal upper limb control and task-specific practice, which are essential for dexterity and fine motor performance [58,59]. The synergy between BCI-based interventions and tDCS enables precise modulation of neural activity. By modulating cortical excitability, this combination effectively enhances the therapeutic outcomes of BCI-based interventions [19,138]. This is corroborated by the superior SUCRA value of BCI-MI–end-effector robot + tDCS compared to that of BCI-MI–end-effector robot. Concurrently, both interventions yielded significant improvements in functional independence within domestic and community settings. As distal upper limb dexterity is a critical determinant of functional independence, these improvements in motor function facilitate the transition from impairment recovery to enhanced activities and participation [139]. However, given the limited number of studies and the relatively low quality of evidence currently available, these findings should be interpreted with caution and cannot yet be considered as a definitive clinical standard.

Furthermore, BCI-MI–exoskeleton robot demonstrated superior effect size estimates for both WMFT and MBI. By providing real-time feedback aligned with MI-induced intentions, exoskeleton-assisted training enhances multijoint synergy and optimizes range of motion, facilitating the translation of these motor gains into improved independence in domestic and community settings [140,141]. Notably, BCI-MI–exoskeleton robot may be limited by its mechanical complexity, potential discomfort, and challenges in anatomical alignment, which may compromise patient adherence and consequently diminish the therapeutic gains reliant on active neural engagement [142]. However, these findings should be interpreted with caution. While the low statistical heterogeneity lends some confidence to these estimates, the moderate certainty of evidence and high risk of bias underscore the necessity for large-scale, high-quality trials to definitively validate the long-term clinical efficacy of BCI-MI–exoskeleton robot.

### Implications for Practice and Research

Although the overall certainty of the evidence remains limited, BCI-based interventions demonstrate considerable potential for enhancing upper limb rehabilitation in stroke survivors. This study identifies several potential intervention strategies across the domains of body functions, body structures, activities, and participation; however, these findings should be regarded as a foundation for further research rather than as definitive clinical practice guidelines. Notably, BCI-MI-TEAS consistently achieved favorable rankings across multiple outcome domains, marking it as a candidate worthy of further investigation. However, given that the evidence certainty for this intervention remains low to moderate, large-scale, high-quality randomized controlled trials are essential before firm clinical conclusions can be drawn. Furthermore, mean improvements in activities and participation observed in most current BCI-based interventions have yet to reach the MCID. Future research must shift focus beyond mere motor impairment reduction to validate whether these gains translate into meaningful functional independence while incorporating broader social participation–based outcome indicators to assess real-world reintegration.

Blinding in BCI-based interventions is inherently challenging to implement due to the visible nature of EEG caps, interactive interfaces, or external assistive devices, which may compromise trial integrity. The overall quality of evidence for BCI-based interventions is predominantly rated as low to moderate, with wide prediction intervals indicating substantial therapeutic heterogeneity. This evidence base remains fragmented across trials regarding stroke chronicity, baseline impairment severity, intervention protocols, and outcome measures. Moreover, as most RCTs are small-scale with highly diverse control groups, node sparsity may limit the stability of our rankings and the precision of indirect comparisons. The ranking of these interventions must be viewed with caution, as these estimates may reflect underlying imbalances in patient characteristics or intervention intensity rather than purely comparative therapeutic efficacy. Therefore, these findings should be interpreted as providing a clinical direction for future practice rather than serving as definitive clinical recommendations for all patients with stroke.

### Limitations

Several limitations should be considered. First, with only 8 trials (11.1%) at low risk of bias and substantial heterogeneity, the robustness of pairwise effect estimates is limited, along with a predominantly low to moderate certainty of evidence for intervention comparisons, which warrants cautious interpretation of the network meta-analysis findings. Second, several intervention nodes were informed by a limited number of studies, reducing the precision of some comparisons. Third, decomposition of BCI-based interventions into specific experimental paradigms, external auxiliary devices, and noninvasive brain stimulation resulted in overly fragmented network nodes, thereby diluting comparison sample sizes and reducing overall statistical power. Fourth, the paucity of outcome measures addressing the participation domain limits this study’s ability to fully evaluate the potential of BCI-based interventions in facilitating social reintegration. Finally, many comparisons relied primarily on indirect evidence because direct head-to-head trials between BCI-based interventions remain scarce.

### Conclusion

To the best of our knowledge, this study represents the first network meta-analysis to evaluate the comparative efficacy of various BCI experimental paradigms combined with different external auxiliary devices and adjunctive noninvasive brain stimulation for poststroke upper limb rehabilitation. Previous reviews have often overlooked the nuances of BCI experimental paradigms and noninvasive brain stimulation; furthermore, they have been unable to provide comparative assessments of therapeutic efficacy [21,23,24]. This review suggests that BCI-based interventions may contribute significantly to poststroke upper limb rehabilitation. To optimize therapeutic outcomes, interventions should be tailored to individual patient needs and specific rehabilitation goals. Notably, BCI-MI-TEAS demonstrated consistently favorable rankings across body functions, body structures, activities, and participation outcomes, while BCI-MI–end-effector robot + tDCS and BCI-MI–exoskeleton robot demonstrated promising rankings regarding activities and participation. However, substantial heterogeneity, significant risk of bias, and relatively low certainty in the evidence necessitate a cautious interpretation of these findings. These observations should be viewed as exploratory and hypothesis-generating rather than as definitive clinical recommendations. Future research should prioritize validating these promising strategies through large-scale, high-quality trials to establish a robust scientific foundation.

## Supplementary material

10.2196/92940 Multimedia Appendix 1 Tables depicting the characteristics of brain-computer interface (BCI)–based intervention categories, characteristics of included studies, assessment of the transitivity assumption, results of meta-regression and node-splitting analysis, assessment of publication bias for pairwise meta-analyses, assessment of publication bias and small-study effects for network meta-analysis, results of node-splitting analysis after excluding high-risk studies, and league table of BCI-based interventions after excluding high-risk studies as well as figures depicting forest plots of pairwise meta-analysis, subgroup forest plots of pairwise meta-analysis, forest plot of subgroup analyses, ranking probability, funnel plot for pairwise meta-analysis to detect publication bias, comparison-adjusted funnel plot for network meta-analysis, leave-one-out sensitivity analysis plot, and ranking probability after excluding high-risk studies.

10.2196/92940 Multimedia Appendix 2 Search strategy.

10.2196/92940 Checklist 1 PRISMA (Preferred Reporting Items for Systematic Reviews and Meta-Analyses) 2020 checklist.

10.2196/92940 Checklist 2 PRISMA-S (Preferred Reporting Items for Systematic Reviews and Meta-Analyses literature search extension) checklist.

## Abbreviations

ADL: activities of daily living
ARAT: Action Research Arm Test
BCI: brain-computer interface
CNKI: China National Knowledge Infrastructure
EEG: electroencephalography
ERP: P300 Event-Related Potentials
FES: functional electrical stimulation
FMA-UE: Fugl-Meyer Assessment-Upper Extremity
GRADE: Grading of Recommendations, Assessment, Development, and Evaluation
ICF: International Classification of Functioning, Disability and Health
MBI: Modified Barthel Index
MCID: minimal clinically important difference
MD: mean difference
MI: motor imagery
NMA: network meta-analysis
PI: prediction interval
PRISMA: Preferred Reporting Items for Systematic Reviews and Meta-Analyses
PRISMA-S: Preferred Reporting Items for Systematic Reviews and Meta-Analyses literature search extension
RCT: randomized controlled trial
RoB 2: Cochrane Risk of Bias 2 tool
rTMS: repetitive transcranial magnetic stimulation
SSVEP: steady-state visual evoked potentials
tDCS: transcranial direct current stimulation
TEAS: transcutaneous electrical acupoint stimulation
WMFT: Wolf Motor Function Test
