# Supplementary material for: Comparative Effectiveness of Noninvasive Brain-Computer Interface–Based Interventions for Upper Limb Rehabilitation in Poststroke Hemiplegia: Systematic Review and Network Meta-Analysis of Randomized Controlled Trials
Source: J Med Internet Res. 2026 Aug 28;28:e92940. doi: 10.2196/92940 (PMC13524221; doi:10.2196/92940)
Supplement: Supplementary file 2 [file jmir-v28-e92940-s004.pdf]

PRISMA-S Checklist

Section/topic	#	Checklist item	Location(s) Reported
**INFORMATION SOURCES AND METHODS**			
Database name	1	Name each individual database searched, stating the platform for each.	Page 8, Line 179-182
Multi-database searching	2	If databases were searched simultaneously on a single platform, state the name of the platform, listing all of the databases searched.	Page 8, Line 179-182
Study registries	3	List any study registries searched.	Page 8, Line 179-182
Online resources and browsing	4	Describe any online or print source purposefully searched or browsed (e.g., tables of contents, print conference proceedings, web sites), and how this was done.	Page 8, Line 179-182
Citation searching	5	Indicate whether cited references or citing references were examined, and describe any methods used for locating cited/citing references (e.g., browsing reference lists, using a citation index, setting up email alerts for references citing included studies).	Page 8, Line 182-190
Contacts	6	Indicate whether additional studies or data were sought by contacting authors, experts, manufacturers, or others.	Page 8, Line 199-201
Other methods	7	Describe any additional information sources or search methods used.	NA
**SEARCH STRATEGIES**			
Full search strategies	8	Include the search strategies for each database and information source, copied and pasted exactly as run.	Multimedia Appendix 1
Limits and restrictions	9	Specify that no limits were used, or describe any limits or restrictions applied to a search (e.g., date or time period, language, study design) and provide justification for their use.	Page 8, Line 174-175;
Search filters	10	Indicate whether published search filters were used (as originally designed or modified), and if so, cite the filter(s) used.	NA
Prior work	11	Indicate when search strategies from other literature reviews were adapted or reused for a substantive part or all of the search, citing the previous review(s).	NA

Updates	12	Report the methods used to update the search(es) (e.g., rerunning searches, email alerts).	NA
Dates of searches	13	For each search strategy, provide the date when the last search occurred.	Page 8, Line 182
**PEER REVIEW**			
Peer review	14	Describe any search peer review process.	NA
**MANAGING RECORDS**			
Total Records	15	Document the total number of records identified from each database and other information sources.	Page 33, Figure 1
Deduplication	16	Describe the processes and any software used to deduplicate records from multiple database searches and other information sources.	Page 8, Line 192-193

PRISMA-S: An Extension to the PRISMA Statement for Reporting Literature Searches in Systematic Reviews
Rethlefsen ML, Kirtley S, Waffenschmidt S, Ayala AP, Moher D, Page MJ, Koffel JB, PRISMA-S Group.
Last updated February 27, 2020.
