# Supplementary material for: Comparative Effectiveness of Noninvasive Brain-Computer Interface–Based Interventions for Upper Limb Rehabilitation in Poststroke Hemiplegia: Systematic Review and Network Meta-Analysis of Randomized Controlled Trials
Source: J Med Internet Res. 2026 Aug 28;28:e92940. doi: 10.2196/92940 (PMC13524221; doi:10.2196/92940)
Supplement: Supplementary file 3 [file jmir-v28-e92940-s001.docx]

**Table of content**

[Table S1. BCI-based intervention categories characteristics.	1](#_Toc21499)

[Table S2. Characteristics of the included studies.	4](#_Toc19602)

[Table S3. Assessment of the transitivity assumption across treatment nodes for the outcomes.	12](#_Toc4437)

[Figure S1. Forest plots of pairwise meta-analysis for BCI-based intervention versus control groups.	19](#_Toc7012)

[Figure S2. Subgroup forest plots of pairwise meta-analysis stratified by BCI-based intervention versus control groups.	22](#_Toc2145)

[Figure S3. Forest plot of subgroup analyses.	26](#_Toc9053)

[Table S4. Meta-regression (n>10).	28](#_Toc25156)

[Table S5. Results of node-splitting analysis for evaluating local inconsistency.	29](#_Toc5357)

[Figure S4. Ranking probability.	30](#_Toc4613)

[Figure S5. Funnel plot for pairwise meta-analysis to detect publication bias.	31](#_Toc29173)

[Figure S6. Comparison-adjusted funnel plot for network meta-analysis to detect publication bias.	33](#_Toc18734)

[Table S6. Assessment of publication bias for pairwise meta-analyses.	35](#_Toc10327)

[Table S7. Assessment of publication bias and small-study effects for network meta-analysis.	36](#_Toc16019)

[Figure S7. Leave-one-out sensitivity analysis plot.	37](#_Toc31270)

[Table S8. Results of node-splitting analysis for evaluating local inconsistency after excluding high-risk studies.	41](#_Toc16450)

[Figure S8. Ranking probability after excluding high-risk studies.	42](#_Toc26721)

[Table S9. League table showing mean differences and their 95% CIs among BCI-based interventions after excluding high-risk studies.	43](#_Toc4574)

**Table S1.** BCI-based intervention categories characteristics.

| **Treatment category** | **Description** |
| --- | --- |
| BCI-Event-related potentials- Functional electric stimulation  (BCI-ERP-FES) | The BCI system utilizes P300 to detect the patient's specific target attention. Once the neural response meets the predefined threshold, the BCI triggers. Functional electrical stimulation to activate the targeted upper limb muscles, thereby executing the patient's intended movement and facilitating closed-loop sensorimotor feedback [1]. |
| BCI-Motor imagery  (BCI-MI) | Patients perform MI to modulate neural activity. The BCI provides visual neurofeedback (e.g., a virtual hand or dynamic bar) as a functional reward for successful mental practice. This closed-loop system reinforces neural pathways through purely informational feedback, without the involvement of external physical assistance [2-4]. |
| BCI-Motor imagery-End-effector robot  (BCI-MI-End-effector robot) | Patients perform sustained MI to modulate neural activity. Once the BCI decodes the volitional intent, it triggers an end-effector robot to provide contingent mechanical assistance, translating the user’s imagined movement into synchronous physical execution [5]. |
| BCI-Motor imagery-End-effector robot + transcranial direct current stimulation  (BCI-MI-End-effector robot + tDCS) | tDCS is applied to prime cortical excitability by modulating resting membrane potentials. Subsequently, patients perform sustained MI to modulate neural activity. Once the BCI decodes the volitional intent, it triggers an end-effector robot to provide contingent mechanical assistance, translating the user’s imagined movement into synchronous physical execution [6]. |
| BCI-Motor imagery-Exoskeleton robot  (BCI-MI-Exoskeleton robot) | BCI monitors neural patterns during sustained MI to decode the user's volitional intent. Upon successful detection, a wearable exoskeleton robot is synchronously triggered to provide multi-joint physical assistance, translating the imagined movement into therapeutic execution [7]. |
| BCI-Motor imagery-Exoskeleton robot + transcranial direct current stimulation  (BCI-MI-Exoskeleton robot + tDCS) | tDCS is applied to prime cortical excitability by modulating resting membrane potentials. Subsequently, BCI monitors neural patterns during sustained MI to decode the user's volitional intent. Upon successful detection, a wearable exoskeleton robot is synchronously triggered to provide multi-joint physical assistance, translating the imagined movement into therapeutic execution [8]. |
| BCI-Motor imagery-Functional electric stimulation  (BCI-MI-FES) | BCI monitors neural patterns during motor imagery. Upon successful detection of the imagery intent, the system synchronously triggers FES to activate the paretic muscles [9]. |
| BCI-Motor imagery-Functional electric stimulation + Repetitive Transcranial magnetic stimulation  (BCI-MI-FES + rTMS) | rTMS is initially applied to modulate cortical excitability and prime the motor network. Patients then perform MI to generate sensorimotor rhythms captured by the BCI. Upon intent detection, the system synchronously triggers FES to execute muscle contractions [10]. |
| BCI-Motor imagery-Soft robotic gloves  (BCI-MI-Soft robotic glove) | BCI monitors neural patterns during sustained MI to decode the user's volitional intent. Upon successful detection, a wearable soft robotic robot is synchronously triggered to provide multi-joint physical assistance, translating the imagined movement into therapeutic execution [11]. |
| BCI-Motor imagery-Transcutaneous electrical acupoint stimulation  (BCI-MI-TEAS) | Patients perform sustained MI to modulate neural activity. Upon real-time decoding of the volitional intent, the BCI triggers transcutaneous electrical acupoint stimulation (TEAS) at specific acupoints—typically LI4 (Hegu), TE5 (Waiguan), PC6 (Neiguan), and LI11 (Quchi)—to provide contingent somatosensory feedback. This targeted stimulation enhances afferent input to the motor cortex, reinforcing the associative link between mental intent and peripheral sensation [12]. |
| BCI-Steady-state visual evoked potentials-Exoskeleton robot  (BCI-SSVEP-Exoskeleton robot) | BCI integrates a brain-computer interface driven by steady-state visual evoked potentials with exoskeleton robot training. Patients evoke distinct neural oscillations by focusing on flickering visual stimuli at specific frequencies to signal their motor intention. Upon decoding this intent, the system triggers the motorized exoskeleton to physically guide and assist the patient's impaired upper limb in performing structured therapeutic movements [13]. |
| BCI-Steady-state visual evoked potentials-Soft robotic gloves  (BCI-SSVEP-Soft robotic glove) | BCI identifies neural oscillations in the visual cortex triggered by SSVEP (e.g., flickering targets on a screen). Upon detecting the specific frequency corresponding to the user's focus, the system synchronously activates a soft robotic glove to execute multi-finger movements [14]. |

**Table S2.** Characteristics of the included studies.

| Author, year  (Country) | Sample size,  n (male/female) | Age, years  (EG/CG) | Baseline FMA-UE scores  (EG/CG) | Time from stroke onset (months)  (EG/CG) | Experimental interventions | | Control  group | BCI training intensity | Risk of bias | Outcome measures |
| --- | --- | --- | --- | --- | --- | --- | --- | --- | --- | --- |
|  |  |  |  |  | Type of primary multi-sensory feedback | Intervention protocol |  |  |  |  |
| Ramirez-Nava et al, 2023 [1]  (Thailand) | EG: 7(5/2)  CG: 7(6/1) | 51.80 ± 20.30 / 54.10 ± 21.40 | 26.10 ±11.50 / 10.50 ± 5.20 | 51.33 ± 60.13 / 58.00 ± 47.47 | Real-Human Imitation-Based Visual Feedback | BCI-ERP-FES | FES | 3d/w, 4w | high | FMA-UE / ARAT |
| He et al, 2025 [15]  (China) | EG: 25(20/5)  CG: 23(14/7) | 58.60 ± 12.80 /  58.30 ± 12.80 | 25.75 ± 5.83 / 19.00 ± 3.00 | 1.11 ± 0.62 /  1.39 ± 0.64 | Screen-based virtual visual feedback + Auditory feedback | BCI-MI | CRT | 20min/d, 5d/w, 2w | low | FMA-UE |
| He et al, 2025 [16]  (China) | EG: 18(13/5)  CG: 18(12/6) | 56.70 ±9.30 / 52.40 ±8.60 | 9.10 ± 2.00 /  9.70 ± 2.60 | 0.87 ± 0.17 /  0.87 ± 0.42 | Screen-based virtual visual feedback + Auditory feedback | BCI-MI | CRT | 30min/d, 6d/w, 2w | moderate | FMA-UE /MBI |
| Hu et al, 2021 [3]  (China) | EG: 7(4/3)  CG: 5(4/1) | 44.90 ± 7.50 /  60.40 ± 16.80 | 12.70 ± 8.88 / 13.80 ± 6.65 | 7.90 ± 6.50 /  7.30 ± 4.50 | Screen-based virtual visual feedback | BCI-MI | MIT | 30min/d | moderate | FMA-UE / ARAT / MBI |
| Lu et al, 2025 [17]  (China) | EG: 21(10/11)  CG: 19(16/3) | 62.17 ± 5.27 /  61.34 ± 5.36 | 25.87 ± 3.25 / 26.15 ± 3.13 | 4.21 ± 1.19 /  4.12 ± 1.11 | NA | BCI-MI | CRT | 20min/d, 7d/w, 8w | high | FMA-UE / MBI |
| Pichiorri et al, 2015 [2]  (Rome) | EG: 14  CG: 14 | 64.10 ± 8.40 /  59.60 ± 12.70 | 23.40 ± 17.30 / 24.20 ± 18.20 | 2.70 ± 1.70 /  2.50 ± 1.20 | Real-Human Imitation-Based Visual Feedback | BCI-MI | MIT | 60min/d, 3d/w, 4w | low | FMA-UE |
| Mihara et al, 2013 [4]  (Japan) | EG: 10(8/2)  CG: 10(4/6) | 56.40 ±7.51 /  60.50 ± 8.04 | 20.70 ± 13.80/  24.00 ± 13.80 | 4.82 ±1.09 /  4.05 ±1.19 | Screen-based virtual visual feedback | BCI-MI | MIT | 20min/d, 3d/w, 2w | moderate | FMA-UE / ARAT |
| Ang et al, 2014 [18]  (Singapore) | EG: 6 (4/2)  CG: 8 (5/3) | 54.00 ± 8.90 /  51.10 ± 6.30 | 33.00 ± 16.20 / 25.50 ± 11.50 | 9.52 ± 2.13 /  13.27 ± 5.03 | Screen-based visual abstract feedback | BCI-MI-End-effector robot | End-effector robot | 60min/d, 3d/w, 6w | moderate | FMA-UE |
| Ang et al, 2015 [19]  (Singapore) | EG: 11(9/2)  CG: 15(7/8) | 48.50 ± 13.50 /  53.60 ± 9.50 | 26.30 ± 10.30 / 26.60 ± 18.90 | 12.77 ± 9.69 /  7.82 ± 6.13 | Screen-based visual abstract feedback | BCI-MI-End-effector robot | End-effector robot | 90min/d, 3d/w, 4w | moderate | FMA-UE |
| Chen et al, 2026 [20]  (China) | EG: 30(24/6)  CG: 30(22/8) | 58.03 ± 7.19 / 60.53 ± 6.79 | 19.77 ± 4.97 / 19.13 ± 5.79 | 2.21 ± 0.27 /  2.03 ± 0.19 | Screen-based virtual visual feedback + Auditory feedback | BCI-MI-End-effector robot | CRT | 20min/d, 5d/w, 4w | high | FMA-UE / WMFT / MBI |
| Foong, 2020 [21]  (Singapore) | EG: 6(4/2)  CG: 7(4/3) | 54.00±8.90 /  58.00±19.30 | 33.70 ± 12.20 / 23.40 ± 14.00 | NA | Screen-based virtual visual feedback | BCI-MI-End-effector robot | End-effector robot | 60min/d, 3d/w, 6w | high | FMA-UE |
| Huang, 2024 [5]  (China) | EG: 11  CG: 11 | 55.09 ± 11.42 /  54.91 ± 6.55 | 33.00 ± 11.56 / 31.00 ± 12.15 | 4.91 ± 0.83 /  4.73 ± 1.85 | Screen-based virtual visual feedback + Auditory feedback | BCI-MI-End-effector robot | CRT | 60min/d, 4d/w, 2w | high | FMA-UE / ARAT / MBI |
| Lin et al, 2018 [22]  (China) | EG: 5(3/2)  CG: 5(5/0) | 52.20 ± 7.70 /  56.20 ± 7.70 | 36.00 ± 9.60 / 36.80 ± 10.30 | 17.80 ± 15.30 /  18.80 ± 13.00 | NA | BCI-MI-End-effector robot | CRT | 35min/d, 3d/w, 4w | moderate | FMA-UE |
| Lu et al, 2025 [23]  (China) | EG: 20(17/3)  CG: 19(9/10) | 52.60 ± 14.48 /  50.42 ± 13.61 | 13.90 ± 6.97 / 13.68 ± 6.28 | 12.00 ± 6.90 /  9.21 ± 7.20 | Screen-based visual abstract feedback | BCI-MI-End-effector robot | MIT | 30min/d, 5d/w, 4w | moderate | FMA-UE |
| Chew et al, 2020 [8]  (Singapore) | EG: 10(6/4)  CG: 9(8/1) | 52.20 ± 11.80 / 56.40 ± 9.60 | 35.30 ± 7.80 / 32.60 ± 8.10 | 31.30 ± 24.50 / 33.30 ± 15.10 | Screen-based visual abstract feedback | BCI-MI-End-effector robot + tDCS | BCI-MI-End-effector robot | 4w | low | FMA-UE |
| Gao et al, 2024 [6]  (China) | EG: 15(11/4)  CG1: 15(12/3)  CG2: 15(12/3) | 55.73 ± 8.60 /  54.60 ± 8.23 /  53.20±13.55 | 20.67 ± 9.13 / 20.93 ± 10.74 /  19.53 ± 11.50 | 1.95 ± 1.03 /  2.34 ± 1.55 /  2.11 ± 1.58 | Screen-based virtual visual feedback + Auditory feedback | BCI-MI-End-effector robot + tDCS | CG1: tDCS  CG2: BCI-MI-End-effector robot | 20min/d, 5d/w, 4w | moderate | FMA-UE / ARAT / MBI |
| Ming et al, 2025 [24]  (China) | EG: 14(10/4)  CG1: 14(9/5)  CG2: 15(8/7) | 54.93 ± 8.73 /  60.79 ± 11.48 / 56.13±8.63 | 22.57 ± 3.36 / 22.29 ± 1.75 /  22.00 ± 0.49 | 0.66 ± 0.12 /  0.73 ± 0.21 /  1.45 ± 0.74 | Screen-based visual abstract feedback | BCI-MI-End-effector robot + tDCS | CG1: CRT  CG2: BCI-MI-End-effector robot | 30min/d, 5d/w, 4w | moderate | FMA-UE /ARAT / MBI |
| Cantillo-Negrete et al, 2021 [7]  (Mexico) | EG: 5(2/3)  CG: 5(3/2) | 59.80 ± 11.20 /  60.00 ±16.70 | 17.50 ±15.30 / 23.10 ±16.10 | 6.37 ± 2.74 /  2.98 ± 1.39 | Screen-based visual abstract feedback | BCI-MI-Exoskeleton robot | Exoskeleton robot | 3d/w, 3w | high | FMA-UE / ARAT |
| Cantillo-Negrete et al, 2025 [25]  (Mexico) | EG: 10(6/4)  CG: 9(8/1) | 47.80 ± 15.70 /  55.80 ± 15.00 | 31.33 ± 12.79 / 30.13 ± 17.05 | 12.70 ± 5.80 /  14.70 ± 12.40 | Screen-based visual abstract feedback | BCI-MI-Exoskeleton robot | Exoskeleton robot | 30min/d, 5d/w, 6w | moderate | FMA-UE / ARAT |
| Frolov et al, 2017 [26]  (Russia) | EG:55(34/21)  CG:19(14/5) | 57.00 ± 12.59 / 59.00 ± 11.11 | 20.85 ± 23.17 / 32.23 ± 23.00 | 8.33 ± 6.67 /  7.33 ± 8.89 | Screen-based visual abstract feedback | BCI-MI-Exoskeleton robot | Exoskeleton robot | 30min/d, 5d/w, 2w | moderate | FMA-UE / ARAT |
| Fu, 2022 [27]  (China) | EG: 6(5/1)  CG: 6(5/1) | 57.00 ± 13.50 /  61.50 ± 11.50 | 13.67 ± 11.48 / 12.83 ± 9.26 | 5.49 ± 6.07 /  2.62 ± 1.89 | Auditory feedback | BCI-MI-Exoskeleton robot | Exoskeleton robot | 30min/d, 5d/w, 4w | moderate | FMA-UE / ARAT / MBI |
| Fu, 2023 [28]  (China) | EG: 30(23/7)  CG: 31(25/6) | 55.93 ± 11.05 /  59.00 ± 14.49 | NA | 3.44 ± 3.44 /  3.08 ± 2.75 | Auditory feedback | BCI-MI-Exoskeleton robot | Exoskeleton robot | 30min/d, 5d/w, 4w | high | FMA-UE |
| Li et al, 2022 [29]  (China) | EG: 12(12/0)  CG: 12(12/0) | 43.80 ± 14.70 /  55.00 ± 12.20 | 22.58 ± 13.71 / 24.25 ± 17.08 | NA | Screen-based visual abstract feedback + Auditory feedback | BCI-MI-Exoskeleton robot | CRT | 60min/d, 5d/w, 2w | high | FMA-UE / WMFT / MBI |
| Liang et al, 2020 [30]  (China) | EG: 15(12/3)  CG: 15(9/6) | 50.60 ± 13.46 /  57.94 ± 8.84 | 16.27 ± 10.27 / 19.33 ± 12.37 | 3.00 ± 2.98 /  2.93 ± 1.44 | Screen-based virtual visual feedback | BCI-MI-Exoskeleton robot | Exoskeleton robot | 30min/d, 5d/w, 4w | high | FMA-UE / MBI |
| Liu et al, 2023 [31]  (China) | EG: 19(15/4)  CG: 18(10/8) | 51.26 ± 11.06 /  52.89 ± 13.07 | 25.37 ± 9.27 / 23.94 ± 6.95 | 0.61 ± 0.56 /  0.59 ± 0.61 | Screen-based visual abstract feedback + Auditory feedback | BCI-MI-Exoskeleton robot | Exoskeleton robot | 30min/d, 5d/w, 4w | moderate | FMA-UE / MBI |
| Liu, 2024 [32]  (China) | EG: 20(12/8)  CG: 20(13/7) | 54.82 ± 7.62 / 56.55 ± 9.99 | NA | 4.41 ± 2.29 /  3.75 ± 2.19 | Screen-based virtual visual feedback | BCI-MI-Exoskeleton robot | CRT | 4w | high | FMA-UE |
| Liu et al, 2024 [33]  (China) | EG: 25(18/7)  CG: 28(20/8) | 57.64 ± 13.12 /  52.68 ± 10.01 | 27.44 ± 12.71 / 27.52 ± 13.48 | 4.44 ± 2.29 /  4.40 ± 3.93 | Screen-based virtual visual feedback | BCI-MI-Exoskeleton robot | Exoskeleton robot | 30min/d, 5d/w, 4w | high | FMA-UE / WMFT / MBI |
| Lyukmaov, 2018[34]  (Russia) | EG: 35(24/11)  CG: 20(15/5) | 52.00 ± 5.25 / 56.86 ± 3.35 | NA | 6.00 ± 2.15 /  6.31 ± 3.08 | NA | BCI-MI-Exoskeleton robot | Exoskeleton robot | 40min/d, 6d/w, 2w | moderate | FMA-UE / ARAT |
| Ramos-Murguialday et al, 2019 [35]  (Germany) | EG: 16(9/7)  CG: 12(7/5) | 49.30 ± 12.50 /  50.30 ± 12.20 | 11.16 ± 1.73 / 13.29 ± 2.86 | NA | NA | BCI-MI-Exoskeleton robot | Exoskeleton robot | 60min/d, 5d/w, 4w | moderate | FMA-UE |
| Várkuti, 2013 [36]  (Germany) | EG: 6(5/1)  CG: 3(2/1) | 40.94 ± 14.50 / 50.67 ± 6.66 | 34.99 ± 11.84 /  47.00 ± 13.90 | 6.80 ± 6.50 /  11.67 ± 13.51 | Screen-based virtual visual feedback | BCI-MI-Exoskeleton robot | Exoskeleton robot | 60min/d, 3d/w, 4w | high | FMA-UE |
| Wu et al, 2020 [37]  (China) | EG: 14(9/5)  CG: 11(9/2) | 62.93 ± 10.56 /  64.82 ± 7.22 | 18.43 ± 2.65 /  14.09 ± 2.51 | 2.11 ± 0.30 /  2.17 ± 1.27 | Screen-based visual abstract feedback | BCI-MI-Exoskeleton robot | CRT | 60min/d, 5d/w, 4w | moderate | FMA-UE / ARAT / WMFT |
| Zanona et al, 2022 [38]  (Brazil) | EG: 23(12/11)  CG: 21(11/10) | 62.20 ± 9.80 /  61.00 ± 3.00 | 32.10 ±16.80 /  37.00 ± 13.50 | 3.20 ± 1.15 /  2.87 ± 1.54 | Real-Human Imitation-Based Visual Feedback | BCI-MI-Exoskeleton robot | CRT | 30min/, 5d/w, 2w | low | FMA-UE |
| Zhang et al, 2025 [39]  (China) | EG: 15(10/5)  CG: 15(12/3) | 55.27 ± 9.93 / 62.60 ± 10.99 | 35.87 ± 13.80 / 35.40 ± 14.03 | 0.41 ± 0.14 /  0.40 ± 0.11 | Screen-based virtual visual feedback + Auditory feedback | BCI-MI-Exoskeleton robot | CRT | 30min/d, 5d/w, 4w | high | FMA-UE / MBI |
| Zheng et al, 2023 [40]  (China) | EG: 40(26/14)  CG: 41(29/12) | 56.61 ± 11.33 / 56.13 ± 13.53 | NA | 2.14 ± 2.88 /  3.24 ± 3.29 | NA | BCI-MI-Exoskeleton robot | CRT | 45min/d, 6d/w, 4w | high | WMFT / ARAT |
| Zhou et al, 2024 [41]  (China) | EG: 25(17/8)  CG: 25(15/10) | 57.12 ± 5.54 /  58.52 ± 4.86 | 26.12 ± 11.67 / 26.60 ± 8.84 | NA | Screen-based visual abstract feedback + Auditory feedback | BCI-MI-Exoskeleton robot | CRT | 40min/d, 5d/w, 4w | low | FMA-UE/ ARAT /WMFT |
| Wang, 2023 [42]  (China) | EG: 15(9/6)  CG: 13(8/7) | 62.80 ± 11.16 /  62.67 ± 10.70 | 12.07 ± 2.09 / 11.80 ± 1.66 | 0.45 ± 0.07 /  0.42 ± 0.09 | Screen-based visual abstract feedback | BCI-MI-Exoskeleton robot + tDCS | tDCS | 45min/d, 5d/w, 4w | moderate | FMA-UE / ARAT |
| Biasiucci et al, 2018 [9]  (Switzerland) | EG: 14  CG: 13 | NA | 21.16 ±10.18 / 19.90 ±11.20 | 39.80 ± 45.90 /  33.50 ± 30.50 | Screen-based visual abstract feedback | BCI-MI-FES | FES | 60min/d, 2d/w, 5w | moderate | FMA-UE |
| Chen et al, 2023 [43]  (China) | EG: 23(11/12)  CG: 23(16/7) | 63.68 ± 11.03 /  61.38 ± 10.38 | 27.82 ± 13.33 / 30.22 ± 15.22 | 0.67 ± 0.12 /  0.74 ± 0.21 | Screen-based virtual visual feedback + Auditory feedback | BCI-MI-FES | FES | 40min/d, 5d/w, 4w | high | FMA-UE / ARAT |
| Dun et al, 2023 [44]  (China) | EG: 12(8/4)  CG: 10(6/4) | 53.47 ± 4.23 /  56.19 ± 9.86 | 27.43 ± 16.98 / 27.03 ± 15.19 | 1.70 ± 0.40 /  1.90 ± 0.50 | Real-human imitation-based visual feedback | BCI-MI-EFS | CRT | 8w | high | FMA-UE / MBI |
| Gao et al, 2025 [45]  (China) | EG: 20(17/3)  CG: 20(18/2) | 52.55 ± 13.38 /  55.16 ± 15.99 | 27.35 ± 7.10 / 26.75 ± 6.03 | 5.63 ± 3.70 /  4.90 ± 3.97 | Auditory feedback | BCI-MI-FES | FES | 20min/d, 5d/w, 4w | moderate | FMA-UE / MBI |
| Hou et al, 2024 [46]  (China) | EG: 29(13/16)  CG: 28(15/13) | 68.93 ± 5.20 /  67.74 ± 4.60 | 15.93 ± 4.14 / 17.04 ± 4.39 | 1.41 ± 0.79 /  1.56 ± 0.76 | Screen-based virtual visual feedback + Auditory feedback | BCI-MI-FES | FES | 20min/d, 5d/w, 4w | high | FMA-UE / WMFT |
| Kim et al, 2016 [47]  (Korea) | EG: 15(6/9)  CG: 15(6/9) | 59.07 ± 8.07 / 59.93 ± 9.79 | 26.80 ± 7.22 / 21.87 ± 8.22 | 8.27 ± 1.98 /  7.80 ± 1.78 | Real-human imitation-based visual feedback | BCI-MI-FES | CRT | 30min/d, 5d/w, 5w | moderate | FMA-UE /MBI |
| Kim et al, 2025 [48]  (Korea) | EG: 12(10/2)  CG: 13(9/4) | 49.00 ± 16.90 / 46.00 ± 12.80 | 29.92 ± 11.95 / 33.54 ± 11.10 | 18.2 ± 16.2 / 22.2 ± 23.4 | Screen-based visual abstract feedback | BCI-MI-FES | FES | 60min/d, 5d/w, 5w | low | FMA-UE |
| Krueger et al, 2024 [49]  (Germany) | EG: 6  CG: 6 | NA | 11.30 ± 4.60 / 10.00 ± 3.60 | NA | NA | BCI-MI-FES | FES | / | moderate | FMA-UE |
| Lee et al, 2022 [50]  (Korea) | EG: 13(4/9)  CG: 13(6/7) | 55.15 ± 11.57 /  58.30 ± 9.19 | 30.53 ± 5.05 / 29.52 ± 3.64 | 7.46 ± 1.61 /  8.30 ± 1.97 | Screen-based virtual visual feedback | BCI-MI-EFS | FES | 30min/d, 5d/w, 4w | moderate | FMA-UE / WMFT / MBI |
| Liang et al, 2024 [51]  (China) | EG: 32(21/11)  CG: 32(19/13) | 57.06 ± 6.04 /  56.41 ± 4.98 | 19.10 ± 5.02 / 18.95 ± 4.91 | 0.78 ± 0.24 /  0.67 ± 0.23 | Screen-based virtual visual feedback + Auditory feedback | BCI-MI-FES | FES | 20min/d, 6d/w, 4w | moderate | FMA-UE / MBI |
| Liao et al, 2023[52]  (China) | EG: 20(8/12)  CG: 20(9/11) | 61.50 ± 3.80 /  61.00 ± 3.70 | 18.50 ± 6.46 / 18.70 ± 6.23 | 0.66 ± 0.07 /  0.68 ± 0.08 | Screen-based virtual visual feedback | BCI-MI-FES | CRT | 45-60min/d, 5d/w, 3w | moderate | FMA-UE |
| Liu et al, 2023 [53]  (China) | EG: 30(22/8)  CG: 30(19/11) | 52.27 ± 11.13 /  50.33 ± 16.35 | 37.52 ± 16.93 / 40.29 ± 15.93 | 0.60 ± 0.31 /  0.56 ± 0.34 | Screen-based virtual visual feedback + Auditory feedback | BCI-MI-FES | CRT | 20min/d, 5d/w, 3w | low | FMA-UE / ARAT / MBI |
| Liu, 2023 [54]  (China) | EG: 30(22/8)  CG: 30(19/11) | 51.70 ± 9.80 /  50.00 ± 10.70 | 29.94 ± 15.19 / 35.28 ± 14.29 | 0.60 ± 0.26 /  0.56 ± 0.22 | Screen-based virtual visual feedback | BCI-MI-FES | FES | 20min/d, 5d/w, 3w | moderate | FMA-UE / WMFT / MBI |
| Ma et al, 2021 [55]  (China) | EG: 20(16/4)  CG: 20(15/5) | 50.90 ± 12.64 /  58.30 ± 11.23 | 20.80 ± 11.21 / 20.75 ± 10.77 | 5.90 ± 2.99 /  6.45 ± 3.38 | Screen-based visual abstract feedback + Auditory feedback | BCI-MI-FES | CRT | 10d | low | FMA-UE / ARAT |
| Miao et al, 2020 [56]  (Austria) | EG: 8(6/2)  CG: 8(6/2) | 48.75 ± 18.06 /  50.25 ± 18.89 | 19.50 ± 9.90 / 20.60 ± 11.40 | 18.25 ± 11.23 /  11.13 ± 5.92 | Screen-based visual abstract feedback + Auditory feedback | BCI-MI-FES | CRT | 20min/d, 3d/w, 4w | moderate | FMA-UE |
| Rungsirisilp et al, 2023 [57]  (Thailand) | EG: 9(7/2)  CG: 8(7/1) | 61.11 ± 7.16 / 61.63 ± 7.83 | 22.78 ± 13.58 / 29.38 ± 8.45 | 51.33 ± 60.13 / 58.00 ± 47.47 | Real-Human Imitation-Based Visual Feedback | BCI-MI-FES | FES | 3d/w, 4w | moderate | FMA-UE |
| Sun, 2023 [58]  (China) | EG: 34(21/13)  CG: 34(23/11) | 62.71 ± 11.40 /  65.50 ± 13.58 | NA | 0.10 ± 0.13 /  0.51 ± 0.73 | Screen-based virtual visual feedback | BCI-MI-FES | FES | 30min/d, 5d/w, 4w | high | ARAT / WMFT |
| Tang and Zhang, 2021 [59]  (China) | EG: 17(12/5)  CG: 17(11/6) | 49.93 ± 8.82 /  51.27 ± 8.56 | 32.05 ± 2.10 / 31.94 ± 2.56 | 1.72 ± 0.51 /  1.70 ± 0.50 | Screen-based visual abstract feedback + Auditory feedback | BCI-MI-FES | FES | 30min/d, 5d/w, 4w | high | FMA-UE / MBI |
| Wang et al, 2022 [60]  (China) | EG: 20(9/11)  CG: 20(12/8) | 69.05 ± 5.79 /  67.25 ± 4.78 | 16.45 ± 3.43 / 17.15 ± 3.83 | 1.40 ± 0.86 /  1.57 ± 0.77 | Screen-based virtual visual feedback + Auditory feedback | BCI-MI-FES | CRT | 20min/d, 5d/w, 4w | high | FMA-UE / WMFT / ARAT / MBI |
| Wang et al, 2023 [61]  (China) | EG: 60(47/13) CG: 60(45/15) | NA | 33.70 ±1 8.47 / 31.98 ± 15.51 | NA | Screen-based visual abstract feedback | BCI-MI-FES | CRT | 20min/d, 5d/w, 8w | moderate | FMA-UE / MBI |
| Wang et al, 2024 [62]  (China) | EG: 150(110/40)  CG: 146(115/31) | 59.67 ± 11.25 /  58.67 ± 10.50 | 28.00 ± 20.00 /  31.00 ± 26.67 | 0.48 ± 0.32 /  0.43 ± 0.25 | Screen-based visual abstract feedback | BCI-MI-FES | CRT | 30min/d, 5d/w, 4w | moderate | FMA-UE / ARAT / WMFT |
| Zhan et al, 2022 [63]  (China) | EG: 12  CG: 12 | NA | 19.17 ± 16.62 / 33.58 ± 18.89 | 38.33 ± 37.66 / 53.08 ± 46.88 | Screen-based visual feedback + Auditory feedback | BCI-MI-FES | FES | 40min/d, 3d/w, 4w | moderate | FMA-UE |
| Zhang, 2020 [64]  (China) | EG: 15(11/4)  CG: 15(7/8) | 60.93 ± 6.76 / 57.87 ± 8.61 | 17.00 ± 10.70 / 16.67 ± 8.55 | 3.02 ± 1.36 /  3.44 ± 1.55 | Screen-based virtual visual feedback | BCI-MI-FES | FES | 20min/d, 5d/w, 8w | moderate | FMA-UE / MBI |
| Zhang et al, 2025 [65]  (China) | EG: 20(13/7)  CG: 20(10/10) | 52.55 ± 11.08 / 51.25 ± 9.79 | 30.65 ± 12.08 / 30.20 ± 8.91 | 0.92 ± 0.39 /  0.76 ± 0.25 | Screen-based virtual visual feedback + Auditory feedback | BCI-MI-EFS | CRT | 30min/d, 5d/w, 3w | moderate | FMA-UE / WMFT / MBI |
| Zhou et al, 2025 [66]  (China) | EG: 20(11/9)  CG: 20(14/6) | 61.90 ± 13.30 / 66.10 ± 11.76 | 35.00 ± 6.52 / 36.75 ± 7.31 | NA | Screen-based virtual visual feedback + Auditory feedback | BCI-MI-FES | FES | 30min/d, 6d/w, 4w | high | FMA-UE / ARAT / WMFT / MBI |
| Brunner et al, 2024 [67]  (Denmark) | EG: 19(11/8)  CG: 21(14/7) | 56.00 ± 14.00 /  57.00 ± 11.00 | 3.33 ± 1.48 / 3.33 ± 1.48 | 1.02 ± 0.33 /  1.12 ± 0.53 | Screen-based virtual visual feedback + Auditory feedback | BCI-MI-FES + rTMS | rTMS | 60min/d | moderate | FMA-UE / ARAT |
| Tan, 2022 [10]  (China) | EG: 20(16/4)  CG: 20(15/5) | 55.55 ± 13.18 /  57.65 ± 9.61 | 33.60 ±1 1.54 / 31.30 ± 11.16 | 1.86 ± 11.23 /  2.10 ± 1.09 | Screen-based virtual visual feedback | BCI-MI-FES + rTMS | rTMS | 30min/d, 5d/w, 4w | high | FMA-UE / MBI |
| Cheng et al, 2020 [11]  (Singapore) | EG: 5(3/2)  CG: 5(2/3) | 62.40 ± 4.70 /  61.40 ± 4.50 | NA | 15.66 ± 9.92 /  29.25 ± 8.45 | Screen-based visual abstract feedback | BCI-MI-Soft robotic glove | Soft robotic glove | 30min/d, 3d/w, 6w | moderate | FMA-UE / ARAT |
| Ji et al, 2025 [68]  (China) | EG: 20(12/8)  CG: 19(15/4) | 61.75 ± 10.35 /  60.05 ± 14.35 | 25.80 ± 4.93 / 25.60 ± 4.92 | 1.63 ± 0.77 /  1.39 ± 0.80 | Screen-based visual abstract feedback | BCI-MI-Soft robotic glove | Soft robotic glove | 20-25min/d, 5d/w, 4w | moderate | FMA-UE / ARAT / MBI |
| Liu et al, 2025 [69]  (China) | EG: 15(5/10)  CG1: 15(4/11)  CG2: 15(5/10) | 61.60 ± 6.85 /  57.00 ± 9.59 /  56.13 ±10.83 | 25.87 ± 6.55 / 27.73 ± 5.50 | 2.61 ± 0.65 /  2.04 ± 0.74 /  1.73 ± 0.43 | Screen-based virtual visual feedback + Auditory feedback | BCI-MI-Soft robotic glove | CG1: Soft robotic glove  CG2: BCI-MI | 20min/d, 5d/w, 4w | moderate | FMA-UE / ARAT / MBI |
| Yang et al, 2023 [70]  (China) | EG: 25(18/7)  CG: 25(16/9) | 55.72 ± 11.55 / 50.92 ± 10.43 | NA | 2.21±1.25 /  2.77± 1.56 | Screen-based virtual visual feedback | BCI-MI-Soft robotic glove | Soft robotic glove | 20min/d, 5d/w, 2w | moderate | FMA-UE / MBI |
| Li et al, 2014 [12]  (China) | EG: 7  CG: 7 | 67.00 ± 4 .96 /  67.14 ± 5.96 | 13.57 ± 4.72 / 11.71 ± 2.63 | 2.69 ± 2.15 /  2.79 ± 2.00 | Screen-based visual abstract feedback + Auditory feedback | BCI-MI-TEAS | TEAS | 1~1.5h/d, 3d/w, 8w | moderate | FMA-UE / ARAT |
| Xin et al, 2022 [71]  (China) | EG: 60(39/21)  CG: 60(42/18) | 60.79 ± 11.24 / 59.62 ± 9.45 | 17.53 ± 3.61 / 17.93 ± 3.32 | 1.90 ± 0.89/  1.83 ± 0.80 | Screen-based virtual visual feedback | BCI-MI-TEAS | CRT | 60min/d, 5d/w, 4w | moderate | FMA-UE / MBI |
| Xia et al, 2025 [13]  (China) | EG: 18(12/6)  CG: 18(13/5) | 41.50 ± 13.40 / 39.30 ± 16.50 | 19.60 ± 9.40 / 17.10 ± 12.7 | 2.56 ± 3.04 /  1.90 ± 1.93 | Screen-based virtual visual feedback + Auditory feedback | BCI-SSVEP-Exoskeleton robot | CRT | 30min/d, 5d/w, 2w | high | FMA-UE |
| Guo et al, 2022 [14]  (China) | EG: 10(9/1)  CG: 10(8/2) | 60.20 ± 9.30 /  56.90 ± 6.10 | 14.90 ± 7.98 / 14.90 ± 5.23 | 12.50 ± 7.10 /  11.70 ± 5.40 | Screen-based visual abstract feedback | BCI-SSVEF-Soft robotic glove | SRG | 40min/d, 5d/w, 2w | high | FMA-UE / WMFT |
| Pan et al, 2026 [72]  (China) | EG: 10(6/4)  CG: 9(7/2) | 55.43 ± 10.15 / 53.67 ± 11.75 | 27.20 ± 12.33 / 15.50 ± 8.63 | 42.07 ± 106.53 / 4.92 ± 3.67 | Screen-based visual abstract feedback | BCI-SSVEP-Soft robotic glove | CRT | 3w | high | FMA-UE |

**Note:** E: Experimental group;; C: Control group; BCI: Brain-Computer interface; MI: Motor imagery; ERP: P300 event-related potentials; SSVEP: Steady-state visual evoked potentials; TEAS: transcutaneous electrical acupoint stimulation; FES: Functional electric stimulation; tDCS: Transcranial direct current stimulation; rTMS: Repetitive Transcranial magnetic stimulation; MIT: Mirror Image Training; CRT: Conventional rehabilitation training; min: Minutes; d: Days; w: Weeks; FMA-UE: Fugl-Meyer Assessment-Upper Extremity; ARAT: Action Research Arm Test, WMFT: Wolf Motor Function Test; MBI: Modified Barthel Index. NA: Not Applicable/Not Reported.

**Table S3.** Assessment of the transitivity assumption across treatment nodes for the outcomes.

**(A)** Fugl-Meyer Assessment of Upper Extremity

| **Node** | **Number of studies** | **Age, years** | **Baseline FMA-UE scores** | **Time from stroke onset (months)** | **Type of primary multi-sensory feedback (%)** | **Session duration (%)** | **Training sessions per week (%)** | **Duration of intervention (%)** |
| --- | --- | --- | --- | --- | --- | --- | --- | --- |
| BCI-ERP-FES | 1 | 51.80 ± 20.30 | 12.60 ± 20.11 | 51.33 ± 60.13 | Real-Human Imitation-Based Visual Feedback: 100% | NA | 2-3d/w: 100% | 4-5w: 100% |
| BCI-MI | 6 | 58.60 ± 10.23 | 11.51 ± 22.68 | 2.87 ± 1.95 | Real-human imitation-based visual feedback: 20%  Screen-based virtual visual feedback: 40%  Screen-based virtual visual feedback + Auditory feedback: 40% | ≤30min/d: 83.3%  60-90min/d: 16.7% | 2-3d/w: 40%  4-5d/w: 20%  6-7d/w: 40% | 2-3w: 60%  4-5w: 20%  6-8w: 20% |
| BCI-MI-End-effector robot | 7 | 54.41 ± 10.84 | 12.62 ± 8.21 | 7.79 ± 6.04 | Screen-based virtual visual feedback: 16.7%  Screen-based virtual visual feedback + Auditory feedback: 33.3%  Screen-based visual abstract feedback: 50% | ≤30min/d: 28.6%  30＜t＜60min/d: 14.3%  60-90min/d: 57.1% | 2-3d/w: 57.1%  4-5d/w: 42.9% | 2-3w: 14.3%  4-5w: 57.1%  6-8w: 28.6% |
| BCI-MI-End-effector robot + tDCS | 2 | 54.87 ± 9.29 | 16.61 ± 8.69 | 7.05 ± 10.42 | Screen-based virtual visual feedback + Auditory feedback: 50%  Screen-based visual abstract feedback: 50% | ≤30min/d: 100% | 4-5d/w: 100% | 4-5w: 100% |
| BCI-MI-Exoskeleton robot | 17 | 54.84 ± 11.01 | 10.03 ± 18.20 | 5.11 ± 4.28 | Auditory feedback: 17.6%  Real-human imitation-based visual feedback: 5.9%  Screen-based virtual visual feedback: 29.4%  Screen-based virtual visual feedback + Auditory feedback: 23.5%  Screen-based visual abstract feedback: 23.5% | ≤30min/d: 60%  30＜t＜60min/d: 13.3%  60-90: 26.7% | 2-3d/w: 12.5%  4-5d/w: 81.2%  6-7d/w: 6.2% | 2-3w: 35.3%  4-5w: 58.8%  6-8w: 5.9% |
| BCI-MI-Exoskeleton robot + tDCS | 2 | 59.00 ± 10.06 | 11.31 ± 3.40 | 0.55 ± 0.10 | Screen-based visual abstract feedback: 100% | ≤30min/d: 50%  30＜t＜60min/d: 50% | 4-5d/w: 100% | 4-5w: 100% |
| BCI-MI-FES | 24 | 57.41 ± 10.37 | 14.18 ± 13.19 | 5.07 ± 12.37 | Real-human imitation-based visual feedback: 13%  Screen-based virtual visual feedback: 13%  Screen-based virtual visual feedback + Auditory feedback: 56.5%  Screen-based visual abstract feedback: 17.4% | ≤30min/d: 75%  30＜t＜60min/d: 15%  60-90min/d: 10% | 2-3d/w: 18.2%  4-5d/w: 72.7%  6-7d/w: 9.1% | 2-3w: 13%  4-5w: 69.6%  6-8w: 17.4% |
| BCI-MI-FES + rTMS | 2 | 55.77 ± 13.59 | 10.96 ± 15.47 | 1.45 ± 0.52 | Screen-based virtual visual feedback: 50%  Screen-based virtual visual feedback + Auditory feedback: 50% | ≤30min/d: 50%  60-90min/d: 50% | 4-5d/w: 100% | 4-5w: 100% |
| BCI-MI-Soft robotic glove | 4 | 63.15 ± 22.23 | 17.24 ± 8.69 | 2.89 ± 2.46 | Screen-based virtual visual feedback: 20%  Screen-based virtual visual feedback + Auditory feedback: 40%  Screen-based visual abstract feedback: 40% | ≤30min/d: 100% | 2-3d/w: 20%  4-5d/w: 80% | 2-3w: 20%  4-5w: 60%  6-8w: 20% |
| BCI-MI-TEAS | 2 | 60.20 ± 11.27 | 31.04 ± 3.09 | 1.98 ± 1.07 | Screen-based virtual visual feedback: 50%  Screen-based virtual visual feedback + Auditory feedback: 50% | 60-90min/d: 100% | 2-3d/w: 50%  4-5d/w: 50% | 4-5w: 50%  6-8w: 50% |
| BCI-SSVEP-Exoskeleton robot | 1 | 41.50 ± 13.40 | 7.50 ± 7.50 | 2.56 ± 3.04 | Screen-based virtual visual feedback + Auditory feedback: 100% | ≤30min/d: 100% | 4-5d/w:100% | 2-3w: 100% |
| BCI-SSVEP-Soft robotic glove | 2 | 31.94 ± 9.73 | 12.19 ± 8.95 | 27.29 ± 75.50 | Screen-based virtual visual feedback: 50%  Screen-based visual abstract feedback: 50% | 30＜t＜60min/d: 100% | 4-5d/w: 100% | 2-3w: 100% |

**Note:** BCI: Brain-Computer interface; MI: Motor imagery; ERP: P300 event-related potentials; SSVEP: Steady-state visual evoked potentials; TEAS: transcutaneous electrical acupoint stimulation; FES: Functional electric stimulation; tDCS: Transcranial direct current stimulation; rTMS: Repetitive Transcranial magnetic stimulation; min: Minutes; d: Days; w: Weeks; FMA-UE: Fugl-Meyer assessment-upper extremity. NA: Not Applicable/Not Reported.

**(B)** Action Research Arm Test

| **Node** | **Number of studies** | **Age, years** | **Baseline ARAT scores** | **Time from stroke onset (months)** | **Type of primary multi-sensory feedback (%)** | **Session duration (%)** | **Training sessions per week (%)** | **Duration of intervention (%)** |
| --- | --- | --- | --- | --- | --- | --- | --- | --- |
| BCI-ERP-FES | 1 | 51.80 ± 20.30 | 10.00 ± 21.61 | 51.33 ± 60.13 | Real-Human Imitation-Based Visual Feedback: 100% | NA | 2-3d/w: 100% | 4-5w: 100% |
| BCI-MI | 2 | 51.66 ± 7.51 | 2.29 ± 17.10 | 6.09 ± 4.20 | Screen-based virtual visual feedback: 100% | ≤30min/d: 100% | 2-3d/w: 100% | 2-3w: 100% |
| BCI-MI-End-effector robot | 1 | 55.09 ± 11.42 | 13.46 ± 13.62 | 4.91 ± 0.83 | Screen-based virtual visual feedback + Auditory feedback: 100% | 60-90min/d: 100% | 4-5d/w: 100% | 2-3w: 100% |
| BCI-MI-End-effector robot + tDCS | 2 | 55.48 ± 8.64 | 13.95 ± 9.06 | 1.54 ± 0.85 | Screen-based virtual visual feedback + Auditory feedback: 66.7%  Screen-based visual abstract feedback: 33.3% | ≤30min/d: 100% | 4-5d/w: 100% | 4-5w: 100% |
| BCI-MI-Exoskeleton robot | 8 | 56.04 ± 10.57 | 9.03 ± 21.71 | 5.91 ± 4.57 | Auditory feedback: 14.3%  Screen-based virtual visual feedback: 14.3%  Screen-based virtual visual feedback + Auditory feedback: 14.3%  Screen-based visual abstract feedback: 57.1% | ≤30min/d: 42.9%  30＜t＜60min/d: 42.9% 60-90min/d: 14.3% | 2-3d/w: 12.5%  4-5d/w: 52.5%  6-7d/w: 25% | 2-3w: 37.5%  4-5w: 50%  6-8w: 12.5% |
| BCI-MI-Exoskeleton robot + tDCS | 1 | 59.00 ± 10.06 | 17.69 ± 7.78 | 0.55 ± 0.10 | Screen-based visual abstract feedback: 100% | ≤30min/d: 50%  30＜t＜60min/d: 50% | 4-5d/w: 100% | 4-5w: 100% |
| BCI-MI-FES | 6 | 60.80 ± 11.14 | 14.05 ± 13.05 | 0.96 ± 1.36 | Screen-based virtual visual feedback: 16.7%  Screen-based virtual visual feedback + Auditory feedback: 66.7%  Screen-based visual abstract feedback: 16.7% | ≤30min/d: 80%  30＜t＜60min/d: 20% | 4-5d/w: 83.3%  6-7d/w: 16.7% | 4-5w: 100% |
| BCI-MI-FES + rTMS | 1 | 56.00 ± 14.00 | 4.67 ± 8.15 | 1.02 ± 0.33 | Screen-based virtual visual feedback + Auditory feedback: 100% | 60-90min/d: 100% | NA | NA |
| BCI-MI-Soft robotic glove | 3 | 61.73 ± 8.21 | 11.91 ± 9.56 | 3.19 ± 2.86 | Screen-based virtual visual feedback + Auditory feedback: 50%  Screen-based visual abstract feedback: 50% | ≤30min/d: 100% | 2-3d/w: 25%  4-5d/w: 75% | 4-5w: 75%  6-8w: 25% |
| BCI-MI-TEAS | 1 | 67.00 ± 4.96 | 24.57 ± 6.02 | 2.69 ± 2.15 | Screen-based virtual visual feedback + Auditory feedback: 100% | 60-90min/d: 100% | 2-3d/w: 100% | 6-8w: 100% |

Note: BCI: Brain-Computer interface; MI: Motor imagery; ERP: P300 event-related potentials; SSVEP: Steady-state visual evoked potentials; TEAS: transcutaneous electrical acupoint stimulation; FES: Functional electric stimulation; tDCS: Transcranial direct current stimulation; rTMS: Repetitive Transcranial magnetic stimulation; min: Minutes; d: Days; w: Weeks; ARAT: Action research arm test. NA: Not Applicable/Not Reported.

**(C)** Wolf Motor Function Test

| **Node** | **Number of studies** | **Age, years** | **Baseline WMFT scores** | **Time from stroke onset (months)** | **Type of primary multi-sensory feedback (%)** | **Session duration (%)** | **Training sessions per week (%)** | **Duration of intervention (%)** |
| --- | --- | --- | --- | --- | --- | --- | --- | --- |
| BCI-MI-End-effector robot | 1 | 58.03 ± 7.19 | 22.00 ± 6.75 | 2.21 ± 0.27 | Screen-based virtual visual feedback + Auditory feedback: 100% | ≤30min/d: 100% | 4-5d/w: 100% | 4-5w: 100% |
| BCI-MI-Exoskeleton robot | 5 | 56.38 ± 11.11 | 17.17 ± 18.15 | 2.86 ± 2.43 | Screen-based virtual visual feedback: 25%  Screen-based virtual visual feedback + Auditory feedback: 50%  Screen-based visual abstract feedback: 25% | ≤30min/d: 20%  30＜t＜60min/d: 40%  60-90min/d: 40% | 4-5d/w: 80%  6-7d/w: 20% | 2-3w: 20%  4-5w: 80% |
| BCI-MI-FES | 9 | 59.66 ± 10.54 | 15.72 ± 13.76 | 0.91 ± 1.03 | Screen-based virtual visual feedback: 33.3%  Screen-based virtual visual feedback + Auditory feedback: 55.6%  Screen-based visual abstract feedback: 11.1% | ≤30min/d: 100% | 4-5d/w: 88.9%  6-7d/w: 11.1% | 2-3w: 22.2%  4-5w: 66.7%  6-8w: 11.1% |
| BCI-SSVEP-Soft robotic glove | 1 | 60.20 ± 9.30 | 5.10 ± 12.30 | 12.50 ± 7.10 | Screen-based visual abstract feedback: 100% | 30＜t＜60min/d: 100% | 4-5d/w: 100% | 2-3w: 100% |

**Note:** BCI: Brain-Computer interface; MI: Motor imagery; SSVEP: Steady-state visual evoked potentials; FES: Functional electric stimulation; WMFT: Wolf Motor Function Test; min: Minutes; d: Days; w: Weeks.

**(D)** Modified Barthel Index

| **Node** | **Number of studies** | **Age, years** | **Baseline MBI scores** | **Time from stroke onset (months)** | **Type of primary multi-sensory feedback (%)** | **Session duration (%)** | **Training sessions per week (%)** | **Duration of intervention (%)** |
| --- | --- | --- | --- | --- | --- | --- | --- | --- |
| BCI-MI | 2 | 57.40 ± 7.41 | 25.71 ± 11.76 | 3.46 ± 2.56 | Screen-based virtual visual feedback: 50%  Screen-based virtual visual feedback + Auditory feedback: 50% | ≤30min/d: 100% | 6-7d/w: 100% | 2-3w: 50%  6-8w: 50% |
| BCI-MI-End-effector robot | 2 | 57.24 ± 8.48 | 24.83 ± 8.37 | 2.93 ± 0.48 | Screen-based virtual visual feedback + Auditory feedback: 100% | ≤30min/d: 50%  60-90min/d: 50% | 4-5d/w: 100% | 2-3w: 50%  4-5w: 50% |
| BCI-MI-End-effector robot + tDCS | 2 | 55.48 ± 8.64 | 26.23 ± 10.24 | 1.54 ± 0.85 | Screen-based virtual visual feedback + Auditory feedback: 66.7%  Screen-based visual abstract feedback: 33.3% | ≤30min/d: 100% | 4-5d/w: 100% | 4-5w: 100% |
| BCI-MI-Exoskeleton robot | 6 | 52.94 ± 12.55 | 22.22 ± 16.69 | 3.22 ± 2.23 | Auditory feedback: 16.7%  Screen-based virtual visual feedback: 33.3%  Screen-based virtual visual feedback + Auditory feedback: 50% | ≤30min/d: 83.3%  60-90min/d: 16.7% | 4-5d/w: 100% | 2-3w: 33.3%  4-5w: 66.7% |
| BCI-MI-Exoskeleton robot + tDCS | 1 | 54.93 ± 8.73 | 25.00 ± 1.88 | 0.66 ± 0.12 | Screen-based visual abstract feedback: 100% | ≤30min/d: 100% | 4-5d/w: 100% | 4-5w: 100% |
| BCI-MI-FES | 13 | 56.07 ± 9.63 | 18.08 ± 14.00 | 2.39 ± 1.37 | Auditory feedback: 7.7%  Real-human imitation-based visual feedback: 15.4%  Screen-based virtual visual feedback: 15.4%  Screen-based virtual visual feedback + Auditory feedback: 53.8%  Screen-based visual abstract feedback: 7.7% | ≤30min/d: 100% | 4-5d/w: 83.3%  6-7d/w: 16.7% | 2-3w: 15.4%  4-5w: 53.8%  6-8w: 30.8% |
| BCI-MI-FES + rTMS | 1 | 55.55 ± 13.18 | 15.15 ± 15.93 | 1.86 ± 0.65 | Screen-based virtual visual feedback + Auditory feedback: 100% | ≤30min/d: 100% | 4-5d/w: 100% | 4-5w: 100% |
| BCI-MI-Soft robotic glove | 3 | 63.20 ± 22.83 | 19.84 ± 8.85 | 2.04 ± 0.92 | Screen-based virtual visual feedback: 25%  Screen-based virtual visual feedback + Auditory feedback: 50%  Screen-based visual abstract feedback: 25% | ≤30min/d: 100% | 4-5d/w: 100% | 2-3w: 25%  4-5w: 75% |
| BCI-MI-TEAS | 1 | 60.79 ± 11.24 | 26.98 ± 11.13 | 1.90 ± 0.89 | Screen-based virtual visual feedback: 100% | 60-90min/d: 100% | 4-5d/w: 100% | 4-5w: 100% |

**Note:** BCI: Brain-Computer interface; MI: Motor imagery; ERP: P300 event-related potentials; SSVEP: Steady-state visual evoked potentials; TEAS: Transcutaneous electrical acupoint stimulation; FES: Functional electric stimulation; tDCS: Transcranial direct current stimulation; rTMS: Repetitive Transcranial magnetic stimulation; MBI: Modified Barthel Index; min: Minutes; d: Days; w: Weeks.

**Figure S1.** Forest plots of pairwise meta-analysis for BCI-based intervention versus control groups.

**（A）**Fugl-Meyer Assessment-Upper Extremity

**(B)** Action Research Arm Test

**(C)** Wolf Motor Function Test

**(D)** Modified Barthel Index

**Figure S2.** Subgroup forest plots of pairwise meta-analysis stratified by BCI-based intervention versus control groups.

**（A）**Fugl-Meyer Assessment-Upper Extremity

**(B)** Action Research Arm Test

**(C)** Wolf Motor Function Test

**(D)** Modified Barthel Index

**Figure S3. Forest plot of subgroup analyses.**

**(A)** Fugl-Meyer Assessment-Upper Extremity

**(B)** Action Research Arm Test

**(C)** Wolf Motor Function Test

**(D)** Modified Barthel Index

**Table S4.** Meta-regression (n>10).

| Outcome | Variable | B | SE | t / z | 95%CI | P-value | Tau^2^ |
| --- | --- | --- | --- | --- | --- | --- | --- |
| FMA-UE | Publication year | 0.037 | 0.035 | 1.069 | [-0.031, 0.106] | 0.285 | 0.708 |
| **FMA-UE** | **Age** | **0.038** | **0.018** | **2.072** | **[0.002, 0.073]** | **0.038** | **0.697** |
| FMA-UE | Time from stroke onset | -0.018 | 0.012 | -1.592 | [-0.040, -0.004] | 0.112 | 0.781 |
| FMA-UE | Sex ratio | -0.013 | 0.094 | -0.139 | [-0.197, 0.171] | 0.900 | 0.796 |
| ARAT | Publication year | 0.057 | 0.038 | 1.516 | [-0.017, 0.131] | 0.130 | 0.203 |
| ARAT | Age | 0.026 | 0.027 | 0.956 | [-0.027,0.080] | 0.339 | 0.224 |
| ARAT | Time from stroke onset | -0.043 | 0.025 | -1.717 | [-0.093, 0.006] | 0.086 | 0.257 |
| ARAT | Sex ratio | -0.029 | 0.128 | -0.226 | [-0.280,0.222] | 0.822 | 0.257 |
| WMFT | Publication year | 0.018 | 0.080 | 0.223 | [-0.140, 0.175] | 0.824 | 0.076 |
| WMFT | Age | 0.020 | 0.018 | 1.080 | [-0.016, 0.056] | 0.280 | 0.076 |
| WMFT | Time from stroke onset | -0.012 | 0.042 | -0.286 | [-0.093, 0.070] | 0.775 | 0.096 |
| WMFT | Sex ratio | -0.020 | 0.101 | -0.194 | [-0.217, 0.178] | 0.846 | 0.076 |
| MBI | Publication year | 0.085 | 0.074 | 1.153 | [-0.060, 0.230] | 0.249 | 0.537 |
| MBI | Age | 0.012 | 0.029 | 0.406 | [-0.045,0.069] | 0.685 | 0.582 |
| MBI | Time from stroke onset | -0.076 | 0.077 | -0.989 | [-0.228, 0.075] | 0.323 | 0.626 |
| MBI | Sex ratio | 0.057 | 0.113 | 0.504 | [-0.164, 0.277] | 0.614 | 0.547 |

Sex ratio: Male/Female. Significant results are in bold.

**Abbreviations:** FMA-UE: Fugl-Meyer Assessment-Upper Extremity; ARAT: Action Research Arm Test; WMFT: Wolf Motor Function Test; MBI: Modified Barthel Index.

**Table S5.** Results of node-splitting analysis for evaluating local inconsistency.

**(A)** Fugl-Meyer Assessment-Upper Extremity

| **Comparison** | **TE** | **Lower** | **Upper** | ***P*-value** |
| --- | --- | --- | --- | --- |
| BCI-MI vs BCI-MI-Soft robotic glove | -5.538 | -10.401 | -0.675 | 0.026 |
| BCI-MI vs Control Group | 5.538 | 0.675 | 10.401 | 0.026 |
| BCI-MI-End-effector robot vs BCI-MI-End-effector robot + tDCS | 3.275 | 0.034 | 6.515 | 0.048 |
| BCI-MI-End-effector robot vs Control Group | -3.275 | -6.515 | -0.034 | 0.048 |
| BCI-MI-End-effector robot + tDCS vs Control Group | 3.275 | 0.034 | 6.515 | 0.048 |
| BCI-MI-Soft robotic glove vs Control Group | -5.538 | -10.401 | -0.675 | 0.026 |

**(B)** Action Research Arm Test

| **Comparison** | **TE** | **Lower** | **Upper** | **P-value** |
| --- | --- | --- | --- | --- |
| BCI-MI vs BCI-MI-Soft robotic glove | -2.257 | -12.313 | 7.798 | 0.660 |
| BCI-MI vs Control Group | 2.257 | -7.798 | 12.313 | 0.660 |
| BCI-MI-End-effector robot vs BCI-MI-End-effector robot + tDCS | -2.629 | -13.775 | 8.518 | 0.644 |
| BCI-MI-End-effector robot vs Control Group | 2.629 | -8.518 | 13.775 | 0.644 |
| BCI-MI-End-effector robot + tDCS vs Control Group | -2.629 | -13.775 | 8.518 | 0.644 |
| BCI-MI-Soft robotic glove vs Control Group | -2.257 | -12.313 | 7.798 | 0.660 |

**(C)** Modified Barthel Index

| **Comparison** | **TE** | **Lower** | **Upper** | ***P*-value** |
| --- | --- | --- | --- | --- |
| BCI-MI vs BCI-MI-Soft robotic glove | -7.004 | -14.109 | 0.101 | 0.053 |
| BCI-MI vs Control Group | 7.004 | -0.101 | 14.109 | 0.053 |
| BCI-MI-End-effector robot vs BCI-MI-End-effector robot + tDCS | -0.367 | -3.955 | 3.222 | 0.841 |
| BCI-MI-End-effector robot vs Control Group | 0.367 | -3.222 | 3.956 | 0.841 |
| BCI-MI-End-effector robot + tDCS vs Control Group | -0.367 | -3.955 | 3.222 | 0.841 |
| BCI-MI-Soft robotic glove vs Control Group | -7.004 | -14.109 | 0.101 | 0.053 |

**Abbreviations:** BCI: Brain-computer interface; MI: Motor imagery; tDCS: Transcranial direct current stimulation.

**Figure S4.** Ranking probability.

| **(A) Fugl-Meyer Assessment-upper Extremity**   | **(B) Action Research Arm Test**   |
| --- | --- |
| **(C) Wolf Motor Function Test**   | (**D) Modified Barthel Index**   |

**Abbreviations:** BCI: Brain-Computer interface; MI: Motor imagery; ERP: P300 event-related potentials; SSVEP: Steady-state visual evoked potentials; FES: Functional electric stimulation; tDCS: Transcranial direct current stimulation; rTMS: Repetitive Transcranial magnetic stimulation.

**Figure S5.** Funnel plot for pairwise meta-analysis to detect publication bias.

**(A)** Fugl-Meyer Assessment-Upper Extremity

**(B)** Action Research Arm Test

**(C)** Wolf Motor Function Test

**(D)** Modified Barthel Index

**Abbreviations:** BCI: Brain-computer interface; MI: Motor imagery; ERP: P300 event-related potentials; SSVEP: Steady-state visual evoked potentials; EE-RT: end-effector robotic training; Exo-RT: exoskeleton robotic training; SRGT: soft robotic gloves training; FES: Functional electric stimulation; tDCS: Transcranial direct current stimulation; rTMS: Repetitive transcranial magnetic stimulation.

**Figure S6.** Comparison-adjusted funnel plot for network meta-analysis to detect publication bias.

(A) Fugl-Meyer Assessment-Upper Extremity

**(B)** Action Research Arm Test

**(C)** Wolf Motor Function Test

**(D)** Modified Barthel Index

**Table S6.** Assessment of publication bias for pairwise meta-analyses.

| Outcome | Begg_Tau | Begg_P | Egger_Intercept | Egger_T | Egger_P |
| --- | --- | --- | --- | --- | --- |
| FMA-UE | 0.007 | 0.929 | -1.358 | -2.812 | 0.006 |
| ARAT | -0.025 | 0.867 | -0.016 | -0.036 | 0.972 |
| WMFT | 0.013 | 0.914 | -0.019 | -0.045 | 0.965 |
| MBI | 0.067 | 0.757 | 1.241 | 1.676 | 0.116 |

**Abbreviations:** FMA-UE: Fugl-Meyer Assessment-Upper Extremity; ARAT: Action Research Arm Test; WMFT: Wolf Motor Function Test; MBI: Modified Barthel Index.

**Table S7.** Assessment of publication bias and small-study effects for network meta-analysis.

| Outcome | Begg_Tau | Begg_P | Egger_Intercept | Egger_T | Egger_P |
| --- | --- | --- | --- | --- | --- |
| FMA-UE | -0.918 | 0.150 | -0.918 | -1.454 | 0.150 |
| ARAT | -1.171 | 0.079 | -1.171 | -1.827 | 0.079 |
| WMFT | -1.899 | 0.065 | -1.899 | -2.766 | 0.065 |
| MBI | -1.377 | 0.244 | -1.377 | -1.188 | 0.244 |

**Abbreviations:** FMA-UE: Fugl-Meyer Assessment-Upper Extremity; ARAT: Action Research Arm Test; WMFT: Wolf Motor Function Test; MBI: Modified Barthel Index.

**Figure S7.** Leave-one-out sensitivity analysis plot.

**(A)** Fugl-Meyer Assessment-Upper Extremity

**(B)** Action Research Arm Test

**(C)** Wolf Motor Function Test

(**D)** Modified Barthel Index

**Table S8.** Results of node-splitting analysis for evaluating local inconsistency after excluding high-risk studies.

**(A)** Fugl-Meyer Assessment-Upper Extremity

| **Comparison** | **TE** | **Lower** | **Upper** | ***P*-value** |
| --- | --- | --- | --- | --- |
| BCI-MI vs BCI-MI-Soft robotic glove | -3.898 | -13.887 | 6.092 | 0.444 |
| BCI-MI vs Control Group | 3.898 | 6.092 | 13.887 | 0.444 |
| BCI-MI-End-effector robot vs BCI-MI-End-effector robot + tDCS | 5.767 | -3.383 | 14.917 | 0.217 |
| BCI-MI-End-effector robot vs Control Group | -5.767 | -14.917 | 3.383 | 0.217 |
| BCI-MI-End-effector robot + tDCS vs Control Group | 5.767 | -3.383 | 14.917 | 0.217 |
| BCI-MI-Soft robotic glove vs Control Group | -3.898 | -13.887 | 6.092 | 0.444 |

**(B)** Action Research Arm Test

| **Comparison** | **TE** | **Lower** | **Upper** | **P-value** |
| --- | --- | --- | --- | --- |
| BCI-MI vs BCI-MI-Soft robotic glove | -1.348 | -12.311 | 9.616 | 0.810 |
| BCI-MI vs Control Group | 1.348 | -9.616 | 12.311 | 0.810 |
| BCI-MI-Soft robotic glove vs Control Group | -1.348 | -12.311 | 9.616 | 0.810 |

**(C)** Modified Barthel Index

| **Comparison** | **TE** | **Lower** | **Upper** | ***P*-value** |
| --- | --- | --- | --- | --- |
| BCI-MI vs BCI-MI-Soft robotic glove | -4.517 | -29.062 | 20.029 | 0.718 |
| BCI-MI vs Control Group | 4.517 | -20.029 | 29.062 | 0.718 |
| BCI-MI-Soft robotic glove vs Control Group | -4.517 | -29.062 | 20.029 | 0.718 |

**Abbreviations:** BCI: Brain-computer interface, MI: Motor imagery, tDCS: Transcranial direct current stimulation.

**Figure S8.** Ranking probability after excluding high-risk studies.

| **(A)** Fugl-Meyer Assessment-Upper Extremity   | **(B)** Action Research Arm Test   |
| --- | --- |
| **(C)** Wolf Motor Function Test   | (**D)** Modified Barthel Index   |

**Abbreviations:** BCI: Brain-computer interface; MI: Motor imagery; ERP: P300 event-related potentials; SSVEP: Steady-state visual evoked potentials; FES: Functional electric stimulation; tDCS: Transcranial direct current stimulation; rTMS: Repetitive transcranial magnetic stimulation.

**Table S9.** League table showing mean differences and their 95% CIs among BCI-based interventions after excluding high-risk studies.

**(A)** Fugl-Meyer Assessment-Upper Extremity

|  | BCI-MI | BCI-MI-End-effector robot | BCI-MI-End-effector robot + tDCS | BCI-MI-Exoskeleton robot | BCI-MI-Exoskeleton robot + tDCS | BCI-MI-FES | BCI-MI-FES + rTMS | BCI-MI-SRGT | BCI-MI-TEAS | Non-BCI-based interventions |
| --- | --- | --- | --- | --- | --- | --- | --- | --- | --- | --- |
| BCI-MI | — | — | — | — | — | — | — | — | — | — |
| BCI-MI-End-effector robot | 0.65 (-5.90 to 7.20) | — | — | — | — | — | — | — | — | — |
| BCI-MI-End-effector robot + tDCS | 1.12 (-5.35 to 7.60) | 0.47 (-3.53 to 4.47) | — | — | — | — | — | — | — | — |
| BCI-MI-Exoskeleton robot | 4.52 (-1.04 to 10.07) | 3.87 (-1.68 to 9.42) | 3.40 (-2.07 to 8.86) | — | — | — | — | — | — | — |
| BCI-MI-Exoskeleton robot + tDCS | 0.91 (-7.19 to 9.02) | 0.26 (-7.84 to 8.37) | -0.21 (-8.25 to 7.84) | -3.60 (-10.93 to 3.72) | — | — | — | — | — | — |
| BCI-MI-FES | 3.90 (-1.23 to 9.02) | 3.25 (-1.88 to 8.37) | 2.77 (-2.26 to 7.80) | -0.62 (-4.40 to 3.15) | 2.98 (-4.02 to 9.98) | — | — | — | — | — |
| BCI-MI-FES + rTMS | -0.37 (-13.11 to 12.38) | -1.02 (-13.76 to 11.73) | -1.49 (-14.20 to 11.22) | -4.88 (-17.15 to 7.38) | -1.28 (-14.89 to 12.33) | -4.26 (-16.34 to 7.82) | — | — | — | — |
| BCI-MI-SRGT | 5.07 (-0.04 to 10.17) | 4.41 (-1.52 to 10.35) | 3.94 (-1.92 to 9.80) | 0.55 (-4.28 to 5.37) | 4.15 (-3.47 to 11.77) | 1.17 (-3.15 to 5.49) | 5.43 (-7.02 to 17.88) | — | — | — |
| BCI-MI-TEAS | 10.76 (3.72 to 17.80) | 10.11 (3.07 to 17.14) | 9.64 (2.67 to 16.61) | 6.24 (0.12 to 12.37) | 9.84 (1.34 to 18.35) | 6.86 (1.12 to 12.60) | 11.12 (-1.88 to 24.13) | 5.69 (-0.78 to 12.17) | — | — |
| Non-BCI-based interventions | -1.88 (-6.51 to 2.76) | -2.53 (-7.15 to 2.10) | -3.00 (-7.52 to 1.53) | -6.39 (-9.46 to -3.32) | -2.79 (-9.44 to 3.86) | -5.77 (-7.97 to -3.57) | -1.51 (-13.39 to 10.37) | -6.94 (-10.66 to -3.22) | -12.63 (-17.93 to -7.33) | — |

**Note:** —: Not applicable. BCI: brain-computer interface; MI: motor imagery; TEAS: transcutaneous electrical acupoint stimulation; FES: functional electric stimulation; tDCS: transcranial direct current stimulation; rTMS: repetitive Transcranial magnetic stimulation.

**(B)** Action Research Arm Test

|  | BCI-MI | BCI-MI-End-effector robot | BCI-MI-End-effector robot + tDCS | BCI-MI-Exoskeleton robot | BCI-MI-Exoskeleton robot + tDCS | BCI-MI-FES | BCI-MI-FES + rTMS | BCI-MI-SRGT | BCI-MI-TEAS | Non-BCI-based interventions |
| --- | --- | --- | --- | --- | --- | --- | --- | --- | --- | --- |
| BCI-MI | — | — | — | — | — | — | — | — | — | — |
| BCI-MI-End-effector robot | 6.22 (-1.26 to 13.71) | — | — | — | — | — | — | — | — | — |
| BCI-MI-End-effector robot + tDCS | 9.52 (3.07 to 15.97) | 3.29 (-0.50 to 7.09) | — | — | — | — | — | — | — | — |
| BCI-MI-Exoskeleton robot | 4.67 (-1.88 to 11.21) | -1.56 (-8.13 to 5.02) | -4.85 (-10.22 to 0.52) | — | — | — | — | — | — | — |
| BCI-MI-Exoskeleton robot + tDCS | 4.40 (-5.35 to 14.15) | -1.82 (-11.59 to 7.95) | -5.11 (-14.12 to 3.89) | -0.27 (-9.34 to 8.81) | — | — | — | — | — | — |
| BCI-MI-FES | 4.72 (-1.60 to 11.04) | -1.50 (-7.86 to 4.85) | -4.80 (-9.90 to 0.30) | 0.05 (-5.16 to 5.26) | 0.32 (-8.60 to 9.23) | — | — | — | — | — |
| BCI-MI-FES + rTMS | 2.34 (-5.78 to 10.46) | -3.88 (-12.03 to 4.27) | -7.17 (-14.38 to 0.04) | -2.33 (-9.62 to 4.97) | -2.06 (-12.33 to 8.21) | -2.38 (-9.47 to 4.72) | — | — | — | — |
| BCI-MI-SRGT | 4.43 (-0.23 to 9.09) | -1.79 (-7.96 to 4.37) | -5.09 (-9.95 to -0.22) | -0.24 (-5.22 to 4.75) | 0.03 (-8.75 to 8.81) | -0.29 (-4.97 to 4.40) | 2.09 (-4.84 to 9.01) | — | — | — |
| BCI-MI-TEAS | 10.95 (1.65 to 20.25) | 4.73 (-4.60 to 14.05) | 1.44 (-7.08 to 9.95) | 6.28 (-2.30 to 14.87) | 6.55 (-4.67 to 17.77) | 6.23 (-2.19 to 14.65) | 8.61 (-1.23 to 18.45) | 6.52 (-1.76 to 14.80) | — | — |
| Non-BCI-based interventions | 0.67 (-4.60 to 5.94) | -5.55 (-10.86 to -0.24) | -8.84 (-12.56 to -5.13) | -4.00 (-7.87 to -0.12) | -3.73 (-11.93 to 4.47) | -4.05 (-7.53 to -0.56) | -1.67 (-7.85 to 4.51) | -3.76 (-6.89 to -0.63) | -10.28 (-17.94 to -2.62) | — |

**Note:** —: Not applicable. BCI: brain-computer interface; MI: motor imagery; TEAS: transcutaneous electrical acupoint stimulation; FES: functional electric stimulation; tDCS: transcranial direct current stimulation; rTMS: repetitive transcranial magnetic stimulation.

**(C)** Wolf Motor Function Test

|  | BCI-MI-Exoskeleton robot | BCI-MI-FES | Non-BCI-based interventions |
| --- | --- | --- | --- |
| BCI-MI-Exoskeleton robot | — | — | — |
| BCI-MI-FES | -5.94 (-13.46 to 1.58) | — | — |
| Non-BCI-based interventions | -11.41 (-17.63 to -5.19) | -5.47 (-9.70 to -1.23) | — |

**Note:** —: Not applicable. BCI: brain-computer interface; MI: motor imagery; FES: functional electric stimulation.

**(D)** Modified Barthel Index

|  | BCI-MI | BCI-MI-End-effector robot | BCI-MI-End-effector robot + tDCS | BCI-MI-Exoskeleton robot | BCI-MI-FES | BCI-MI-SRGT | BCI-MI-TEAS | Non-BCI-based interventions |
| --- | --- | --- | --- | --- | --- | --- | --- | --- |
| BCI-MI | — | — | — | — | — | — | — | — |
| BCI-MI-End-effector robot | 2.46 (-16.48 to 21.41) | — | — | — | — | — | — | — |
| BCI-MI-End-effector robot + tDCS | 4.31 (-11.88 to 20.50) | 1.85 (-8.00 to 11.69) | — | — | — | — | — | — |
| BCI-MI-Exoskeleton robot | 9.00 (-7.19 to 25.18) | 6.53 (-10.39 to 23.45) | 4.68 (-9.07 to 18.44) | — | — | — | — | — |
| BCI-MI-FES | 5.14 (-8.78 to 19.05) | 2.67 (-12.09 to 17.43) | 0.82 (-10.17 to 11.82) | -3.86 (-14.86 to 7.14) | — | — | — | — |
| BCI-MI-SRGT | 3.82 (-8.28 to 15.93) | 1.36 (-14.42 to 17.13) | -0.49 (-12.81 to 11.84) | -5.17 (-17.50 to 7.15) | -1.31 (-10.45 to 7.83) | — | — | — |
| BCI-MI-TEAS | 10.39 (-8.23 to 29.02) | 7.93 (-11.33 to 27.19) | 6.08 (-10.47 to 22.63) | 1.40 (-15.16 to 17.95) | 5.26 (-9.09 to 19.60) | 6.57 (-8.82 to 21.95) | — | — |
| Non-BCI-based interventions | -3.47 (-16.41 to 9.47) | -5.93 (-19.77 to 7.91) | -7.78 (-17.51 to 1.95) | -12.46 (-22.19 to -2.74) | -8.60 (-13.73 to -3.47) | -7.29 (-14.86 to 0.28) | -13.86 (-27.26 to -0.46) | — |

Note: —: Not applicable. BCI: brain-computer interface; MI: motor imagery; TEAS: transcutaneous electrical acupoint stimulation; tDCS: transcranial direct current stimulation; rTMS: repetitive transcranial magnetic stimulation.

**References**

1.	Ramirez-Nava AG, Mercado-Gutierrez JA, Quinzaños-Fresnedo J, Toledo-Peral C, Vega-Martinez G, Gutierrez MI, Pacheco-Gallegos MDR, Hernández-Arenas C, Gutiérrez-Martínez J. Functional electrical stimulation therapy controlled by a P300-based brain-computer interface, as a therapeutic alternative for upper limb motor function recovery in chronic post-stroke patients. A non-randomized pilot study. *Front Neurol.* 2023; 14:1221160.

2.	Pichiorri F, Morone G, Petti M, Toppi J, Pisotta I, Molinari M, Paolucci S, Inghilleri M, Astolfi L, Cincotti F, et al. Brain-computer interface boosts motor imagery practice during stroke recovery. *Ann Neurol.* 2015; 77(5):851-865.

3.	Hu YQ, Gao TH, Li J, Tao JC, Bai YL, Lu RR. Motor Imagery-Based Brain-Computer Interface Combined with Multimodal Feedback to Promote Upper Limb Motor Function after Stroke: A Preliminary Study. *Evid Based Complement Alternat Med.* 2021; 2021:1116126.

4.	Mihara M, Hattori N, Hatakenaka M, Yagura H, Kawano T, Hino T, Miyai I. Near-infrared spectroscopy-mediated neurofeedback enhances efficacy of motor imagery-based training in poststroke victims: a pilot study. *Stroke.* 2013; 44(4):1091-1098.

5.	Huang H. Impact of BCI-Based Upper Limb Rehabilitation on Hand Motor Recovery and Functional Restoration in Post-Stroke Patients. Master's thesis. Tianjin University of Sport. 2024. 10.27364/d.cnki.gttyy.2024.000259.

6.	Gao L, Chu F, Jia F, Chen J, Zhang M. Effect of brain-computer interface based on visual, auditory and motor feedback combined with transcranial di‐rect current stimulation on upper limb function in stroke patients. *Chin J Rehabil Theory Pract.* 2024; 30(02):202-209.

7.	Cantillo-Negrete J, Carino-Escobar RI, Carrillo-Mora P, Rodriguez-Barragan MA, Hernandez-Arenas C, Quinzaños-Fresnedo J, Hernandez-Sanchez IR, Galicia-Alvarado MA, Miguel-Puga A, Arias-Carrion O. Brain-Computer Interface Coupled to a Robotic Hand Orthosis for Stroke Patients' Neurorehabilitation: A Crossover Feasibility Study. *Front Hum Neurosci.* 2021; 15:656975.

8.	Chew E, Teo WP, Tang N, Ang KK, Ng YS, Zhou JH, Teh I, Phua KS, Zhao L, Guan C. Corrigendum: Using Transcranial Direct Current Stimulation to Augment the Effect of Motor Imagery-Assisted Brain-Computer Interface Training in Chronic Stroke Patients-Cortical Reorganization Considerations. *Front Neurol.* 2020; 11:605141.

9.	Biasiucci A, Leeb R, Iturrate I, Perdikis S, Al-Khodairy A, Corbet T, Schnider A, Schmidlin T, Zhang H, Bassolino M, et al. Brain-actuated functional electrical stimulation elicits lasting arm motor recovery after stroke. *Nat Commun.* 2018; 9(1):2421.

10.	Tan Y. Effect of repetitive transcranial magnetic stimulation combined with brain computer interface on upper limb motor function in stroke patients. Master's thesis. Hebei Medical University. 2022. 10.27111/d.cnki.ghyku.2022.000256.

11.	Cheng N, Phua KS, Lai HS, Tam PK, Tang KY, Cheng KK, Yeow RC, Ang KK, Guan C, Lim JH. Brain-Computer Interface-Based Soft Robotic Glove Rehabilitation for Stroke. *IEEE Trans Biomed Eng.* 2020; 67(12):3339-3351.

12.	Li M, Liu Y, Wu Y, Liu S, Jia J, Zhang L. Neurophysiological substrates of stroke patients with motor imagery-based Brain-Computer Interface training. *Int J Neurosci.* 2014; 124(6):403-415.

13.	Xia X, Kang X, Jia L, Wang Q, Zhang L, Zhang R, Wang Y, Wu X, Chen X, Liu X. Effects of MI-SSVEP-Based Brain-Computer Interface-Assisted Rehabilitation Training on Upper Limb Motor Function in Stroke Patients. *Chin J Stroke.* 2025; 20(09):1087-1096.

14.	Guo N, Wang X, Duanmu D, Huang X, Li X, Fan Y, Li H, Liu Y, Yeung EHK, To MKT, et al. SSVEP-Based Brain Computer Interface Controlled Soft Robotic Glove for Post-Stroke Hand Function Rehabilitation. *IEEE Trans Neural Syst Rehabil Eng.* 2022; 30:1737-1744.

15.	He J, Yuan Z, Quan L, Xi H, Guo J, Zhu D, Chen M, Yang B, Cui Z, Zhu S, et al. Multimodal assessment of a BCI system for stroke rehabilitation integrating motor imagery and motor attempts: a randomized controlled trial. *J Neuroeng Rehabil.* 2025; 22(1):185.

16.	He X, Wang Z, Xiao J, Wu L, Zhang H, Wang Y, Jia J. Comparative Study on the Efficacy of Rehabilitation Brain-Computer Interface versus MotorImagery Therapy in Upper Limb Motor Function Rehabilitation during the Flaccid ParalysisPhase of Stroke. *Chin J Stroke.* 2025; 20(10):1222-1228.

17.	Lu J, Wang P, Li H, Wang J, Li X, Wang C. Effect of brain computer interface combined with acupuncture therapy on motorand cognitive function in stroke patients. *Chin J Clin Res.* 2025; 38(05):725-729.

18.	Ang KK, Guan C, Phua KS, Wang C, Zhou L, Tang KY, Ephraim Joseph GJ, Kuah CW, Chua KS. Brain-computer interface-based robotic end effector system for wrist and hand rehabilitation: results of a three-armed randomized controlled trial for chronic stroke. *Front Neuroeng.* 2014; 7:30.

19.	Ang KK, Chua KS, Phua KS, Wang C, Chin ZY, Kuah CW, Low W, Guan C. A Randomized Controlled Trial of EEG-Based Motor Imagery Brain-Computer Interface Robotic Rehabilitation for Stroke. *Clin EEG Neurosci.* 2015; 46(4):310-320.

20.	Chen R, Cheng G, Zhang X, Li D, Lu P, Chen S, Xiao H. Randomized controlled trial of sequential motor imagery–based brain–computer interface and scalp acupuncture for post-stroke motor recovery with a multimodal neuroimaging case exploration. *Journal of Neurorestoratology.* 2026; 14(3).

21.	Foong R, Ang KK, Quek C, Guan C, Phua KS, Kuah CWK, Deshmukh VA, Yam LHL, Rajeswaran DK, Tang N, et al. Assessment of the Efficacy of EEG-Based MI-BCI With Visual Feedback and EEG Correlates of Mental Fatigue for Upper-Limb Stroke Rehabilitation. *IEEE Trans Biomed Eng.* 2020; 67(3):786-795.

22.	Lin BS, Chen JL, Hsu HC. Novel Upper-Limb Rehabilitation System Based on Attention Technology for Post-Stroke Patients: A Preliminary Study. *Ieee Access.* 2018; 6:2720-2731.

23.	Lu R, Pang Z, Gao T, He Z, Hu Y, Zhuang J, Zhang Q, Gao Z. Multisensory BCI promotes motor recovery via high-order network-mediated interhemispheric integration in chronic stroke. *BMC Med.* 2025; 23(1):380.

24.	Ming Z, Yu W, Fan J, Ling G, Fengming C, Wei T. Efficacy of kinesthetic motor imagery based brain computer interface combined with tDCS on upper limb function in subacute stroke. *Sci Rep.* 2025; 15(1):11829.

25.	Cantillo-Negrete J, Rodríguez-García ME, Carrillo-Mora P, Arias-Carrión O, Ortega-Robles E, Galicia-Alvarado MA, Valdés-Cristerna R, Ramirez-Nava AG, Hernandez-Arenas C, Quinzaños-Fresnedo J, et al. The ReHand-BCI trial: a randomized controlled trial of a brain-computer interface for upper extremity stroke neurorehabilitation. *Front Neurosci.* 2025; 19:1579988.

26.	Frolov AA, Mokienko O, Lyukmanov R, Biryukova E, Kotov S, Turbina L, Nadareyshvily G, Bushkova Y. Post-stroke Rehabilitation Training with a Motor-Imagery-Based Brain-Computer Interface (BCI)-Controlled Hand Exoskeleton: A Randomized Controlled Multicenter Trial. *Front Neurosci.* 2017; 11:400.

27.	Fu J, Chen S, Shu X, Jiang Z, Wei D, Jia J. Effects of Brain-Computer Interface Training on Motor Function Recovery in Patients with Post-Stroke Sensory lmpairment: An Exploratory Study. *Chinese Journal of Stroke.* 2022; 17(10):1051-1057.

28.	Fu J, Chen S, Shu X, Lin Y, Jiang Z, Wei D, Gao J, Jia J. Functional-oriented, portable brain-computer interface training for hand motor recovery after stroke: a randomized controlled study. *Front Neurosci.* 2023; 17:1146146.

29.	Li X, Wang L, Miao S, Yue Z, Tang Z, Su L, Zheng Y, Wu X, Wang S, Wang J, et al. Sensorimotor Rhythm-Brain Computer Interface With Audio-Cue, Motor Observation and Multisensory Feedback for Upper-Limb Stroke Rehabilitation: A Controlled Study. *Front Neurosci.* 2022; 16:808830.

30.	Liang S, Zhu Y, Wang W, Zhuang M, Xu D, Liu J, Tian S. Application of Brain-Computer Interface in the Rehabilitation of Upper Limb Motor Dysfunction in Stroke Patients. *Chinese Journal of Rehabilitation Medicine.* 2020; 35(02):185-188.

31.	Liu M, Li Z, Cao Y, He D, Song X. Effect of brain-computer interface training based on motor imagery on hand function for subacute stroke patients. *Chin J Rehabil Theory Pract.* 2023; 29(01):71-76.

32.	Liu J. Evaluation and Mechanism Study of Motor Imagery-Based Brain-Computer Interface Training on Upper-Limb Function Rehabilitation in Stroke Patients. Master's thesis. Guangdong Medical University. 2024.

33.	Liu L, Qin W, Le L, Qu X, Zhang Q, Nie Z. Application of motor imagery based brain computer interface in stroke patients with hand dysfunetion. *Chinese Journal of Rehabilitation.* 2024; 39(12):707-713.

34.	Lyukmanov RK, Aziatskaya GA, Mokienko OA, Varako NA, Kovyazina MS, Suponeva NA, Chernikova LA, Frolov AA, Piradov MA. [Post-stroke rehabilitation training with a brain-computer interface: a clinical and neuropsychological study]. *Zh Nevrol Psikhiatr Im S S Korsakova.* 2018; 118(8):43-51.

35.	Ramos-Murguialday A, Curado MR, Broetz D, Yilmaz Ö, Brasil FL, Liberati G, Garcia-Cossio E, Cho W, Caria A, Cohen LG, et al. Brain-Machine Interface in Chronic Stroke: Randomized Trial Long-Term Follow-up. *Neurorehabil Neural Repair.* 2019; 33(3):188-198.

36.	Várkuti B, Guan C, Pan Y, Phua KS, Ang KK, Kuah CW, Chua K, Ang BT, Birbaumer N, Sitaram R. Resting state changes in functional connectivity correlate with movement recovery for BCI and robot-assisted upper-extremity training after stroke. *Neurorehabil Neural Repair.* 2013; 27(1):53-62.

37.	Wu Q, Yue Z, Ge Y, Ma D, Yin H, Zhao H, Liu G, Wang J, Dou W, Pan Y. Brain Functional Networks Study of Subacute Stroke Patients With Upper Limb Dysfunction After Comprehensive Rehabilitation Including BCI Training. *Front Neurol.* 2019; 10:1419.

38.	Zanona AF, Piscitelli D, Seixas VM, Scipioni K, Bastos MSC, de Sá LCK, Monte-Silva K, Bolivar M, Solnik S, De Souza RF. Brain-computer interface combined with mental practice and occupational therapy enhances upper limb motor recovery, activities of daily living, and participation in subacute stroke. *Front Neurol.* 2022; 13:1041978.

39.	Zhang Z, Li C, Su M. A brain-computer interface can improve upper limb funetion after an ischemic stroke. *ChinJPhys Med Rehabil.* 2025; 47(4):300-306.

40.	Zheng J, Wu Z, Wang A, Zeng Y, Jiang R, Wang B. The clincal observation of XingnaoKaiqiao needling method combined withbrain-computer interface-and rehabilitation robot in treating the handdysfunction after stroke. *Journal of Gannan Medical University.* 2023; 43(01):53-58+71.

41.	Zhou G, Chen X, Tan H, Li Y, Li N, Cao Y, Wang H. The clinical efficacy of closed⁃loop rehabilitation therapy by brain⁃computer interface combined with exo⁃skeleton robotic hand for patients with hand dysfunction after cerebral infarction *The Journal of Practical Medicine.* 2024; 40(17):2395-2400.

42.	Wang C. Effect of brain computer interface combined with transcranial direct current stimulation on hand motor dysfunction in cerebral ischemic stroke. Master's thesis. Qinghai University. 2023. 10.27740/d.cnki.gqhdx.2023.000776.

43.	Chen X, Na J, Ma X, Jin Q, Zhang R, Zhang X. The Effect of Brain-Computer Interface Combined with Motor Imagery Training on Upper Limb Function Recovery in Patients with Ischemic Stroke. *Chinese Journal of Physical Medicine and Rehabilitation.* 2023; 45(3):222-225.

44.	Dun H, Guo H, Li H, Xu F, Zhu W. Application of mirror- therapy combined with brain-computer interfacete chnology in upper limb functional rehabilitation of patients after stroke. *Chongqing Medical Journal.* 2023; 52(18):2820-2824.

45.	Gao H, Hou S, Wang X, Zhu C. Effect of brain-computer interface on upper limb motor dysfunction in stroke patients based on functional near-infrared spectroscopy. *Chin J Rehabil Theory Pract.* 2025; 31(09):1066-1073.

46.	Hou S, Wang L, Wang C. lmprovement of upper limb and brain funetion in stroke patients through motor imagery brain computer interface training. *Chinese Journal of Rehabilitation Medicine.* 2024; 39(11):643-648.

47.	Kim T, Kim S, Lee B. Effects of Action Observational Training Plus Brain-Computer Interface-Based Functional Electrical Stimulation on Paretic Arm Motor Recovery in Patient with Stroke: A Randomized Controlled Trial. *Occup Ther Int.* 2016; 23(1):39-47.

48.	Kim MS, Park H, Kwon I, An KO, Kim H, Park G, Hyung W, Im CH, Shin JH. Efficacy of brain-computer interface training with motor imagery-contingent feedback in improving upper limb function and neuroplasticity among persons with chronic stroke: a double-blinded, parallel-group, randomized controlled trial. *J Neuroeng Rehabil.* 2025; 22(1):1.

49.	Krueger J, Krauth R, Reichert C, Perdikis S, Vogt S, Huchtemann T, Dürschmid S, Sickert A, Lamprecht J, Huremovic A, et al. Hebbian plasticity induced by temporally coincident BCI enhances post-stroke motor recovery. *Sci Rep.* 2024; 14(1):18700.

50.	Lee SH, Kim SS, Lee BH. Action observation training and brain-computer interface controlled functional electrical stimulation enhance upper extremity performance and cortical activation in patients with stroke: a randomized controlled trial. *Physiother Theory Pract.* 2022; 38(9):1126-1134.

51.	Liang T, Long Y, Lu L, Zhou J, Huang F, Huang L, Wu Y, Long Y, Wei X, Liu Z. Effect of rope-assisted proprioceptive neuromuscular facilitation combined with rope-assisted brain-computer in‐terface training on upper limb function in stroke patients with hemiplegia: a randomized controlled trial. *Chin J Rehabil Theory Pract.* 2024; 30(08):972-978.

52.	Liao W, Li J, Zhang X, Li C. Motor imagery brain-computer interface rehabilitation system enhances upper limb performance and improves brain activity in stroke patients: A clinical study. *Front Hum Neurosci.* 2023; 17:1117670.

53.	Liu X, Zhang W, Li W, Zhang S, Lv P, Yin Y. Effects of motor imagery based brain-computer interface on upper limb function and attention in stroke patients with hemiplegia: a randomized controlled trial. *BMC Neurol.* 2023; 23(1):136.

54.	Liu X. Effects of Motor Imagery based Brain-computerInterface on Upper Limb Function and Attention inStroke Patients with Hemiplegia. Master's thesis. North China University of Science and Technology. 2023. 10.27108/d.cnki.ghelu.2023.000062.

55.	Ma Z. Research on influence and brain mechanism of Brain computer interface and scalp acupuncture therapy on rehabilitation of motor recovery after stroke PhD thesis. Shanghai University of Traditional Chinese Medicine. 2021. 10.27320/d.cnki.gszyu.2021.000167.

56.	Miao Y, Chen S, Zhang X, Jin J, Xu R, Daly I, Jia J, Wang X, Cichocki A, Jung TP. BCI-Based Rehabilitation on the Stroke in Sequela Stage. *Neural Plast.* 2020; 2020:8882764.

57.	Rungsirisilp N, Chaiyawat P, Techataweesub S, Meesrisuk A, Wongsawat Y. Applying Action Observation During a Brain-Computer Interface on Upper Limb Recovery in Chronic Stroke Patients. *IEEE Access.* 2023; 11:4931-4943.

58.	Sun X. Investigation on functional reconstruction of patients with brain-computer interface combined with functional electrical stimulation in high plateaus cerebral ischemic stroke. Master's thesis. Qinghai University. 2023. 10.27740/d.cnki.gqhdx.2023.000412.

59.	Tang Q, Zhang T. Effects of Functional Electrical Stimulation Controlled by Brain-computer Interface on Upper Limb Dysfunctionin Stroke Patients. *Chin J Rehabil Theory Pract.* 2021; 27(07):802-806.

60.	Wang L, Zhang Y, Wang C, Sun C, Li M, Liu X. Clinical study of brain-computer interface based electrical stimulation training in improving upper limb dysfunctionof patients with stroke. *BME ＆ Clin Med.* 2022; 26(02):163-168.

61.	Wang P, Liu J, Wang L, Ma H, Mei X, Zhang A. Effects of brain-Computer interface combined with mindfulness therapy on rehabilitation of hemiplegic patients with stroke: a randomized controlled trial. *Front Psychol.* 2023; 14:1241081.

62.	Wang A, Tian X, Jiang D, Yang C, Xu Q, Zhang Y, Zhao S, Zhang X, Jing J, Wei N, et al. Rehabilitation with brain-computer interface and upper limb motor function in ischemic stroke: A randomized controlled trial. *Med.* 2024; 5(6):559-569.e554.

63.	Zhan G, Chen S, Ji Y, Xu Y, Song Z, Wang J, Niu L, Bin J, Kang X, Jia J. EEG-Based Brain Network Analysis of Chronic Stroke Patients After BCI Rehabilitation Training. *Front Hum Neurosci.* 2022; 16:909610.

64.	Zhang M. Effects of brain-computer interface technology on upper limb and hand function in stroke patients with hemiplegia. Master's thesis. Hebei Normal University. 2020. 10.27110/d.cnki.ghsfu.2020.000116.

65.	Zhang W, Liu X, Yan Y, Sun Z, Ge X, Li W, Lv P, Yin L. Clinical efficacy of motor imagery-based brain-computer interface collaborative training combined with conventional re⁃habilitation therapy on subacute stroke patients with hemiplegia. *Rehabil Med.* 2025; 35(02):146-151.

66.	Zhou Y, He J, Yang Z, Zhang L, Huang Y. Therapeutic effect of brain-computer interface (BCI)combined with functional electrical stimulation on upper limb motor function in stroke patients. *Chinese Journal of Rehabilitation.* 2025; 40(06):349-353.

67.	Brunner I, Lundquist CB, Pedersen AR, Spaich EG, Dosen S, Savic A. Brain computer interface training with motor imagery and functional electrical stimulation for patients with severe upper limb paresis after stroke: a randomized controlled pilot trial. *J Neuroeng Rehabil.* 2024; 21(1):10.

68.	Ji X, Lu X, Xu Y, Zhang W, Yang H, Yin C, Wang H, Ren C, Ji Y, Li Y, et al. Effects and neural mechanisms of a brain-computer interface-controlled soft robotic glove on upper limb function in patients with subacute stroke: a randomized controlled fNIRS study. *J Neuroeng Rehabil.* 2025; 22(1):171.

69.	Liu X, Gao L, Chu F, Chen J, Zhang M. Effect of brain-computer interface combined with upper limb rehabilitation robot on upper limb function ofstroke patients. *Chin J Rehabil Theory Pract.* 2025; 31(06):703-710.

70.	Yang Y, Wang H, Dong Y, Shi G, Li Q, Zhang J, Shi X. Effect of visual motion-induced brain computer interface technology on upper limb motor and cognitive functionof patients with stroke. *Chin J Rehabil Theory Pract.* 2023; 29(04):472-478.

71.	Xin H, Li P, Li X, Wang G. Effect of Low Frequency Transcutaneous Acupoint Electrical Stimulation Combined with Balance Acupuncture and Brain Computer Interface Technology on Upper Limb Motor Function of Stroke Patients. *J Clin Acupunct Mox.* 2022; 38(10):10-15.

72.	Pan X, Zhang R, Xia X, Cui H, Liu L, Chen X. Feasibility of a hybrid SSVEP-motor imagery BCI with robotic feedback for upper limb motor rehabilitation in stroke patients. *J Neurosci Methods.* 2026; 432:110780.
