# Supplementary material for: Comparative Effectiveness of Noninvasive Brain-Computer Interface–Based Interventions for Upper Limb Rehabilitation in Poststroke Hemiplegia: Systematic Review and Network Meta-Analysis of Randomized Controlled Trials
Source: J Med Internet Res. 2026 Aug 28;28:e92940. doi: 10.2196/92940 (PMC13524221; doi:10.2196/92940)
Supplement: Supplementary file 4 [file jmir-v28-e92940-s002.docx]

**Table S1. Search strategy**

**Date of search: from database inception to July 17, 2026.**

| **PubMed** | **Term** | **S.no** | **Search strategy** | **Results** |
| --- | --- | --- | --- | --- |
|  | #Population: Stroke | #1 | "Stroke"[Mesh] OR "Stroke Rehabilitation"[Mesh] OR "Cerebrovascular Disorders"[Mesh] OR "Brain Ischemia"[Mesh] OR "Brain Infarction"[Mesh] OR "Intracranial Hemorrhages"[Mesh] OR "Hemiplegia"[Mesh] OR "Paresis"[Mesh] | 494,075 |
|  |  | #2 | "stroke*"[Title/Abstract] OR "poststroke"[Title/Abstract] OR "post-stroke"[Title/Abstract] OR "cerebrovascular accident*"[Title/Abstract] OR "cerebral vascular accident*"[Title/Abstract] OR "brain vascular accident*"[Title/Abstract] OR "CVA"[Title/Abstract] OR "apoplex*"[Title/Abstract] OR "cerebral infarct*"[Title/Abstract] OR "brain infarct*"[Title/Abstract] OR "cerebral ischem*"[Title/Abstract] OR "brain ischem*"[Title/Abstract] OR "intracranial hemorrhag*"[Title/Abstract] OR "cerebral hemorrhag*"[Title/Abstract] OR "brain hemorrhag*"[Title/Abstract] OR "hemipleg*"[Title/Abstract] OR "hemipar*"[Title/Abstract] OR "paresis"[Title/Abstract] OR "motor impairment*"[Title/Abstract] OR "motor deficit*"[Title/Abstract] OR "motor dysfunction*"[Title/Abstract] OR "upper limb impairment*"[Title/Abstract] OR "upper extremity impairment*"[Title/Abstract] OR "upper limb dysfunction*"[Title/Abstract] OR "upper extremity dysfunction*"[Title/Abstract] OR "upper limb paresis"[Title/Abstract] OR "upper extremity paresis"[Title/Abstract] OR "arm dysfunction*"[Title/Abstract] OR "arm paresis"[Title/Abstract] | [523,851](https://pubmed.ncbi.nlm.nih.gov/?term=stroke*[Title/Abstract]+OR+poststroke[Title/Abstract]+OR+post-stroke[Title/Abstract]+OR+"cerebrovascular+accident*"[Title/Abstract]+OR+"cerebral+vascular+accident*"[Title/Abstract]+OR+"brain+vascular+accident*"[Title/Abstract]+OR+CVA[Title/Abstract]+OR+apoplex*[Title/Abstract]+OR+"cerebral+infarct*"[Title/Abstract]+OR+"brain+infarct*"[Title/Abstract]+OR+"cerebral+ischem*"[Title/Abstract]+OR+"brain+ischem*"[Title/Abstract]+OR+"intracranial+hemorrhag*"[Title/Abstract]+OR+"cerebral+hemorrhag*"[Title/Abstract]+OR+"brain+hemorrhag*"[Title/Abstract]+OR+hemipleg*[Title/Abstract]+OR+hemipar*[Title/Abstract]+OR+paresis[Title/Abstract]+OR+"motor+impairment*"[Title/Abstract]+OR+"motor+deficit*"[Title/Abstract]+OR+"motor+dysfunction*"[Title/Abstract]+OR+"upper+limb+impairment*"[Title/Abstract]+OR+"upper+extremity+impairment*"[Title/Abstract]+OR+"upper+limb+dysfunction*"[Title/Abstract]+OR+"upper+extremity+dysfunction*"[Title/Abstract]+OR+"upper+limb+paresis"[Title/Abstract]+OR+"upper+extremity+paresis"[Title/Abstract]+OR+"arm+dysfunction*"[Title/Abstract]+OR+"arm+paresis"[Title/Abstract]&sort=date) |
|  |  | #3 | #1 OR #2 | 757,227 |
|  | #Intervention: Non-invasive brain-computer interface | #4 | "Brain-Computer Interfaces"[Mesh] OR "Neurofeedback"[Mesh] | 8,087 |
|  |  | #5 | "non-invasive brain-computer interface*"[Title/Abstract] OR "brain-computer interface*"[Title/Abstract] OR "brain computer interface*"[Title/Abstract] OR "brain-machine interface*"[Title/Abstract] OR "brain machine interface*"[Title/Abstract] OR "brain-computer interaction*"[Title/Abstract] OR "brain machine interaction*"[Title/Abstract] OR "BCI"[Title/Abstract] OR "neural interface*"[Title/Abstract] OR "neural interface technolog*"[Title/Abstract] OR "hybrid brain-computer interface*"[Title/Abstract] OR "neuroprosthe*"[Title/Abstract] OR "neurofeedback"[Title/Abstract] OR "EEG neurofeedback"[Title/Abstract] OR "electroencephalograph*"[Title/Abstract] OR "EEG-based"[Title/Abstract] OR "fNIRS-based"[Title/Abstract] OR "functional near-infrared spectroscopy"[Title/Abstract] OR "motor imagery"[Title/Abstract] OR "movement imagery"[Title/Abstract] OR "P300 event-related potential"[Title/Abstract] OR "P300-based"[Title/Abstract] OR "SSVEP"[Title/Abstract] OR "steady-state visual evoked potential*"[Title/Abstract] OR "event-related potential*"[Title/Abstract] OR "ERP"[Title/Abstract] OR "visual evoked potential*"[Title/Abstract] | 128,078 |
|  |  | #6 | #4 OR #5 | 128,606 |
|  | #Study design: Randomized controlled trial | #7 | "randomized controlled trial"[Publication Type] OR "controlled clinical trial"[Publication Type] OR "random*"[Title/Abstract] OR "trial"[Title/Abstract] OR "placebo"[Title/Abstract] OR "clinical trial"[Title/Abstract] OR "controlled trial"[Title/Abstract] OR "allocat*"[Title/Abstract] OR "blind*"[Title/Abstract] OR "RCT"[Title/Abstract] | 2,712,952 |
|  | #8 | | #3 AND #6 AND #7 | 1,195 |
| **Embase** | **Term** | **S.no** | **Search strategy** | **Results** |
|  | #Population: Stroke | #1 | 'cerebrovascular disease'/exp OR 'stroke'/exp OR 'stroke patient'/exp OR 'ischemic stroke'/exp OR 'hemorrhagic stroke'/exp OR 'lacunar stroke'/exp OR 'stroke complication'/exp OR 'brain hemorrhage'/exp OR 'brain hematoma'/exp OR 'brain infarction'/exp OR 'brain ischemia'/exp OR 'intracranial hemorrhage'/exp OR 'stroke rehabilitation'/exp OR 'hemiplegia'/exp OR 'hemiparesis'/exp OR 'paresis'/exp | 1,169,699 |
|  |  | #2 | 'stroke*':ti,ab,kw OR 'poststroke':ti,ab,kw OR 'post-stroke':ti,ab,kw OR 'cerebrovascular accident*':ti,ab,kw OR 'cerebral vascular accident*':ti,ab,kw OR 'brain vascular accident*':ti,ab,kw OR 'cva':ti,ab,kw OR 'apoplex*':ti,ab,kw OR 'cerebral infarct*':ti,ab,kw OR 'brain infarct*':ti,ab,kw OR 'cerebral ischem*':ti,ab,kw OR 'brain ischem*':ti,ab,kw OR 'intracranial hemorrhag*':ti,ab,kw OR 'cerebral hemorrhag*':ti,ab,kw OR 'brain hemorrhag*':ti,ab,kw OR 'hemipleg*':ti,ab,kw OR hemipar*:ti,ab,kw OR 'paresis':ti,ab,kw OR 'motor impairment*':ti,ab,kw OR 'motor deficit*':ti,ab,kw OR 'motor dysfunction*':ti,ab,kw OR 'upper limb impairment*':ti,ab,kw OR 'upper extremity impairment*':ti,ab,kw OR 'upper limb dysfunction*':ti,ab,kw OR 'upper extremity dysfunction*':ti,ab,kw OR 'upper limb paresis':ti,ab,kw OR 'upper extremity paresis':ti,ab,kw OR 'arm dysfunction*':ti,ab,kw OR 'arm paresis':ti,ab,kw | 836,347 |
|  |  | #3 | #1 OR #2 | 1,35,804 |
|  | #Intervention: Non-invasive brain-computer interface | #4 | 'non-invasive brain computer interface'/exp OR 'brain computer interface'/exp OR 'brain machine interface'/exp OR 'neurofeedback'/exp | 18,815 |
|  |  | #5 | 'non-invasive brain-computer interface*':ti,ab,kw OR 'brain-computer interface*':ti,ab,kw OR 'brain computer interface*':ti,ab,kw OR 'brain-machine interface*':ti,ab,kw OR 'brain machine interface*':ti,ab,kw OR 'brain-computer interaction*':ti,ab,kw OR 'BCI':ti,ab,kw OR 'neural interface*':ti,ab,kw OR 'neural interface technolog*':ti,ab,kw OR 'hybrid brain-computer interface*':ti,ab,kw OR neuroprosthe*:ti,ab,kw OR neurofeedback:ti,ab,kw OR 'eeg neurofeedback':ti,ab,kw OR 'electroencephalograph*':ti,ab,kw OR 'EEG-based':ti,ab,kw OR 'fNIRS-based':ti,ab,kw OR 'functional near-infrared spectroscopy':ti,ab,kw OR 'motor imagery':ti,ab,kw OR 'movement imagery':ti,ab,kw OR 'P300 event-related potential':ti,ab,kw OR 'P300-based':ti,ab,kw OR 'SSVEP':ti,ab,kw OR 'steady-state visual evoked potential*':ti,ab,kw OR 'event-related potential*':ti,ab,kw OR 'ERP':ti,ab,kw OR 'visual evoked potential*':ti,ab,kw | 162,321 |
|  |  | #6 | #4 OR #5 | 165,325 |
|  | #Study design: Randomized controlled trial | #7 | 'randomized controlled trial'/exp OR 'controlled clinical trial'/exp OR 'randomization'/exp OR 'random*':ti,ab,kw OR 'trial':ti,ab,kw OR 'placebo':ti,ab,kw OR 'clinical trial':ti,ab,kw OR 'controlled trial':ti,ab,kw OR 'allocat*':ti,ab,kw OR 'blind*':ti,ab,kw OR 'RCT':ti,ab,kw | 4,195,126 |
|  | #8 | | #3 AND #6 AND #7 | 2,203 |
| **Cochrane Library** | **Term** | **S.no** | **Search strategy** | **Results** |
|  | #Population: Stroke | #1 | MH:"stroke" OR MH:"hemorrhagic strokes" OR MH:"brain infarction" OR MH:"ischemic stroke" OR MH:"stroke rehabilitation" OR MH:"brain injury, chronic" OR MH:"brain damage, chronic" OR MH:"ischemic attack, transient" OR MH:"hypoxia-ischemia, brain" OR MH:"brain infarction" OR MH:"brain ischemia" OR MH:"cerebrovascular disorders" OR MH:"hemiplegia" OR MH:"paresis" | 20,353 |
|  |  | #2 | "stroke":ti,ab,kw OR "poststroke":ti,ab,kw OR "post-stroke":ti,ab,kw OR "cerebrovascular accident":ti,ab,kw OR "cerebral vascular accident":ti,ab,kw OR "brain vascular accident":ti,ab,kw OR "apoplexy":ti,ab,kw OR "cerebral vascular accident":ti,ab,kw OR "CVA":ti,ab,kw OR "acute cerebrovascular accident":ti,ab,kw OR "cerebral infarction":ti,ab,kw OR "brain infarction":ti,ab,kw OR "cerebrovascular disorder":ti,ab,kw OR "brain vascular disorder":ti,ab,kw OR "intracranial vascular disorder":ti,ab,kw OR "intracranial vascular disease":ti,ab,kw OR "cerebrovascular disease":ti,ab,kw OR "cerebrovascular insufficiency":ti,ab,kw OR "cerebrovascular occlusion":ti,ab,kw OR "cerebral ischemia":ti,ab,kw OR "brain ischemia":ti,ab,kw OR "intracranial hemorrhagia":ti,ab,kw OR "cerebral hemorrhagia":ti,ab,kw OR "brain hemorrhagia":ti,ab,kw OR "hemiplegia":ti,ab,kw OR "hemiparesis":ti,ab,kw OR "paresis":ti,ab,kw OR "motor impairment":ti,ab,kw OR "motor deficit":ti,ab,kw OR "motor dysfunction":ti,ab,kw OR "upper limb impairment":ti,ab,kw OR "upper extremity impairment":ti,ab,kw OR "upper limb dysfunction":ti,ab,kw OR "upper extremity dysfunction":ti,ab,kw OR "upper limb paresis":ti,ab,kw OR "upper extremity paresis":ti,ab,kw OR "arm dysfunction":ti,ab,kw OR "arm paresis":ti,ab,kw | 97,466 |
|  |  | #3 | #1 OR #2 | 116,901 |
|  | #Intervention: Non-invasive brain-computer interface | #4 | MH:"Brain-Computer Interfaces" OR MH:"Neurofeedback" | 211 |
|  |  | #5 | "non-invasive brain-computer interface":ti,ab,kw OR "brain-computer interface":ti,ab,kw OR "brain computer interface":ti,ab,kw OR "brain-machine interface":ti,ab,kw OR "brain machine interface":ti,ab,kw OR "brain-computer interaction":ti,ab,kw OR "BCI":ti,ab,kw OR "neural interface":ti,ab,kw OR "neural interface technology":ti,ab,kw OR "hybrid brain-computer interface":ti,ab,kw OR "neuroprosthesis":ti,ab,kw OR "neurofeedback":ti,ab,kw OR "EEG neurofeedback":ti,ab,kw OR "electroencephalograph":ti,ab,kw OR "EEG-based":ti,ab,kw OR "fNIRS-based":ti,ab,kw OR "functional near-infrared spectroscopy":ti,ab,kw OR "motor imagery":ti,ab,kw OR "movement imagery":ti,ab,kw OR "P300 event-related potential":ti,ab,kw OR "P300-based":ti,ab,kw OR "SSVEP":ti,ab,kw OR "steady-state visual evoked potential":ti,ab,kw OR "event-related potential":ti,ab,kw OR "ERP":ti,ab,kw OR "visual evoked potential":ti,ab,kw | 8,164 |
|  |  | #6 | #4 OR #5 | 28,081 |
|  | #Study design: Randomized controlled trial | #7 | "randomized controlled trial":ti,ab,kw OR "controlled clinical trial":ti,ab,kw OR "clinical trial":ti,ab,kw OR "controlled trial":ti,ab,kw OR "random":ti,ab,kw OR "randomly":ti,ab,kw OR "randomization":ti,ab,kw OR "random allocation":ti,ab,kw OR "allocation":ti,ab,kw OR "placebo":ti,ab,kw OR trial:ti,ab,kw OR "blind":ti,ab,kw OR "single blind":ti,ab,kw OR "double blind":ti,ab,kw OR "RCT":ti,ab,kw | 1,630,443 |
|  | #8 | | #3 AND #6 AND #7 | 755 |
| **Web of Science** | **Term** | **S.no** | **Search strategy** | **Results** |
|  | #Population: Stroke | #1 | TS=("stroke*" OR "stroke rehabilitation" OR "cerebrovascular disorder*" OR "poststroke" OR "post-stroke" OR "cerebrovascular accident*" OR "cerebral vascular accident*" OR "brain vascular accident*" OR "CVA" OR "apoplex*" OR "cerebral infarct*" OR "brain infarct*" OR "cerebral ischem*" OR "brain ischem*" OR "intracranial hemorrhag*" OR "cerebral hemorrhag*" OR "brain hemorrhag*" OR "hemipleg*" OR "hemipar*" OR paresis OR "motor impairment*" OR "motor deficit*" OR "motor dysfunction*" OR "upper limb impairment*" OR "upper extremity impairment*" OR "upper limb dysfunction*" OR "upper extremity dysfunction*" OR "upper limb paresis" OR "upper extremity paresis" OR "arm dysfunction*" OR "arm paresis") | 681,872 |
|  | #Intervention: Non-invasive brain-computer interface | #2 | TS=("non-invasive brain-computer interface*" OR "brain-computer interface*" OR "brain computer interface*" OR "brain-machine interface*" OR "brain machine interface*" OR "brain-computer interaction*" OR "brain machine interaction*" OR "BCI" OR "neural interface*" OR "neural interface technolog*" OR "hybrid brain-computer interface*" OR "neuroprosthe*" OR "neurofeedback" OR "EEG neurofeedback" OR "electroencephalograph*" OR "EEG-based" OR "fNIRS-based" OR "functional near-infrared spectroscopy" OR "motor imagery" OR "movement imagery" OR "P300 event-related potential" OR "P300-based" OR "SSVEP" OR "steady-state visual evoked potential*" OR "event-related potential*" OR "ERP" OR "visual evoked potential*") | 179,795 |
|  | #Study design: Randomized controlled trial | #3 | TS=("randomized controlled trial" OR "controlled clinical trial" OR random* OR "trial" OR "placebo" OR "clinical trial" OR "controlled trial" OR "allocat*" OR "blind*" OR "RCT") | 4,628,964 |
|  | #4 | | #1 AND #2 AND #3 | 1,592 |
| **MEDLINE & CINAHL** | **Term** | **S.no** | **Search strategy** | **Results** |
|  | #Population: Stroke | #1 | MH "Stroke+" OR MH "Stroke Rehabilitation+" OR MH "Cerebrovascular Disorders+" OR MH "Brain Ischemia+" OR MH "Brain Infarction+" OR MH "Intracranial Hemorrhages+" OR MH "Hemiplegia+" OR MH "Paresis+" | 633,025 |
|  |  | #2 | TI ("stroke*" OR "poststroke" OR "post-stroke" OR "cerebrovascular accident*" OR "cerebral vascular accident*" OR "brain vascular accident*" OR CVA OR apoplex* OR "cerebral infarct*" OR "brain infarct*" OR "cerebral ischem*" OR "brain ischem*" OR "intracranial hemorrhag*" OR "cerebral hemorrhag*" OR "brain hemorrhag*" OR hemipleg* OR hemipar* OR paresis OR "motor impairment*" OR "motor deficit*" OR "motor dysfunction*" OR "upper limb impairment*" OR "upper extremity impairment*" OR "upper limb dysfunction*" OR "upper extremity dysfunction*" OR "upper limb paresis" OR "upper extremity paresis" OR "arm dysfunction*" OR "arm paresis") OR AB ("stroke*" OR "poststroke" OR "post-stroke" OR "cerebrovascular accident*" OR "cerebral vascular accident*" OR "brain vascular accident*" OR CVA OR apoplex* OR "cerebral infarct*" OR "brain infarct*" OR "cerebral ischem*" OR "brain ischem*" OR "intracranial hemorrhag*" OR "cerebral hemorrhag*" OR "brain hemorrhag*" OR hemipleg* OR hemipar* OR paresis OR "motor impairment*" OR "motor deficit*" OR "motor dysfunction*" OR "upper limb impairment*" OR "upper extremity impairment*" OR "upper limb dysfunction*" OR "upper extremity dysfunction*" OR "upper limb paresis" OR "upper extremity paresis" OR "arm dysfunction*" OR "arm paresis") | 666,977 |
|  |  | #3 | #1 OR #2 | 961,901 |
|  | #Intervention: Non-invasive brain-computer interface | #4 | MH "Brain-Computer Interfaces+" OR MH "Neurofeedback+" | 8,775 |
|  |  | #5 | TI ("non-invasive brain-computer interface*" OR "brain-computer interface*" OR "brain computer interface*" OR "brain-machine interface*" OR "brain machine interface*" OR "brain-computer interaction*" OR "brain machine interaction*" OR "BCI" OR "neural interface*" OR "neural interface technolog*" OR "hybrid brain-computer interface*" OR "neuroprosthe*" OR "neurofeedback" OR "EEG neurofeedback" OR "electroencephalograph*" OR "EEG-based" OR "fNIRS-based" OR "functional near-infrared spectroscopy" OR "motor imagery" OR "movement imagery" OR "P300 event-related potential" OR "P300-based" OR "SSVEP" OR "steady-state visual evoked potential*" OR "event-related potential*" OR "ERP" OR "visual evoked potential*") OR AB ("non-invasive brain-computer interface*" OR "brain-computer interface*" OR "brain computer interface*" OR "brain-machine interface*" OR "brain machine interface*" OR "brain-computer interaction*" OR "brain machine interaction*" OR "BCI" OR "neural interface*" OR "neural interface technolog*" OR "hybrid brain-computer interface*" OR "neuroprosthe*" OR "neurofeedback" OR "EEG neurofeedback" OR "electroencephalograph*" OR "EEG-based" OR "fNIRS-based" OR "functional near-infrared spectroscopy" OR "motor imagery" OR "movement imagery" OR "P300 event-related potential" OR "P300-based" OR "SSVEP" OR "steady-state visual evoked potential*" OR "event-related potential*" OR "ERP" OR "visual evoked potential*") | 130,762 |
|  |  | #6 | #4 OR #5 | 131,561 |
|  | #Study design: Randomized controlled trial | #7 | PT "randomized controlled trial" OR PT "controlled clinical trial" OR TI ("random*" OR "trial" OR "placebo" OR "allocat*" OR "blind*" OR "RCT") OR AB ("random*" OR "trial" OR "placebo" OR "allocat*" OR "blind*" OR "RCT") | 3,941,315 |
|  | #8 | | #3 AND #6 AND #7 | 1,634 |
| **Wiley Online Library** | **Term** | **S.no** | **Search strategy** | **Results** |
|  | #Population: Stroke | #1 | "stroke*" OR "stroke rehabilitation" OR "cerebrovascular disorder*" OR "poststroke" OR "post-stroke" OR "cerebrovascular accident*" OR "cerebral vascular accident*" OR "brain vascular accident*" OR "CVA" OR "apoplex*" OR "cerebral infarct*" OR "brain infarct*" OR "cerebral ischem*" OR "brain ischem*" OR "intracranial hemorrhag*" OR "cerebral hemorrhag*" OR "brain hemorrhag*" OR "hemipleg*" OR "hemipar*" OR paresis OR "motor impairment*" OR "motor deficit*" OR "motor dysfunction*" OR "upper limb impairment*" OR "upper extremity impairment*" OR "upper limb dysfunction*" OR "upper extremity dysfunction*" OR "upper limb paresis" OR "upper extremity paresis" OR "arm dysfunction*" OR "arm paresis" in Abstract | 41,871 |
|  | #Intervention: Non-invasive brain-computer interface | #2 | "non-invasive brain-computer interface*" OR "brain-computer interface*" OR "brain computer interface*" OR "brain-machine interface*" OR "brain machine interface*" OR "brain-computer interaction*" OR "brain machine interaction*" OR "BCI" OR "neural interface*" OR "neural interface technolog*" OR "hybrid brain-computer interface*" OR "neuroprosthe*" OR "neurofeedback" OR "EEG neurofeedback" OR "electroencephalograph*" OR "EEG-based" OR "fNIRS-based" OR "functional near-infrared spectroscopy" OR "motor imagery" OR "movement imagery" OR "P300 event-related potential" OR "P300-based" OR "SSVEP" OR "steady-state visual evoked potential*" OR "event-related potential*" OR "ERP" OR "visual evoked potential*" in Abstract | 8,789 |
|  | #Study design: Randomized controlled trial | #3 | "randomized controlled trial" OR "controlled clinical trial" OR random* OR "trial" OR "placebo" OR "clinical trial" OR "controlled trial" OR "allocat*" OR "blind*" OR "RCT" in Abstract | 392,519 |
|  | #4 | | #1 AND #2 AND #3 | 48 |
| **CNKI(Chinese database)** | **Term** | **S.no** | **Search strategy** | **Results** |
|  | #Population: Stroke | #1 | SU=('卒中' + '中风' + '脑卒中' + '脑血管意外' + '脑梗死' + '脑梗塞' + '缺血性卒中' + '出血性卒中' + '脑出血' + '蛛网膜下腔出血' + '脑缺血' + '偏瘫' + '半身不遂' + '偏身瘫痪' + '肢体瘫痪' + '上肢瘫痪' + '偏身轻瘫' + '运动功能障碍' + '运动障碍' + '上肢功能障碍' + '上肢运动障碍') | 537,463 |
|  | #Intervention: Non-invasive brain-computer interface | #2 | SU=('脑机接口' + '脑-机接口' + '脑机交互' + '脑机交互技术' + '脑机器接口' + '脑-机器接口' + 'BCI' + '神经接口' + '神经反馈' + '运动想象' +' P300' + '稳态视觉诱发电位' + 'SSVEP' + '事件相关电位' + '功能性近红外光谱' + '近红外脑功能成像') | 50,847 |
|  | #Intervention: Non-invasive brain-computer interface | #3 | SU=('随机' + '随机对照' + '随机对照试验' + '对照试验' + '临床试验' + '随机分组' + '随机分配' + '盲法') | 421,712 |
|  | #4 | | #1 AND #2 AND #3 | 58 |
| **WanFang(Chinese database)** | **Term** | **S.no** | **Search strategy** | **Results** |
|  | #Population: Stroke | #1 | 主题:("卒中" OR "中风" OR "脑卒中" OR "脑血管意外" OR "脑梗死" OR "脑梗塞" OR "缺血性卒中" OR "出血性卒中" OR "脑出血" OR "蛛网膜下腔出血" OR "脑缺血" OR "偏瘫" OR "半身不遂" OR "偏身瘫痪" OR "肢体瘫痪" OR "上肢瘫痪" OR "偏身轻瘫" OR "运动功能障碍" OR "运动障碍" OR "上肢功能障碍" OR "上肢运动障碍") | 1,021,024 |
|  | #Intervention: Non-invasive brain-computer interface | #2 | 主题:("脑机接口" OR "脑-机接口" OR "脑机交互" OR "脑机交互技术" OR "脑机器接口" OR "脑-机器接口" OR "神经接口" OR "神经反馈" OR "脑电神经反馈" OR "脑电图" OR "脑电信号" OR "运动想象" OR "P300" OR "稳态视觉诱发电位" OR "SSVEP" OR "事件相关电位" OR "功能性近红外光谱" OR "近红外脑功能成像") | 79,772 |
|  | #Study design: Randomized controlled trial | #3 | 主题:("随机" OR "随机对照" OR "随机对照试验" OR "对照试验" OR "临床试验" OR "随机分组" OR "随机分配" OR "盲法") | 3,885,227 |
|  | #4 | | #1 AND #2 AND #3 | 1,531 |
| **Sinomed(Chinese database)** | **Term** | **S.no** | **Search strategy** | **Results** |
|  | #Population: Stroke | #1 | "中风"[不加权:扩展] OR "偏瘫"[不加权:扩展] | 69,958 |
|  |  | #2 | ("卒中"[摘要:智能] OR "中风"[摘要:智能] OR "脑卒中"[摘要:智能] OR "脑血管意外"[摘要:智能] OR "脑梗死"[摘要:智能] OR "脑梗塞"[摘要:智能] OR "缺血性卒中"[摘要:智能] OR "出血性卒中"[摘要:智能] OR "脑出血"[摘要:智能] OR "蛛网膜下腔出血"[摘要:智能] OR "脑缺血"[摘要:智能] OR "偏瘫"[摘要:智能] OR "半身不遂"[智能] OR "偏身瘫痪"[智能] OR "肢体瘫痪"[摘要:智能] OR "上肢瘫痪"[摘要:智能] OR "偏身轻瘫"[摘要:智能] OR "运动功能障碍"[摘要:智能] OR "上肢功能障碍"[摘要:智能] OR "上肢运动障碍"[摘要:智能]) | 808,920 |
|  |  | #3 | #1 OR #2 | 827,244 |
|  | #Intervention: Non-invasive brain-computer interface | #4 | ("脑机接口"[摘要:智能] OR "脑-机接口"[摘要:智能] OR "脑机交互"[摘要:智能] OR "脑机交互技术"[摘要:智能] OR "脑机器接口"[摘要:智能] OR "脑-机器接口"[摘要:智能] OR "BCI"[摘要:智能] OR "神经接口"[摘要:智能] OR "神经反馈"[摘要:智能] OR "运动想象"[摘要:智能] OR "P300"[摘要:智能] OR "稳态视觉诱发电位"[摘要:智能] OR "SSVEP"[摘要:智能] OR "事件相关电位"[摘要:智能] OR "功能性近红外光谱"[摘要:智能] OR "近红外脑功能成像"[摘要:智能]) | 96,063 |
|  | #Study design: Randomized controlled trial | #5 | "随机对照试验"[不加权:扩展] | 205,519 |
|  |  | #6 | ("随机"[摘要:智能] OR "随机对照"[摘要:智能] OR "随机对照试验"[摘要:智能] OR "对照试验"[智能] OR "临床试验"[摘要:智能] OR "随机分组"[摘要:智能] OR "随机分配"[摘要:智能] OR "盲法"[摘要:智能]) | 2,236,009 |
|  |  | #7 | #5 OR #6 | 2,648,663 |
|  | #8 | | #3 AND #4 AND #7 | 1,212 |
| **VIP(Chinese database)** | **Term** | **S.no** | **Search strategy** | **Results** |
|  | #Population: Stroke | #1 | M = (卒中 OR 中风 OR 脑卒中 OR 脑血管意外 OR 脑梗死 OR 脑梗塞 OR 缺血性卒中 OR 出血性卒中 OR 脑出血 OR 蛛网膜下腔出血 OR 脑缺血 OR 偏瘫 OR 半身不遂 OR 偏身瘫痪 OR 肢体瘫痪 OR 上肢瘫痪 OR 偏身轻瘫 OR 运动功能障碍 OR 运动障碍 OR 上肢功能障碍 OR 上肢运动障碍) | 458,056 |
|  | #Intervention: Non-invasive brain-computer interface | #2 | M = (脑机接口 OR 脑-机接口 OR 脑机交互 OR 脑机交互技术 OR 脑机器接口 OR 脑-机器接口 OR BCI OR 神经接口 OR 神经反馈 OR 运动想象 OR P300 OR 稳态视觉诱发电位 OR SSVEP OR 事件相关电位 OR 功能性近红外光谱 OR 近红外脑功能成像) | 882,750 |
|  | #Study design: Randomized controlled trial | #3 | M = (随机 OR 随机对照 OR 随机对照试验 OR 对照试验 OR 临床试验 OR 随机分组 OR 随机分配 OR 盲法) | 175,557 |
|  | #4 | | #1 AND #2 AND #3 | 1,443 |
